# Phylogenetic revision of *Minyomerus* Horn, 1876 sec. Jansen & Franz, 2015 (Coleoptera, Curculionidae) using taxonomic concept annotations and alignments

**DOI:** 10.3897/zookeys.528.6001

**Published:** 2015-10-20

**Authors:** M. Andrew Jansen, Nico M. Franz

**Affiliations:** 1 School of Life Sciences, PO Box 874501, Arizona State University, Tempe, AZ 85287-4501

**Keywords:** Biogeography, cladistics, concept taxonomy, desert, Entiminae, *Minyomerus*, new species, parthenogenesis, *Piscatopus*, provenance, revision, weevils

## Abstract

This contribution adopts the taxonomic concept annotation and alignment approach. Accordingly, and where indicated, previous and newly inferred meanings of taxonomic names are individuated *according to* one specific source. Articulations among these concepts and pairwise, logically consistent alignments of original and revisionary classifications are also provided, in addition to conventional nomenclatural provenance information. A phylogenetic revision of the broad-nosed weevil genera *Minyomerus* Horn, 1876 sec. O’Brien & Wibmer (1982), and *Piscatopus* Sleeper, 1960 sec. O’Brien & Wibmer (1982) (Curculionidae [non-focal]: Entiminae [non-focal]: Tanymecini [non-focal]) is presented. Prior to this study, *Minyomerus* sec. O’Brien & Wibmer (1982) contained seven species, whereas the monotypic *Piscatopus* sec. O’Brien & Wibmer (1982) was comprised solely of *Piscatopus griseus* Sleeper, 1960 sec. O’Brien & Wibmer (1982). We thoroughly redescribe these recognized species-level entities and furthermore describe ten species as new to science: *Minyomerus bulbifrons* sec. Jansen & Franz (2015) (henceforth: [JF2015]), **sp. n.**, *Minyomerus aeriballux* [JF2015], **sp. n.**, *Minyomerus cracens* [JF2015], **sp. n.**, *Minyomerus gravivultus* [JF2015], **sp. n.**, *Minyomerus imberbus* [JF2015], **sp. n.**, *Minyomerus reburrus* [JF2015], **sp. n.**, *Minyomerus politus* [JF2015], **sp. n.**, *Minyomerus puticulatus* [JF2015], **sp. n.**, *Minyomerus rutellirostris* [JF2015], **sp. n.**, and *Minyomerus trisetosus* [JF2015], **sp. n.** A cladistic analysis using 46 morphological characters of 22 terminal taxa (5/17 outgroup/ingroup) yielded a single most-parsimonious cladogram (L = 82, CI = 65, RI = 82). The analysis strongly supports the monophyly of *Minyomerus* [JF2015] with eight unreversed synapomorphies, and places *Piscatopus griseus* sec. O’Brien & Wibmer (1982) within the genus as sister to *Minyomerus rutellirostris* [JF2015]. Accordingly, *Piscatopus* sec. Sleeper (1960), **syn. n.** is changed to junior synonymy of *Minyomerus* [JF2015], and its sole member *Piscatopus griseus* sec. Sleeper (1960) is moved to *Minyomerus* [JF2015] as *Minyomerus griseus* [JF2015], **comb. n.** In addition, the formerly designated type *Minyomerus innocuus* Horn, 1876 sec. Pierce (1913), **syn. n.** is changed to junior synonymy of *Minyomerus microps* (Say, 1831) [JF2015] which has priority. The genus is widespread throughout western North America, ranging from Canada to Mexico and Baja California. Apparent patterns of interspecific diversity of exterior and genitalic morphology, varying host plant ranges, overlapping and widely extending species distributions, suggest an early origin for *Minyomerus* [JF2015], with a diversification that likely followed the development of North American desert biomes. Three species in the genus – i.e., *Minyomerus languidus* Horn, 1876 [JF2015], *Minyomerus microps* [JF2015], and *Minyomerus trisetosus* [JF2015] – are putatively considered parthenogenetic.

## Introduction

This phylogenetic revision, in compliance with ICZN (1999), re-/utilizes sets of taxonomic names to not just refer to their individual respective types (Witteveen 2015), but to variously denote previous and current taxonomic concepts (Franz et al. 2008). In order to individuate these multiple usages, we adopt the taxonomic concept approach as specified in Franz and Cardona-Duque (2013), where:

1. *Taxonomic concept labels* (name sec. author [year]; Berendsohn 1995) are used whenever we identify one specific usage of the taxonomic name. This convention is also used (where appropriate) to represent nomenclatural (type) relationships. Example: *Minyomerus* Horn, 1876 sec. Jansen & Franz (2015).
2. Solely the taxonomic name (without the sec. annotation) is used to refer to the cumulative history (origin to present) of taxonomic concepts associated with that name. Example: *Minyomerus* Horn, 1876.
3. The annotation [non-focal] is added to taxonomic names whose meanings are not under scrutiny in the present context; such as names for higher-level weevil groups and associated plants (exempting common names). Example: Tanymecini Lacordaire, 1863 [non-focal].

**Figure 1.**
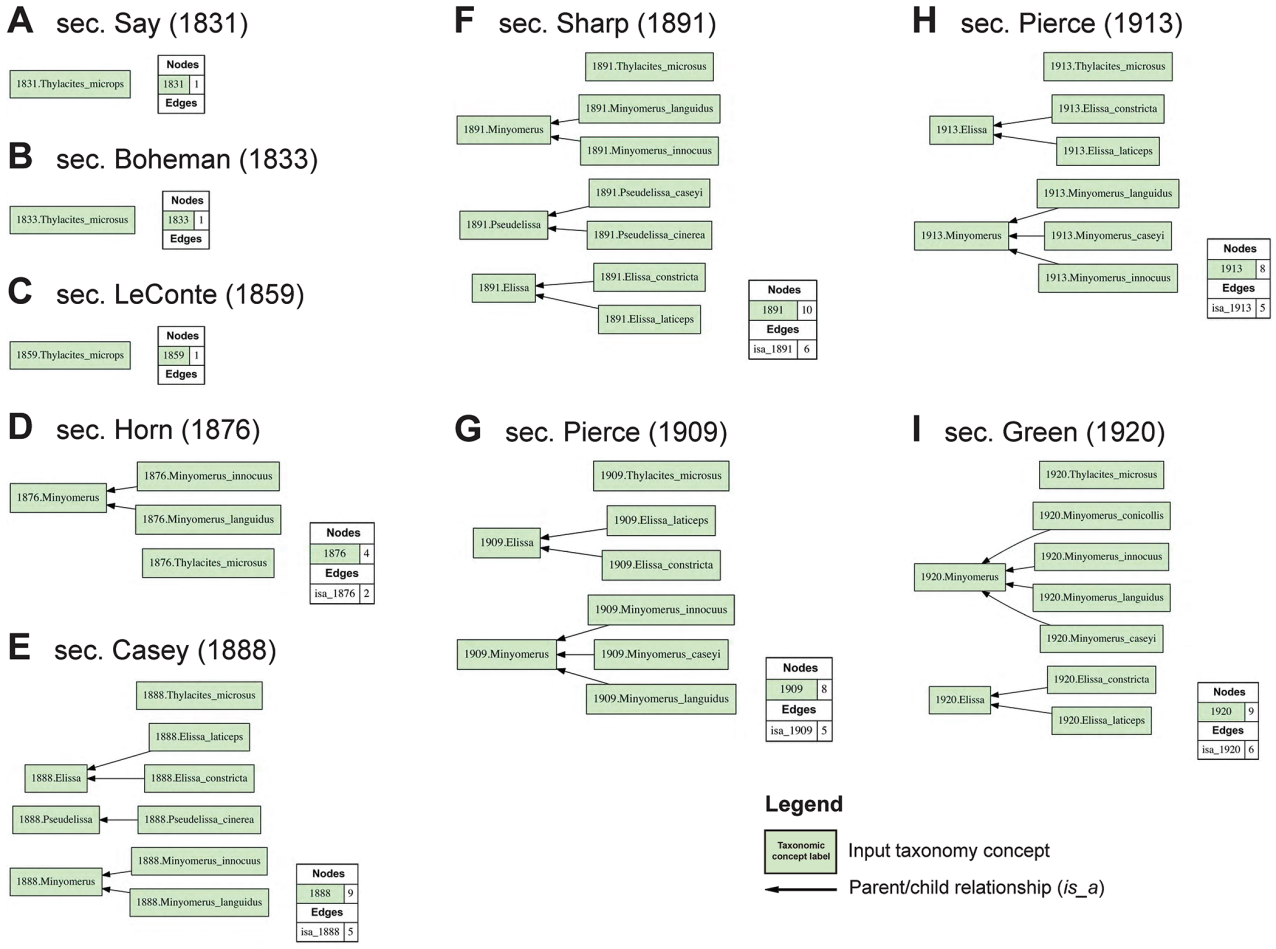
Arrangement of input visualizations 1–9 (1831–1920) representing relevant prior Taxonomies (concepts and parent/child *is_a* relationships) for the *Minyomerus* [JF2015] revision, generated with the Euler/× toolkit for concept taxonomy alignment. Annotation and representation conventions are in accordance with Franz et al. (2015a, 2015b). In particular, taxonomic concept labels such as *Thylacites microps* sec. Say (1831) are abbreviated as “1831.Thylacites_microps”. The succeeding taxonomies are represented cumulatively, i.e., all concepts explicitly and implicitly endorsed at the time of publication are represented. For further detail see Suppl. material 1. **A** sec. Say (1831) **B** sec. Boheman (1833) **C** sec. LeConte (1859) **D** sec. Horn (1876) **E** sec. Casey (1888) **F** sec. Sharp (1891) **G** sec. Pierce (1909) **H** sec. Pierce (1913) **I** sec. Green (1920).

**Figure 2.**
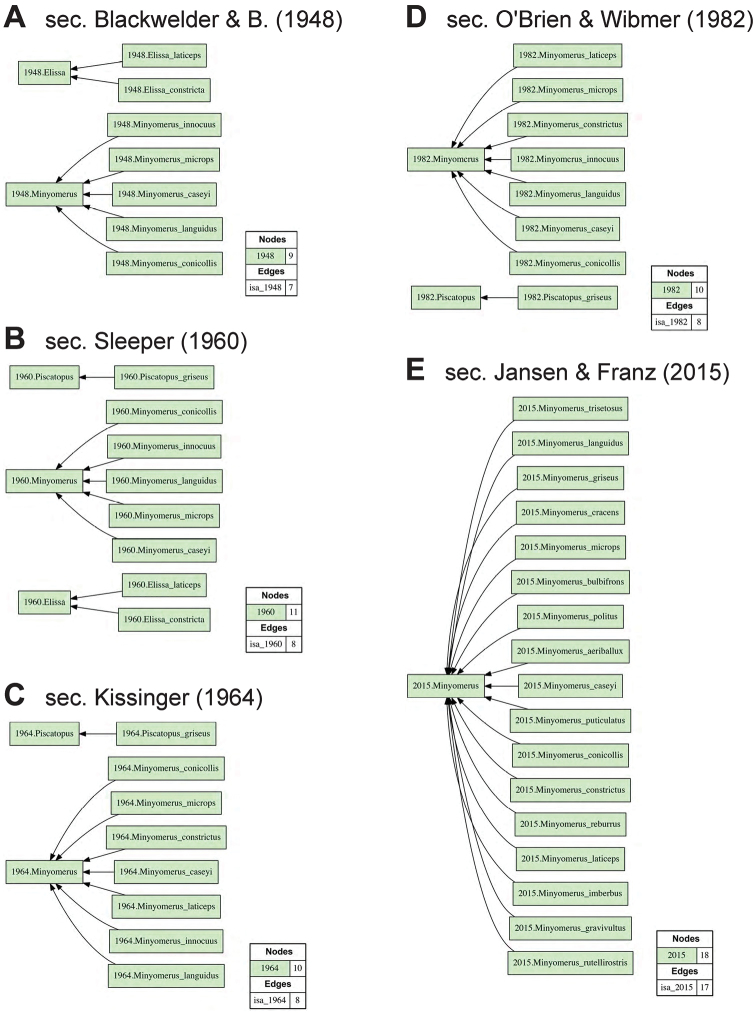
Arrangement of input visualizations 10–14 (1948–2015) representing relevant prior taxonomies for the *Minyomerus* [JF2015] revision. See Fig. 1 and Suppl. material 1 for further explanation. **A** sec. Blackwelder & Blackwelder (1948) **B** sec. Sleeper (1960) **C** sec. Kissinger (1964) **D** sec. O’Brien & Horn (1876) **E** sec. Jansen & Franz (2015).

**Figure 3.**
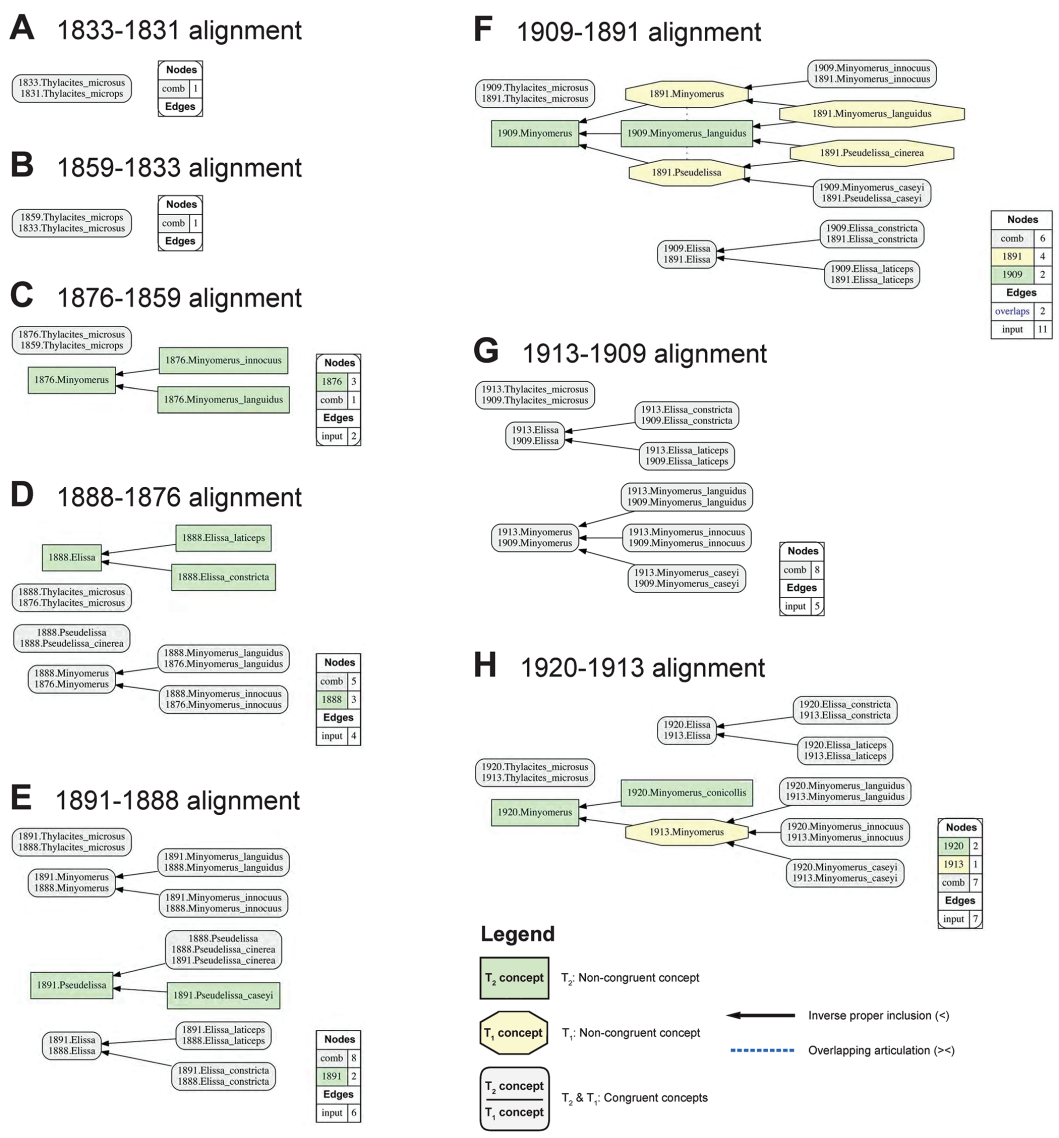
Arrangement of eight pairwise, logically consistent alignments for directly succeeding input taxonomies (1831–1920) with relevance to the *Minyomerus* [JF2015] revision, as shown in Fig. 1. The alignments and visualizations were produced using the Euler/X toolkit for concept Taxonomy, as detailed in Franz et al. (2015a, 2015b). For each pairwise alignment, congruent concept regions (originating in T_2_ and/or T_1_) are shown as grey rectangles, concepts regions unique to the later taxonomy (T_2_) are shown as green rectangles, and concept regions unique to the earlier taxonomy (T_1_) are shown as yellow octagons. Articulations of inverse proper inclusion (>) and overlap (>>) are also shown. For further detail see Suppl. material 2. **A** alignment of Boheman (1833) and Say (1831) **B** alignment of LeConte (1859) and Boheman (1833) **C** alignment of Horn (1876) and LeConte (1859) **D** alignment of Casey (1888) and Horn (1876) **E** alignment of Sharp (1891) and Casey (1888) **F** alignment of Pierce (1909) and Sharp (1891) **G** alignment of Pierce (1913) and Pierce (1909) **H** alignment of Boheman (1920) and Say (1913).

**Figure 4.**
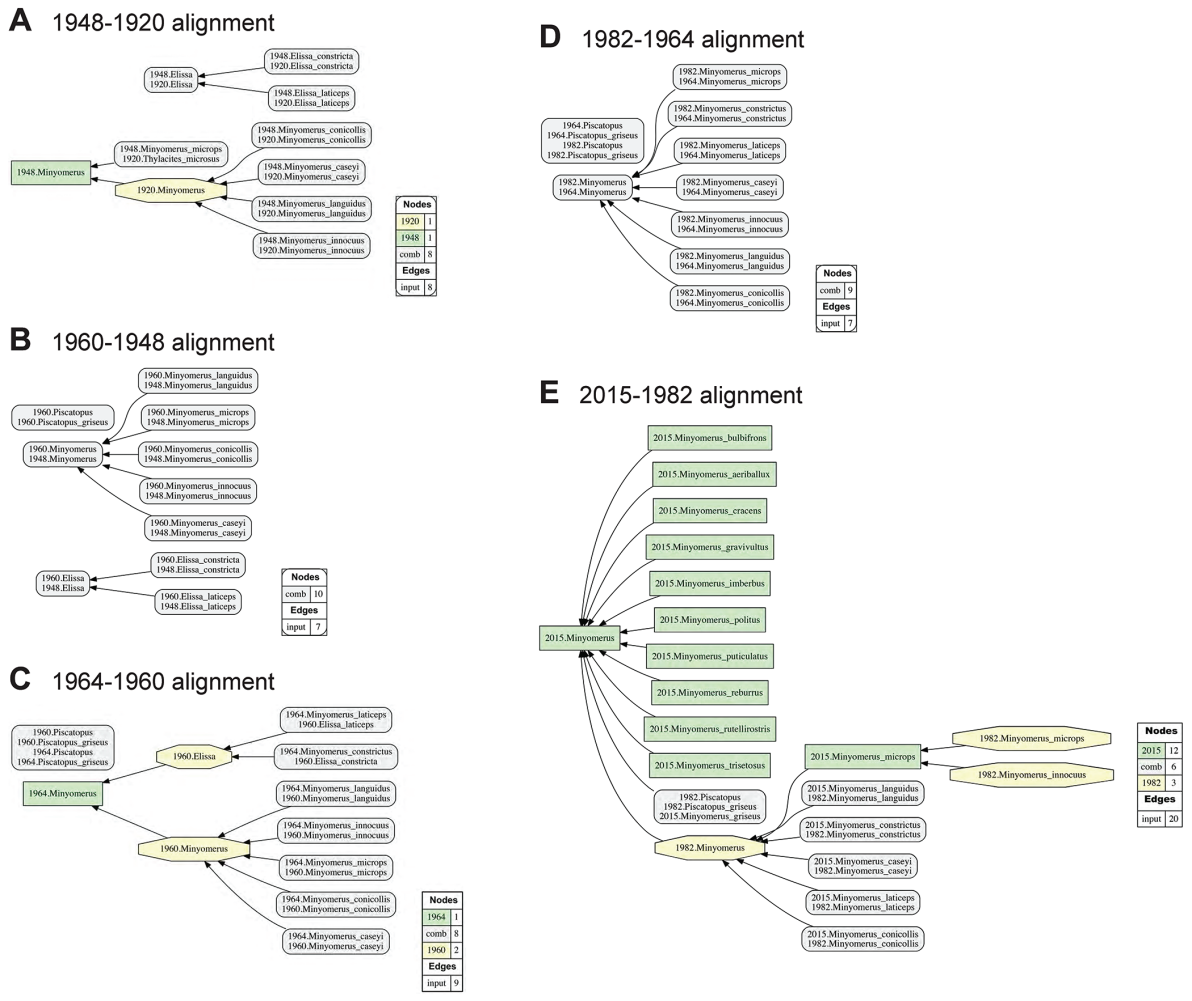
Arrangement of five pairwise, logically consistent alignments for directly succeeding input taxonomies (1920–2015) with relevance to the *Minyomerus* [JF2015] revision, as shown in Fig. 2. See Fig. 1 and Suppl. material 2 for further explanation. **A** alignment of Blackwelder and Blackwelder (1948) and Green (1920) **B** alignment of Sleeper (1960) and Blackwelder and Blackwelder (1948) **C** alignment of Kissinger (1964) and Sleeper (1960) **D** alignment of O’Brien and Wibmer (1982) and Kissinger (1964) **E** alignment of Jansen and Franz (2015) and O’Brien and Wibmer (1982).

For ease of legibility, we abbreviate the often appearing name usage specifier “sec. Jansen & Franz (2015)” with [JF2015]. This approach allows us to differentiate 14 previous and current taxonomic perspectives authored in 1831–2015 (Figs 1, 2), and to produce 13 logically inferred, pairwise alignments among the respective sets of taxonomic concepts that are directly interpretable by machines (Figs 3, 4). We acknowledge that these conventions may be unfamiliar to some readers. However, because this contribution is of an empirical nature, we defer to other publications that explain the benefits and practice of using the taxonomic concept approach (Franz and Peet 2009; Franz and Cardona-Duque 2013; Franz et al. 2015a, 2015b).

The weevil genus *Minyomerus* Horn, 1876 [JF2015], is assigned to the tribe Tanymecini Lacordaire, 1863 [non-focal], subtribe Tanymecina Lacordaire, 1863 [non-focal] (Curculionidae [non-focal]: Entiminae [non-focal] – higher-level classification in accordance with Alonso-Zarazaga and Lyal 1999 and Bouchard et al. 2011). Member species of the genus are primarily distributed throughout the desert and grassland regions of southwestern North America, ranging as far north as Alberta, Canada, and as far south as San Luis Potosí, Mexico. Adult specimens of *Minyomerus* [JF2015] have a short, broad rostrum and dehiscent mandibular process and therefore belong to the broad-nosed weevils, subfamily Entiminae [non-focal] (Marvaldi 1997; Anderson 2002; Oberprieler et al. 2007; Franz 2012; Marvaldi et al. 2014). They are covered in appressed, circular scales, with rows of sub-recumbent to erect, interspersed setiform scales (‘setae’), and can range in length from 2.8 to 6.0 mm. The genus shares with other taxa assigned to the tribe Tanymecini [non-focal] the presence of post-ocular vibrissae that project from the anterior prothoracic margin (Howden 1959, 1970, 1982). Members of *Minyomerus* [JF2015] have traditionally been differentiated from other genera of tanymecines [non-focal] based on the following traits: rounded elytral humeri; apparently contiguous procoxae; presence of stout, spiniform, ventral setae on all tarsi; profemora that are not dilated and lack spines; and the distinct scrobe channel that is directed ventrad of the eye (van Emden 1944; Kissinger 1964; Anderson 2002).

Prior taxonomic treatments of *Minyomerus* and close relatives have failed to fully recognize the North American species-level diversity, and are thus inadequate for separating many of the entailed species. Critical morphological character systems such as mouthparts or genitalia were not examined. The classificatory history also contains numerous synonymies. Lastly, the life history of species of *Minyomerus* [JF2015] was poorly known, and some authors speculated that the genus was “subaquatic” even though species exclusively occur in arid environments (Horn 1876, Casey 1888).

The relatively complicated nomenclatural and taxonomic history of *Minyomerus* is herein recognized to entail 14 relevant stages (Figs 1, 2). The history begins with the description of *Thylacites microps* sec. Say (1831) by Say (1831: 9) (Fig. 1A). Two years later the type and taxonomic entity were redescribed by Boheman (in Schoenherr 1833: 523) – who was evidently not aware of Say’s preceding treatment – as *Thylacites microsus* sec. Boheman (1833) (Fig. 1B). LeConte (1859: 268) recognized the homotypic synonymy and thus also established the priority of *Thylacites microps* sec. LeConte (1859) (Fig. 1C). However, the latter, priority-carrying name has not been in regular use from that time until the present revision.

The genus-level name and concept *Minyomerus* sec. Horn (1876) were originally created by Horn (in LeConte and Horn 1876: 17) with two constituent species (concepts): *Minyomerus innocuus* Horn, 1876 sec. Horn (1876), and *Minyomerus languidus* Horn, 1876 sec. Horn (1876) (Fig. 1D). The former name (individuated as *Minyomerus innocuus* sec. Pierce [1913]) was subsequently designated as the type of *Minyomerus* sec. Pierce (1913) by Pierce (1913: 400) (Fig. 1H). Two junior synonyms of *Minyomerus* [JF2015] – viz. *Elissa* Casey, 1888 sec. Kissinger (1964), and *Pseudelissa* Casey, 1888 sec. Pierce (1909) – were changed to their current status by Kissinger (1964: 30; Fig. 2C) and Pierce (1909: 359; Fig. 1G), respectively. *Elissa* sec. Casey (1888) originally (Casey 1888: 271–272) entailed two species (concepts): the type species, *Elissa laticeps* Casey, 1888 sec. Casey (1888), and *Elissa constricta* Casey, 1888 sec. Casey (1888) (Fig. 1E). *Pseudelissa* sec. Casey (1888), described in Casey (1888: 273-274), was initially comprised of one species (concept): *Pseudelissa cinerea* Casey, 1888 sec. Casey (1888) (Fig. 1E), which was later changed to junior synonymy of *Minyomerus languidus* sec. Pierce (1909) by Pierce (1909: 359) (Fig. 1G). *Pseudelissa* sec. Sharp (1891) had previously added the species-level member *Pseudelissa caseyi* Sharp, 1891 sec. Sharp (1891) (Fig. 1F). With the addition of *Minyomerus conicollis* Green, 1920 sec. Green (1920) (Fig. 1I), and the transfer of *Thylacites microps* to *Minyomerus* sec. Blackwelder & Blackwelder (1948: 46; Fig. 2A), the genus-level concept *Minyomerus* sec. O’Brien & Wibmer (1982) had expanded to contain seven species (concepts) as recognized in O’Brien and Wibmer (1982: 46; Fig. 2D). Meanwhile Sleeper (1960: 84-86; Fig. 2B) described *Piscatopus* Sleeper, 1960 sec. Sleeper (1960), and its sole member and type *Piscatopus griseus* Sleeper, 1960 sec. Sleeper (1960), as closely related to but distinguished from *Elissa* sec. Sleeper (1960) (synonymized in Kissinger 1964 with *Minyomerus* sec. Kissinger [1964]; Fig. 2C) by “having the front coxae separated by the prosternum” and several other traits.

Prior and newly conducted field and museum research efforts have made available extensive specimen material, encompassing ten new species of *Minyomerus* [JF2015] which are herein described and integrated into a comprehensive phylogenetic revision of this group of North American weevils (Fig. 2E). Many existing type specimens were available for study through either loans or shared, well-resolved images, including types of taxa newly placed into synonymy. Based on this material we provide detailed morphological re-/descriptions of all previously recognized and new taxa, a phylogenetic analysis using morphological evidence pertaining to 22 terminals (5 outgroup, 17 ingroup), and an amended classification and identification key consistent with these insights. Our revision has implications for the status of *Piscatopus* [JF2015], which is phylogenetically entailed within *Minyomerus* [JF2015] in the sense of the present treatment. The validity of *Minyomerus innocuus* [JF2015] and *Minyomerus microps* [JF2015] as names of the types of *Minyomerus* [JF2015] is also resolved. We furthermore provide new information on species distributions, historical biogeographic relationships, habitat preferences, life history traits, and host plant records for members of *Minyomerus* [JF2015], as well as some indications concerning their reproductive strategies and the timing of their evolutionary radiation.

## Material and methods

**Natural history observations.** Members of the genus *Minyomerus* [JF2015] are typically found on a variety of host plants, as indicated following each species account. Many species are likely generalists with regards to their host plant spectrum. Adult weevils are most commonly observed on the leaves and branches of the host where they feed on the leaf tissue. Eggs, larvae, and pupae remain unknown. The larvae are probably root feeders, as observed in the tanymecine [non-focal] genus *Tanymecus* Germar, 1817 [non-focal] (Howden 1975). They typically inhabit arid regions, especially deserts, throughout southwestern North America, including the Mojave, Sonoran, Chihuahuan, and Great Basin Deserts. They also occur in semi-arid regions of the Great Plains, on the Colorado Plateau, and throughout Baja California, Mexico. The adults apparently disperse without flight, as the hind wings and associated flight structures of all species in the genus are greatly reduced and not readily apparent after dissections. In several species only females are known, and some are putatively considered parthenogenetic, as indicated in the respective descriptions.

**Field and museum work.** Extensive field and museum work was undertaken in support of this revision during the period of May 14, 2013 to July 02, 2013. More than 8000 miles were covered to access and sample known and expected habitats for *Minyomerus* [JF2015] species in Arizona, California, Colorado, Nevada, New Mexico, Texas, and Utah. Sampling protocols included collection by sweeping, beating putative host plants, and mercury and ultraviolet light trapping. Specimens were preserved in 95% ethanol and kept cool for the duration of the trip. Detailed field notes included the precise GPS location, habitat type, soil consistency, and, where apparent, host plant associations. Georeferencing of localities was performed with Google Earth (Google Inc. 2009–2015) and reported in decimal degrees. Taxonomic names for associated host plants are used in accordance with Munz and Keck (1973) and SEINet (2015).

The set of newly collected *Minyomerus* [JF2015] specimens was supplemented with abundant material housed in the following collections, using the codens of Arnett et al. (1993):

ASUHIC Hasbrouck Insect Collection, Arizona State University, Tempe, Arizona

BYU M.L. Bean Life Science Museum, Brigham Young University, Provo, Utah

CAS California Academy of Sciences, San Francisco, California

CMNC Canadian Museum of Nature Collection, Ottawa, Canada

CSCA California State Collection of Arthropods, Sacramento, California

CWOB Charles W. O’Brien Collection, Green Valley, Arizona

EMEC Essig Museum of Entomology, University of California, Berkeley, California

NMSU New Mexico State University, Las Cruces, New Mexico

NVDA Nevada State Department of Agriculture, Reno, Nevada

TAMU Texas A & M University, College Station, Texas

TTUZ Texas Tech University, Lubbock, Texas

UCDC Bohart Museum of Entomology, University of California, Davis, California

UMNH Utah Museum of Natural History, University of Utah, Salt Lake City, Utah

UNMC University of New Mexico Albuquerque, New Mexico

USNM National Museum of Natural history, Washington, D.C.

Labels for newly designated type specimens include the genus name and species epithet, gender symbol (♀/♂), and the author names and years. They are colored red for holotypes and yellow for paratypes. Identification labels for non-type specimens examined in this revision are white.

**Morphological analysis.** The herein utilized systematic and descriptive approach follows Franz (2010a, 2010b, 2012). Terminology for exterior morphology is in general accordance with Torre-Bueno (Nichols 1989). Additional morphological terms specific to (entimine) weevils were used as follows: Ting (1936) and Morimoto and Kojima (2003) for mouthparts; Thompson (1992) for tibial apices and abdominal segments; and Oberprieler et al. (2014) and Howden (1995) for male and female terminalia.

All measurements were taken using a Leica M205 C stereomicroscope and attached computer running the software Leica Application Suite (LAS), version 4.1.0. The overall body length and width were measured in dorsal view as the maximum distance between the apex of the rostrum and the apex of the elytra, and the maximum width of the elytra, respectively. The length of the rostrum was measured in dorsal view as the distance extending from the apex of the epistoma to the anterior margin of the eyes. The width of the rostrum was measured in dorsal view as the maximum distance between the dorsal margins of the rostrum near the point of antennal insertion. The length of the pronotum was measured in dorsal view as the length along the midline from the anterior margin to the posterior margin. The width of the elytra was measured in dorsal view as the maximum distance between the lateral margin and the elytral suture. Other length and width measurements were performed in dorsal orientation, as the maximum length and width of the corresponding structure (profemur, protibia, elytron, and aedeagus). Images of mouthparts and terminalia were produced with the Leica microscope equipment, whereas habitus photographs were created with a Visionary Digital Passport II sytem using a Canon EOS Mark 5D II camera.

Species of *Minyomerus* [JF2015] recognized in this revision were delimited through application of the phylogenetic species concept *sensu* Wheeler and Platnick (2000). Species descriptions follow the inferred phylogenetic sequence (see details below). The genus-level description for *Minyomerus* [JF2015] highlights characters present in all members and accounts for their apparent variability. Consequently, the species descriptions represent unique and complementary accounts of the character states observed in each species, including their variability, and excepting characters that are invariate within the genus as specified in the higher-level description of *Minyomerus* [JF2015]. Likewise, descriptions of males in the genus- and species-level accounts emphasize characters that are variable and sufficiently different from those of the females to merit recognition. Some redundancy occurs between genus and species descriptions when discussing characters that vary widely within single species, and also between species descriptions where a character exhibits considerable variation in some species yet is invariant in others.

The key to identifying species of *Minyomerus* [JF2015] is arranged with emphasis being placed on the most readily observable diagnostic characters. The revision is arranged with the species descriptions appearing first, followed by the key to species, and then by the phylogenetic results.

**Phylogenetic analysis.** The morphological cladistic analysis includes 22 terminal taxa; with 17 ingroup and 5 outgroup terminals. Following resolution of species identities and boundaries, the ingroup terminals were represented by six species previously assigned to *Minyomerus* sec. O’Brien & Wibmer (1982), *Piscatopus griseus* sec. Sleeper (1960), and ten newly recognized species. The lack of prior phylogenetic analyses of *Minyomerus* and closely related lineages made it necessary to sample outgroups fairly broadly while remaining focused on North American lineages that are are putative close relatives of the ingroup (Nixon and Carpenter 1993). The tribe Tanymecini [non-focal] is cosmopolitan, with the subtribe Tanymecina [non-focal] containing the majority of New World species diversity in the tribe (Alonzo-Zarazaga and Lyal 1999). Accordingly, four outgroup terminals are represented by species belonging to separate genera in the Tanymecina [non-focal]; viz. *Pandeleteius cinereus* (Horn, 1876) [non-focal], *Pandeleteinus subcancer* Howden, 1969 [non-focal], *Isodrusus debilis* Sharp, 1911 [non-focal], and *Isodacrys buchanani* Howden, 1961 [non-focal]. Because generic relationships in the Tanymecini [non-focal] remain unresolved, it was deemed prudent to find a relatively far removed taxon to root the cladogram that would nevertheless display states that are applicable to the ingroup for all characters under consideration (Rieppel 2007; Franz 2014). For this purpose we select the North American species *Sitona californicus* (Fahraeus, 1840) [non-focal], representing the tribe Sitonini Gistel, 1856 [non-focal].

The character matrix was edited and phylogenetic results reviewed and reconnected to the input using the WinDada and WinClados interfaces of WinClada (Nixon 2002). The character sequence follows that of the taxonomic descriptions. The most parsimonious tree and character state optimizations were inferred under parsimony using NONA (Goloboff 1999). An unconstrained heuristic search of tree space for the 22-terminal matrix was conducted using the commands: hold 100001, mult*1000, hold/100, with mult*max* selected. Bootstrap support was inferred in WinClada using the parameters of 1000 replications, hold 1000, hold/100, mult*10, “Don’t do max*”, and “Save consensus”. Finally, Bremer support values (Bremer 1994) were calculated in NONA using the commands hold 20000, suboptimal 20, and bsupport 20.

**Nomenclatural and taxonomic provenance.** In accordance with Franz and Peet (2009), we use the symbol “=” to indicate nomenclatural synonymy (homo-/heterotypic); and “==” (congruence) and “>” (proper inclusion) to indicate taxonomic concept articulations. The annotations (INT) and (OST) indicate intensional and ostensive readings of articulations, and AND is used to connect multiple simultaneously recognized provenance relationships. The corresponding taxonomic provenance analysis input/output files (Figs 1–4) have been placed in the Dryad Digital Reposistory (see Suppl. material 1). For further information on taxonomic provenance methods see Franz and Cardona-Duque (2013) and Franz et al. (2015a).

**Species habitat modeling.** We used the modeling program Maxent, Version 3.3, to generate habitat models for the species of *Minyomerus* [JF 2015] (Figs 50–52) based on the documented occurrence records (Phillips et al. 2004, 2006; Elith et al. 2011). The default settings “Max number background points”, set to 100,000, and “Iterations” set to 10 and with cross-validation, were applied to leverage all available locality data. However, no models could be created for three species with single documented localities; and only a single-iteration habitat model could be generated for a fourth species due to limited information data.

We selected 19 bioclimatic variables and elevation as Environmental Layers in Maxent, obtained from WorldClim (see Hijmans et al. 2005 and the associated website for further detail on variables). The layers were downloaded by tile (zones 11–13 and 21–23), with a 30 arc-second resolution (projected using WSG 84) to provide adequate coverage of the full distribution of *Minyomerus* [JF2015]. These tiles were assembled, by layer, into composite maps of six tiles each, using QGIS, Version 2.8.1 ‘Wien’, prior to species distribution modeling (Quantum GIS Development Team 2015).

After modeling, the raster files of the predictive probabilities were imported into QGIS. Each file was designated a specific color, and each pixel in the raster grid was assigned a linearly interpolated saturation of that color, with increasing saturation denoting an increased probability of successful prediction of species presence at that point. Pixels with a value below 0.50 were rendered transparent so that the model only shows regions with a greater than 50% chance of successful prediction. The raster files were clipped to remove extraneous predicted regions based on: (1) predictive probability (i.e., removing large areas with only transparent pixels) and (2) geographic extent (accounting for endemicity). For instance, species endemic to Baja California Sur, Mexico, do not require a predictive model for bioclimatically similar habitats in Canada. The actual (documented) occurrence records are laid over the modeled habitat ranges as colored circles on the respetiv maps (Figs 50–52), along with vector layers of country and state borders (Hijmans et al. 2012).

## Systematic revision

### Genus Minyomerus [JF2015]

Taxon classificationAnimaliaColeopteraCurculionidae

Horn, 1876 sec. Jansen & Franz (2015)

> Minyomerus Horn, 1876: 17 sec. Horn (1876)
> AND = Elissa Casey, 1888: 271 sec. Casey (1888) (synonymized by Kissinger 1964: 30)
> AND = Pseudelissa Casey, 1888: 273 sec. Casey (1888) (synonymized by Pierce 1909: 359)
> AND = Piscatopus Sleeper, 1960: 84 sec. Sleeper (1960), syn. n.

#### Type species.

*Minyomerus microps* (Say, 1831: 9) sec. Jansen & Franz (2015), stat. n.

== (INT) AND > (OST) AND = *Thylacites microps* Say, 1831: 9 sec. Say (1831) (transferred to *Minyomerus* sec. Blackwelder & Blackwelder [1948] on the authority of Buchanan *in litt*. by Blackwelder and Blackwelder 1948: 46)

== (INT) AND > (OST) AND = *Thylacites microsus* Boheman, 1833: 523 sec. Boheman (1833) (synonymized by LeConte 1859: 286)

== (INT) AND > (OST) AND = *Minyomerus innocuus* Horn, 1876: 18 sec. Horn (1876) (type, designated by Pierce 1913: 400), syn. n.

#### Diagnosis.

*Minyomerus* [JF2015] is diagnosed by a unique combination of synapomorphic traits; specifically, the integument is covered by appressed scales that are sub-circular and overlap posteriorly; the nasal plate is present as a broad, scale-covered, chevron-shaped ridge demarcating the epistoma; a sulcus posteriad of nasal plate is present; the scrobe is subequal in length to the funicle and club combined; the head is directed slightly ventrally; the metatibial apex lacks setiform bristles yet displays bristles that are shorter to subequal in length to the surrounding setae and conical to lamelliform; the mesotarsi are slightly shorter than the mesotibiae, and all tarsi lack pads of setiform setae but have stout, spiniform setae. The following additional characters are useful for identifying members of *Minyomerus* [JF2015], especially when differentiating the former from other genera of Tanymecini [non-focal] such as *Isodrusus* Sharp, 1911 [non-focal], *Isodacrys* Sharp, 1911 [non-focal], and *Pandeleteinus* Champion, 1911 [non-focal] (see also Anderson 2002): the intercoxal process of the prosternum is medially divided into two halves (with the procoxae apparently contiguous in most); the elytral humeri are rounded rather than angled and protruding; the profemora are not dilated and lack spines; the protibiae are ventrally excavated by a longitudinal groove or concavity; and a distinct scrobe is present and directed ventrad of the eye, with a more or less apparent tooth formed by an overhang of the dorsal margin.

#### Description – female.

*Habitus*. Length 2.80–6.49 mm, width 1.02–2.41 mm, shape elongate and sub-cylindrical, length/width ratio 2.18–2.94 mm, widest at anterior 1/5–2/5 of elytra. Integument tan to black, vestiture consisting of appressed sub-circular to occasionally irregular squamiform scales, arranged densely throughout, partially overlapping; color and opacity varying among species, from white to dark brown, though generally brownish, in some species appearing semi-translucent (in others opaque), metallic, or opalescent; with interspersed colors forming small maculae, bands and other variously scattered patterns; scales generally becoming lighter ventrally, including rows of setae. Linear setiform scales (‘setae’) sparse throughout, short, sub-erect to sub-recumbent, brown to white, arranged in rows on elytral intervals, and becoming longer on humeri and venter.

**Mouthparts.** *Mandibles.* Covered with non-overlapping, sub-circular to sub-quadrate, whitish or opalescent scales, with several setae; ovate scar from deciduous process located apicolaterally.

*Maxillae* (Fig. 10B). Cardo as long as distance from base of palpomere I to base of palpiger, wider than palpomere III, bifurcate at base with an inner angle typically between 90–120°, arms of variable length, inner (mesal) arm thicker than outer arm in most species, apical end strongly curved outward (laterally) at a 90° angle, one or both arms of bifurcation equal in length to apically outcurved arm, glabrous. Stipes short, sub-quadrate to sub-rectangular, roughly equal in length to one or both bifurcations of cardo, glabrous or with a single lateral seta. Galeo-lacinial complex not extending to apex of maxillary palpomere I, apically rounded; complex membranous and setose in posterior 1/2-3/4, sclerotized and somewhat emarginate anteriorly; dorsally with 5-9 apicomesal lacinial teeth; ventrally with 1-5 reduced lacinial teeth. Palpiger with a transverse row or patch of setae; anterior portion variably membranous, posteriorly sclerotized. Maxillary palps three-segmented; I longer than II, I apically oblique, apical end facing mesally and forming 30–60° angle with base, I and II with variably inserted setae; II shorter than III; III elongate, with parallel sulci and apical sensilla.

*Labium* (Fig. 10A). Prementum completely covering maxillary palps; often pentagonal or hexagonal, ventrally sub-planar, concave laterally; margins of prementum with variable degrees of curvature, apicomedially projected (ligula), ligula angulate; each lateral region with 1 long seta. Labial palps 2- or 3-segmented, variably exposed; II shorter than I, both usually with 1 apical seta; III similar in length to II, when present; III apically constricted and with sensilla.

**Rostrum.** Length 0.38–0.83 mm, appearing markedly reduced in length, anterior portion variably broader than long, sub-equal in width to head, rostrum/pronotum length ratio 0.41–0.75, rostrum length/width ratio 0.73–1.43; shape in cross section sub-rectangular for most species. Separation of rostrum from head generally obscure; rostrum sub-divided into a short, planar, transverse, anterior section, and a larger, convex, posterior section with a seamless transition into rest of head. Dorsal outline of rostrum square to trapezoidal, anterior half of dorsal surface mesally planar, posterior half convex and rugoso-punctate. Rostrum in lateral view nearly elongate-rectangular to square; basal half of dorsolateral margins converging anteriorly, anterior half sub-parallel; apical margin emarginate and bisinuate, with 2–6 large vibrissae, each inserted laterad of each sinuation. In frontal view, nasal plate defined by V-shaped or Y-shaped, impressed lines, concave to convex, integument covered with non-overlapping sub-circular white or opalescent scales, and with interspersed apically directed setae. Margins of mandibular incision straight, slightly diverging dorsally in frontal view, bounded by same type of scales as those on nasal plate. Ventrolateral sulci variably defined as a deep notch or sulcus dorsad of insertion point of mandibles, running parallel to the scrobe. Dorsal surface of rostrum with median fovea at posterior end of nasal plate; ventrolateral margins sub-parallel. Rostrum ventrally with a median fovea and 2 sub-parallel sulci beginning at corners of oral cavity and continuing as small foveae towards base of rostrum; with 2 foveae laterad of former and roughly in line with insertion point of mandibles; these sulci and foveae can be variably expressed. Oral cavity with lateral margins nearly straight (Fig. 6A).

**Antennae.** Antennal insertion near apical 1/3 of rostrum, dorsal to posterior margin of mandibular insertion point. Scrobe lateral, strongly curved, with parallel edges nearly continuing to anterior margin of eye; dorsal margin of scrobe overhanging slightly and forming a minute tooth, variably located relative to eye. Antennae apparently 12-segmented, segment 11 with annulus that lacks an inner phragm. Scape slender, clavate; directed ventrad of eye in idealized position; covered with appressed, squamiform scales with interspersed setae on clubbed section of scape. Funicle 7-segmented; sub-equal in length to scape; funicular antennomeres progressing from elongate to equilateral, clavate, covered with appressed scales and apically directed, interspersed setae; segments becoming less clavate and shorter with increasing proximity to club, except for terminal segment, which is longer and wider than preceding segment; where noted, some species without scales on terminal segment, clothed as antennal club. Club appearing 4-segmented, terminal segment with annulus that lacks an inner phragm; similar in length to funicular antennomeres III-VII, 2.5–3.0 × as long as wide, with a covering of apically-directed pubescence with interspersed sub-erect setae.

**Head.** Eyes small, laterally positioned, globular, coarsely facetted, protruding, anterodorsal margin of each eye impressed, posterior margin elevated from lateral surface of head; eyes separated in dorsal view by 3–6 × their anterior-posterior length, set off from anterior prothoracic margin by up to 1/2 of their anterior-posterior length. Head between eyes rugose and bulging. Head typically with a broad, transverse post-ocular impression.

**Thorax.** *Pronotum.* Variously equilateral (with dimensions of dorsal, lateral, and ventral surfaces equal, or nearly so), length/width ratio 0.68–1.06, surface transversely convex, sub-cylindrical; widest near midpoint; shape varying slightly from typical form in some species; surface punctate, punctures often obscured by scales; median sulcus present, sometimes not visible, beginning just beyond anterior constriction continuing to just anteriad of posterior margin. Anterior margin ranging from straight and even to slightly curved and somewhat produced dorsally, lateral margins evenly curved and widening into a bulge near midpoint, anteriorly constricted (sometimes subtly so); posterior margin straight to incurved. Pronotum in lateral view sub-cylindrical, narrower ventrally, with transverse ventrolateral sulci running sub-parallel to anterior and posterior margins, respectively; sometimes with scales forming a whitish stripe that continues along each elytron; with evenly spaced, anteriorly directed, sub-recumbent setae variously inserted near anterior margin; antero- and posterolateral margins with a fringe of appressed scales, with plumose setae beneath. Anterolateral margin with a full or reduced tuft of post-ocular vibrissae present, emerging near eye; vibrissae achieving a maximum length up to anterior-posterior length of eye.

*Scutellum.* Usually exposed, covered with appressed scales, triangular, equilateral, lateral margins slightly incurved.

*Pleurites.* Mesepisternum sub-triangular; mesepimeron trapezoidal, longer anterior edge meeting posterior side of mesepisternum; metepisternum linear, anteriorly abruptly widening into a triangular shape, gradually thinning posteriorly and covered by elytron near posterior portion of metasternum; metepimeron entirely covered by elytron.

*Sterna.* Prosternum longer than mesosternum; procoxal cavities positioned at midpoint, appearing contiguous, prosternal process usually not complete between coxae, slightly elevated. Mesosternum shorter than metasternum; anterior 1/2 incompletely covered by plumose scales, posterior portion as remainder of body surface; mesocoxal cavities separated by distance 1/6–2/5× width of mesocoxal cavity. Metasternum with a more or less obscure transverse sulcus posteriad of anterior that continues to lateral extent of coxae; metacoxal cavities separated by 1.5× their width. Metendosternite strongly reduced.

***Legs.*** Prothoracic legs longer than mesothoracic legs; scale colors variously interspersed, setation generally similar to that of remainder of body surface; tibiae ventrally with rows of longer sub-erect setae, tibiae and trochanters of all legs with a single, hair-like, brown seta positioned on mesal surface, approximately 3× length of adjacent setae. Profemur/pronotum length ratio 0.08–1.15; profemur moderately stout, slightly incurved, in cross section elliptical; proximal 4/5 of profemur gradually widening, then abruptly constricted with distal 1/5 produced ventrally as an obliquely rounded to semicircular projection covering tibial joint; condyle of tibial articulation occupying 4/5 of distal surface and 1/5 length of femur. Protibia/profemur length ratio 0.81–1.01; protibia typically moderately long and slender, straight, in cross section elliptical, apically expanded; protibial apex obliquely truncate, ventral setal comb situated on a flat surface, setal comb broken posteriorly, and becoming thinner and sparser anteriorly, setae also becoming shorter and more stout anteriorly; mucro present as a laterally projected tooth of variable size, triangular. Protarsus with tarsomere I nearly 2 × as long as II, elongate-conical; II and III similar in length, III wider than II; II conical, III bifid, jointly similar in length to V; IV mostly hidden by III; claw paired, separate, simple. Meso- and metathoracic legs slightly shorter and longer than prothoracic legs, respectively, all legs generally sub-equal in length with differences relatively small; mesotibiae with a pecten surrounding condylar surface, ring posteriorly interrupted; metatibial apex entirely scale covered, with strong outer bevel and inner flange (“corbel closed”; see Thompson 1992), outer bevel longer than inner flange, terminating in an oblique, almond-shaped convex ity ringed by a number of short, spiniform setae. Meso- and metatarsi similar to protarsi. All tarsi ventrally with spiniform setae.

***Elytra.*** Length/width ratio 2.58–3.54; widest at anterior 1/5–2/5; anterior margins jointly 3/4–2 × wider than posterior margin of pronotum, curved posteriorly; humeri broadly rounded, not strongly projected; lateral margins slightly converging posteriorly, becoming more strongly rounded and converging apically; posterior margins constricted and narrower ventrally, posteriorly narrowly truncated. Elytra in lateral view convex, widening slightly posteriorly; posterior declivity broadly arcuate dorsally, nearly straight thereafter, angled at 45–80° to main body axis. Elytra with 10 complete striae; striae distinctly punctate, covered with scales, sub-equal in width to intervals; stria 10 and lateral margin sinuate; strial punctures distinct, variably separated by several times their diameter; intervals elevated and impunctate; scales completely covering integument, colors variously interspersed, most species have some specimens with a white stripe laterally continuing from pronotum, these stripes more or less defined on some specimens; each interval medially with a row of sub-erect to sub-recumbent setae.

**Wings.** Apterous.

**Abdomen.** *Sterna.* Ventrites III and IV jointed, V-VII free; scales similar to elytra, though generally of a lighter color, including rows of sub-erect setae; III longer than IV, midregion planar, posterior margin somewhat emarginate mesally, elevated and set off from IV along lateral 1/4-1/3s of its length, somewhat concave anteriorly; IV medially longer than V and VI jointly, laterally sub-equal in length; V and VI similar in length, margins straight. Sternum VII mesally 1/2-1× as long as wide, sub-triangular; setae lengthening slightly and becoming darker and more erect posteriorly; anterior margin straight to broadly curved; posterior margin broadly arcuate, emarginate, and rimmed with short, posteriorly directed setae.

*Terga.* Tergum VII broadly arcuate posteriorly, somewhat convex, with interspersed setae becoming stouter and more densely arranged medio-posteriorally, posterior margin plicate and emarginate medially. Pygidium (tergum VIII) entirely covered by elytra, convex, sub-cylindrical to sub-conical (with lateral edges folded beneath), anterior edge broadly incurved mesally, posterior margin plicate, with a few minute setae inserted along rim; medial 1/3 of anterior 1/2 of pygidium less sclerotized.

**Terminalia** (Fig. 14). *Sternum VIII*. Anterior 7/8 (spiculum ventrale) narrowly stylate, anterior end slightly knobbed; posterior 1/8 (lamina) sub-quadrate, dorsally evenly concave, arms entire; anterior edges each incurved forming a slightly obtuse angle with lateral margin, produced to a point anteromedially at connection to spiculum ventrale; a less sclerotized region present anteriorly with anterior and lateral edges straight, latter sub-parallel; sclerotized region with pores laterally, more or less sclerotized medially; posterior edge plicate, with a fringe of sparse long setae.

*Ovipositor.* Coxites (distal gonocoxites) less sclerotized posteriorly, becoming more sclerotized anteriorly, 1/2–2× as broad as long in dorsal view, slightly narrower posteriorly, and with sparse long setae throughout; styli sub-equal in length to coxites (latter short), digitate, narrowed apically, attachment to each coxite somewhat oblique, with 2 long setae near base. Genital chamber slightly shorter than sternum VIII.

*Spermatheca.* Comma-shaped; collum short, apically with a large, hood-shaped projection perpendicular to ramus, length and alignment with curvature of bulb of ramus variable, some species ante-apically with a long, perpendicular, cylindrical projection; collum short, cylindrical, sub-contiguous with, and typically angled at 90° to ramus; ramus elongate, bulbous, sometimes stalked, generally equal in thickness to corpus; corpus swollen or not; cornu elongate, apically, gradually narrowed, strongly recurved along its length.

#### Description – male.

Males are generally similar to females in appearance, and in many species are difficult to separate from female specimens. Variation within males can often exceed intersexual boundaries, further complicating their identification as male specimens. The description of males is therefore limited to characters and states that are sufficiently and consistently different between the sexes.

*Habitus.* Length 2.41–5.82 mm, width 0.90–2.72 mm, length/width ratio 1.77–3.17. Rostrum length 0.38–0.78 mm, rostrum/pronotum length ratio 0.42–0.81, rostrum length/width ratio 0.71–1.55. Pronotum length/width ratio 0.72–1.06. Profemur/pronotum length ratio 0.76–1.33, protibia/profemur length ratio 0.76–1.00. Elytra length/width ratio 2.72–3.59.

*Mouthparts.* As in female, mentum slightly more angular, broader, and produced laterally, overall with slightly straighter margins.

*Elytra.* Generally narrower relative to pronotum, elytral declivity slightly more angulate with regard to main body axis, but otherwise as in female.

*Abdomen.* Sternum IV relatively shorter, mesally slightly longer and laterally shorter than V and VI jointly. Sternum VII narrower than female, sub-trapezoidal, posterior margin straight mesally. Tergum VII convex, anterior margin posteriorly incurved, medially less sclerotized anteriorly, with interspersed setae becoming stouter and more densely arranged posteriorally, posterior margin cultellate and incurved mesally. Pygidium (tergum VIII) entirely covered by elytra, convex, sub-cylindrical (with lateral edges folded beneath), anterior margin broadly incurved, posterior margin arcuate, subtly incurved mesally, plicate; posteriorly punctate, each puncture with a single seta; anteriorly rugose.

**Terminalia** (Fig. 15). *Sternum VIII*. Consisting of 2 sub-triangular sclerites; each sclerite slightly wider anteriorly than long, with lateral and mesal margins curved, mesal margins with 6–8 setae posteriorly, anterior margins sinuate, laterally produced to a point; dorsal surface with a patch of short, fine setae laterally; spiculum relictum not apparent.

*Spiculum gastrale* (sternite IX) slightly longer than pedon of aedeagus; apical 1/5 expanded to an alate lamina (basal plate) with 2 sclerotized, gradually narrowing projections whose lateral margins are parallel. These projections located on lateral 1/3 of posterior margin, as long as sub-quadrate portion of lamina. Mesal 1/3 with a short, sub-trapezoidal projection. Stylus (apodeme of sternite IX) straight along mesal 1/2, curving gently along anterior 1/4; anteriorly explanate.

*Tegmen.* Slightly longer than pedon; tegminal apodeme (manubrium) stylate, feebly sinuate, and slightly expanded anteriorly; posterior ring with 2 posteriorly directed, narrowly triangular projections (parameres).

*Aedeagus.* Pedon length/width ratio 2.83–7.82; antero-ventral margin membranous, mesally curved; lateral margins gradually, evenly converging posteriorly to an acute point; in lateral view evenly curved; width becoming gradually narrower posteriorly, ventral margins in region of ostium sinuate; dorsally with sparsely arranged, short, fine setae laterally, becoming slightly more densely arranged meso-posteriorly; apex angulate. Ostium elongate-ovate, laterally emarginate, basal and apical edges each with a recurved invagination. Internal sac variously plicate, membranous except for 2 sclerotized, sinuate-uncinate rami; gonopore projecting as a flagellum, anteriorly extending along aedeagal apodemes, with an apical sclerite, sclerite highly variable in form within the genus. Aedeagal apodemes (temones) slightly longer than pedon, each posteriorly embedded in a lateral fold of pedon, sclerotized throughout, becoming wider and less sclerotized anteriorly.

#### Distribution.

Members of *Minyomerus* [JF2015] are distributed across the desert and grassland regions of North America (Figs 50–52). They are found as far north as Alberta, Canada, and south to Baja California Sur and San Luis Potosí, Mexico. Their range extends from the western Mojave Desert to Missouri.

#### Natural history.

*Minyomerus* [JF2015] species have a range of host plants with which they can be associated; they are commonly found on creosote bush (*Larrea tridentata* (DC.) Coville [non-focal; Zygophyllaceae [non-focal]), broomweed (*Gutierrezia* Lagasca [non-focal; Asteraceae [non-focal), sagebrush (*Artemisia* Linnaeus [non-focal]; Asteraceae [non-focal]), and occasionally on other various Asteraceae [non-focal]. It is likely that the larvae are root feeders, as are other tanymecine [non-focal] weevils, but this has not been directly observed. Adults are found on the stems and leaves of their host plant during day and night. Some species of *Minyomerus* [JF2015] are active during the hottest parts of the day; others might possibly take shelter near the roots of the host. Many species have overlapping distributions and can occur sympatrically in certain areas, and in many cases on the same individual plants. They can be collected by beating, sweeping plants, light trapping, and with pitfall traps.

### Minyomerus imberbus [JF2015]

Taxon classificationAnimaliaColeopteraCurculionidae

Jansen & Franz sec. Jansen & Franz (2015) sp. n.

http://zoobank.org/CA1E4402-FF39-4FB2-9FAE-01EF1818BD43

Figs 5 , 6

#### Diagnosis.

*Minyomerus imberbus* [JF2015] is best distinguished from other congenerics by a combination of characters, as follows. The interspersed setae on the body are minute and white. The anterior margin of the pronotum bears a reduced tuft of post-ocular vibrissae. The head is barely elevated between the eyes and head appears bare and smooth due to the small size of the setae. The spermatheca is distinct and has an elongate, cylindrical ramus, which is slightly thinner than corpus. Finally, the cornu is strongly recurved in the basal half, giving it a uniquely sinuate appearance.

#### Description – female.

*Habitus.* Length 3.69-5.82 mm, width 1.52–2.72 mm, length/width ratio 2.14–2.43, widest at anterior 1/4 of elytra. Integument dark reddish-brown to black. Scales slightly off-white to beige, semi-translucent or opaque. Setae minute, sub-recumbent, white.

*Mandibles.* Covered with whitish scales, with 2–3 longer setae.

*Rostrum.* Length 0.45–0.61 mm, anterior portion ca. 2.5 × broader than long, rostrum/pronotum length ratio 0.41–0.52, rostrum length/width ratio 1.21–1.24. Dorsal outline of rostrum square, anterior half of dorsal surface mesally concave, posterior half slightly convex. Rostrum in lateral view sub-rectangular; apical margin with 2 large vibrissae. Nasal plate defined by V-shaped, impressed lines, convex, integument covered with white scales. Margins of mandibular incision directed 20° outward dorsally in frontal view. Ventrolateral sulci strongly defined, beginning as a narrow sulcus posteriad of insertion point of mandibles, running parallel to scrobe, terminating in a ventral fovea. Ventrolateral margins slightly converging anteriorly.

*Antennae.* Dorsal margin of scrobe overhanging broadly (not forming a minute tooth). Funicle slightly longer than scape. Club similar in length to funicular antennomeres IV-VII, ca. 2.5 × as long as wide.

*Head.* Eyes separated in dorsal view by 4–5 × their anterior-posterior length, set off from anterior prothoracic margin by ca. 1/2 of their anterior-posterior length. Head between eyes slightly convex and rugoso-punctate.

*Pronotum.* Wider than long, length/width ratio 0.89–0.96, somewhat globular; median sulcus not apparent. Anterior margin arcuate, subtly incurved mesally, and somewhat produced dorsally; anterior constriction broad, posterior margin slightly arcuate. Pronotum in lateral view with minute setae inserted 2 × their length from anterior margin. Anterolateral margin with a reduced tuft of post-ocular vibrissae present, consisting of 3–5 setae, emerging near ventral 1/4 of eye; vibrissae achieving a maximum length sub-equal to anterior-posterior length of eye.

*Scutellum.* Usually exposed, lateral margins strongly incurved.

*Pleurites.* Metepisternum covered by elytron near posterior margin of metasternum.

*Thoracic sterna.* Mesocoxal cavities separated by distance 1/2 × width of mesocoxal cavity. Metasternum without apparent transverse sulcus; metacoxal cavities separated by 3 × their width.

*Legs.* Profemur/pronotum length ratio 0.78–1.05; proximal 5/6 of profemur gradually widening, then abruptly constricted with distal 1/6 produced ventrally as a semicircular projection covering tibial joint; condyle of tibial articulation occupying 3/4 of distal surface and 1/6 length of femur. Protibia/profemur length ratio 0.86–0.93; protibia moderately stout; protibial apex with ventral setal comb situated on a slightly concave surface; mucro present as a minute, laterally projected tooth. Metatibial apex with almond shaped convex ity ringed by 12–14 short, spiniform setae.

*Elytra.* Length/width ratio 2.85–3.05; widest at anterior 1/4; anterior margins jointly ca. 2 × wider than posterior margin of pronotum; lateral margins slightly converging after anterior 1/4, more strongly rounded and converging in posterior 1/3. Elytra in lateral view nearly planar in region of disk, concave near anterior margin and posterior declivity; posterior declivity angled at 65–70° to main body axis. Punctures distinct, separated by 3–6 × their diameter; intervals weakly elevated.

*Abdominal sterna.* Ventrite III elevated and set off from IV along lateral 1/3s of its length, somewhat concave anteriorly. Sternum VII mesally 3/5 as long as wide; anterior margin straight.

*Tergum.* Pygidium (tergum VIII) sub-cylindrical.

*Sternum VIII*. Lamina sub-triangular; sclerotized region with pores throughout, more sclerotized medially.

*Ovipositor.* Coxites 1/2 as broad as long in dorsal view, porose throughout; styli glabrous.

*Spermatheca.* S-shaped; collum short, apically with a large, hood-shaped projection perpendicular to ramus, nearly equal in length and contiously aligned with curvature of bulb of ramus and ante-apically with a short, cylindrical projection, angled at ca. 45° to collum, nearly equal in length to collum and 1/3 length of ramus; collum short, obliquely rounded, sub-contiguous with, and angled at 90° to ramus; ramus elongate, cylindrical, slightly thinner than corpus; corpus not swollen, slightly thicker than ramus; cornu elongate, apically, gradually narrowed, strongly recurved and arched in basal 1/2, forming an inner angle of ca. 40°, with apical 1/2 perpendicular to collum and corpus, feebly sinuate.

**Figure 5.**
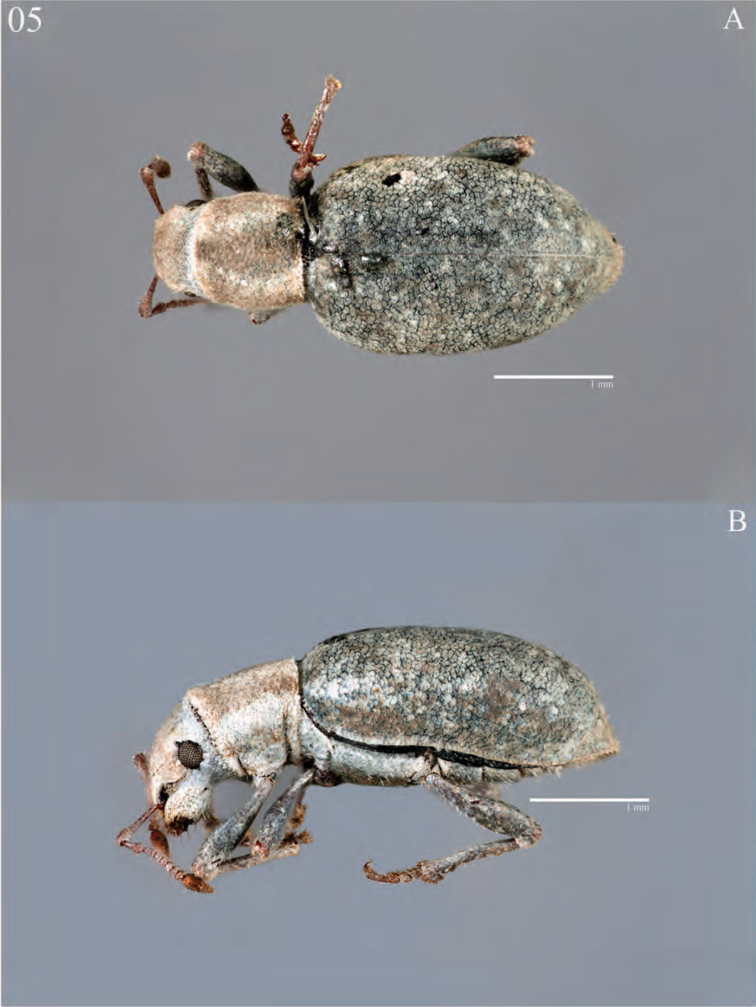
Habitus of *Minyomerus imberbus* [JF2015], female **A** dorsal view **B** lateral view.

**Figure 6.**
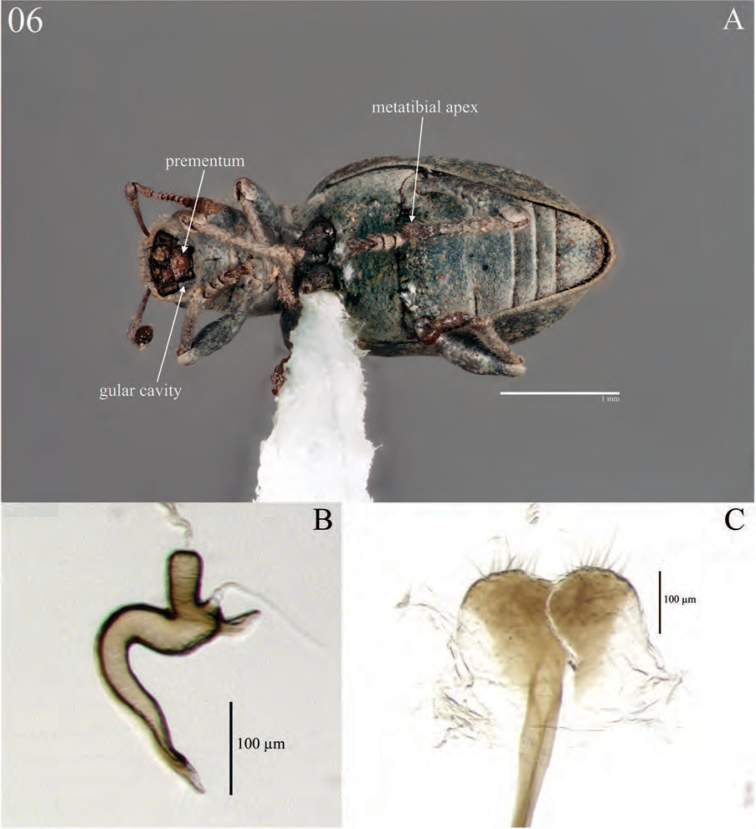
Diagnostic features and terminalia of *Minyomerus imberbus* [JF2015], female **A** ventral habitus **B** spermatheca **C** lamina of sternum VIII.

#### Male.

Not available or known.

#### Comments.

Due to the limited number of specimens of this species, dissections of mouthparts could not be performed.

#### Etymology.

Named in reference to the relative lack of setae on the head; *imberbus* = beardless; Latin adjective (Brown 1956).

#### Material examined.

Holotype – female “Pine Valley IBP, Valid. Site, Millard Co., Ut., 27 Jun. 73” (BYU). Paratype – “Pine Valley IBP, Valid. Site, Millard Co., Ut., 27 Jun. 73/ Host *Artemisia spinescens* [non-focal]” (BYU: 1 female).

#### Distribution.

This species is known from one locality in Millard County, Utah (Fig. 50).

#### Natural history.

Associated with budsage (*Artemisia spinescens* D.C.Eaton [non-focal]; Asteraceae [non-focal]). It is unknown whether this species is parthenogenetic.

### Minyomerus constrictus [JF2015]

Taxon classificationAnimaliaColeopteraCurculionidae

(Casey, 1888) sec. Jansen & Franz (2015)

Figs 7 , 8

== AND = Elissa constricta Casey, 1888: 272 sec Casey (1888) (synonymized by Kissinger 1964: 30)

#### Diagnosis.

*Minyomerus constrictus* [JF2015] is most readily distinguished from other congenerics by the strong anterior “constriction” of the pronotum. The elytral setae are also very thin and setiform, especially on the disk, with setae apically tapering to a fine point, rather than being broadly rounded. The ocular vibrissae are well-developed in this species, and the eye is relatively large compared to other species. The spermatheca has the ramus bulbous, but tapered basally. The aedeagus is abruptly constricted apically.

#### Redescription – female.

*Habitus.* Length 3.33–4.17 mm, width 1.44–1.74 mm, length/width ratio 2.31–2.47, widest at anterior 1/3 of elytra. Integument black on tagmata and elytra, light to dark orange-brown on other appendages. Scales with variously interspersed colors ranging from white to manila/beige to coffee brown, in some specimens appearing semi-translucent (in others opaque) or with an opalescent sheen; scales on most specimens becoming lighter ventrally, but some specimens with venter of same coloration as dorsum. Setae very short, fine, white or translucent, becoming longer, thicker, and more erect on lateral margins of head and prothorax, humeri, and venter.

*Mandibles.* Covered with white scales, with 2 long setae dorsally, 2 long setae vetrolaterally, and 1 shorter seta between these.

*Maxillae.* Cardo bifurcate at base with an inner angle of ca. 125°, inner (mesal) arm 2 × longer than outer arm, inner arm 2 × width of outer arm, inner arm of bifurcation equal in length to apically outcurved arm. Stipes sub-quadrate, 1.25–1.5 × wider than long, roughly equal in length to inner arm of bifurcation of cardo, glabrous. Galeo-lacinial complex apically incurved (mesally); setose in basal 2/3; dorsally with 9 apicomesal lacinial teeth (1 of which is dorsad of mesal margin; ventrally with 5 reduced lacinial teeth. Palpiger with a transverse row of 2 setae, sclerotized on basal 2/3.

*Maxillary palps.* I and II both apically oblique, apical ends facing mesally and forming a 45° angle with base, I and II each with 2 apical setae.

*Labium.* Roughly trapezoidal; apical margins sinuate, weakly angulate; lateral margins straight; basal margin arcuate. Labial palps 3-segmented, I with apical 1/2 projecting beyond margin of prementum, reaching beyond apex of ligula; I and II both with 1 apical seta; III slightly longer than II.

*Rostrum.* Length 0.49–0.63 mm, anterior portion 2–3 × broader than long, rostrum/pronotum length ratio 0.59–0.64, rostrum length/width ratio 1.15–1.37. Dorsal outline of rostrum sub-rectangular, anterior half of dorsal surface impressed. Rostrum in lateral view sub-rectangular; basal half of dorsolateral margins strongly converging anteriorly, anterior half slightly divergent; apical margin with sinuations meeting mesally to form a small point, with 2 large vibrissae. Nasal plate defined by Y-shaped lines, concave mesally, covered white scales and with interspersed, erect, laterally directed setae. Margins of mandibular incision curved, directed 10–15° outward dorsally in frontal view. Ventrolateral sulci weakly defined as a broad concavity dorsad of insertion point of mandibles, running parallel to scrobe, becoming flatter posteriorly and disappearing ventrally. Dorsal surface of rostrum with median sulcus running from fovea at posterior end of anterior half rostrum to midpoint of posterior half of rostrum. Rostrum ventrally without median fovea. Oral cavity with lateral margins straight, diverging.

*Antennae.* Small tooth formed by overhanging dorsal margin of scrobe anterior to margin of eye by 1/2 of length of eye. Club nearly 3 × as long as wide.

*Head.* Eyes strongly impressed, posterior margin level with lateral surface of head; eyes separated in dorsal view by 3–4 × their anterior-posterior length, set off from anterior prothoracic margin by 1/4 of their anterior-posterior length.

*Pronotum.* Length/width ratio 0.80–0.93, globular; surface finely punctate; median sulcus absent. Anterior margin straight, lateral margins curved and widening into a bulge near anterior 2/5 of pronotum, thence curved towards posterior margin, posterior margin incurved. Pronotum in lateral view sub-cylindrical to slightly globular, with setae that just reach beyond anterior margin. Anterolateral margin with a tuft of post-ocular vibrissae present, emerging near dorsal 1/4 of eye, becoming gradually, evenly longer ventrally, stopping just above ventral 1/4 of eye; vibrissae achieving a maximum length 2/3 × anterior-posterior length of eye.

*Scutellum.* Equilateral to slightly longer than wide.

*Pleurites.* Metepisternum covered by elytron near anterior 1/5 of metasternum; a deep fovea present just basad of triangular projection of metepisternum.

*Thoracic sterna.* Mesocoxal cavities separated by distance1/3 × width of mesocoxal cavity. Metasternum with a transverse sulcus just posteriad of anterior margin, can be hidden by scales in some specimens; posteriorly a large median fovea between ventrite III and metasternum, antero-mesally planar, turned inward 25° and planar in posterior 1/2; metacoxal cavities widely separated by 3–4 × their width.

*Legs.* Profemur/pronotum length ratio 1.04–1.15. Protibia/profemur length ratio 0.87–0.94; protibia with ventral setal comb situated on a mesally incurved edge; mucro reduced to a very small apically projected tooth. Protarsus with tarsomere III equilateral; II broadly conical, III bifid; I and III jointly similar in length to V. Metatibial apex with almond shaped convex ity with a row of 5 short, widely separated, spiniform setae.

*Elytra.* Length/width ratio 2.90–3.20; widest at anterior 1/3; anterior margins jointly 1.5–2 × wider than posterior margin of pronotum; lateral margins sub-parallel after anterior 1/6, more strongly rounded and converging in posterior 1/4. Elytra in lateral view convex, mesally somewhat planar on dorsum; posterior declivity angled at nearly 80° to main body axis. Elytra very finely punctate, punctures not visible in some specimens, separated by 8 × their diameter; intervals not elevated.

*Abdominal sterna.* Scales similar to elytra, in some specimens of a lighter color; III with midregion planar posteriorly, anteriorly incurved to a mesal fovea on anterior margin, elevated and set off from IV along lateral 1/3 of its length. Sternum VII mesally 2/5 × as long as wide; setae darkening, lengthening, and becoming more erect in mesal 1/3; anterior margin weakly curved.

*Tergum.* Pygidium (tergum VIII) sub-cylindrical, medially with a patch of very short, fine setae.

*Sternum VIII.* Posterior 1/8 (lamina) square; anterior edges each incurved forming a 90° angle with lateral margin.

*Ovipositor.* Coxites in dorsal view nearly as long as broad; styli as long as coxites, glabrous.

*Spermatheca.* Comma-shaped; collum extremely short, apically with hood-shaped projection at 45° to central axis of ramus, 3/4 × length of ramus and contiously aligned with curvature of bulb of ramus; collum sub-contiguous with, and angled at 45° to ramus; ramus spherical, equal in length to width of corpus; corpus swollen, 1.5 × width of cornu; cornu elongate, apically, gradually narrowed, basal 3/4 angled at nearly 90° to corpus, gently curved along mesal 1/2, and recurved near apical 1/4 such that apex is sub-parallel to corpus.

**Figure 7.**
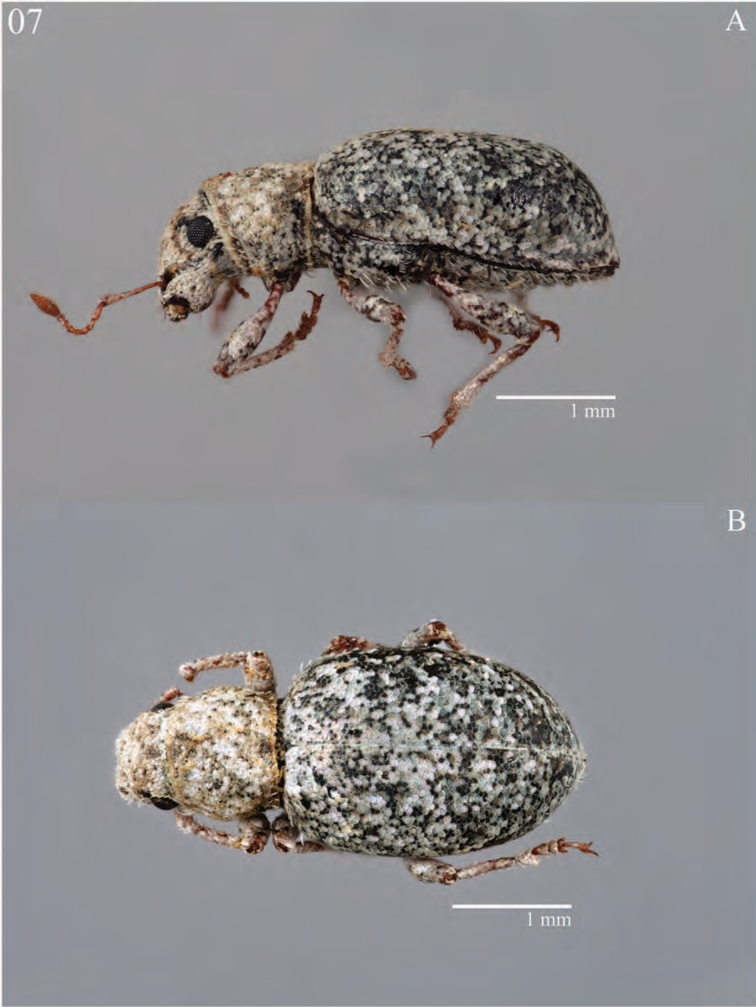
Habitus of *Minyomerus constrictus* [JF2015], female **A** lateral view **B** dorsal view.

**Figure 8.**
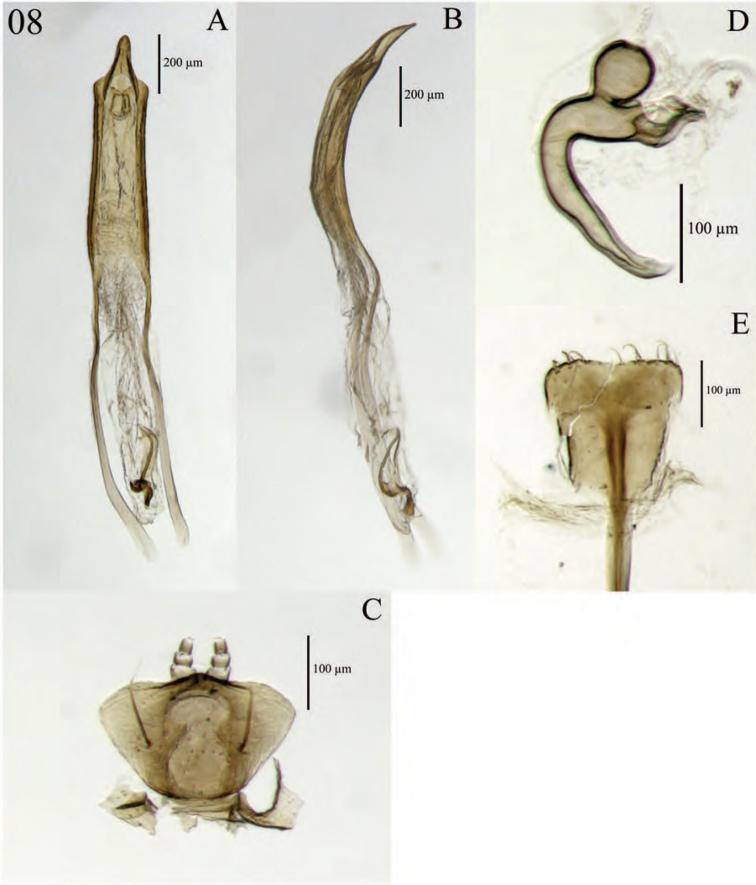
Diagnostic features and terminalia of *Minyomerus constrictus* [JF2015] **A** aedeagus, dorsal view **B** aedeagus, lateral view **C** labial prementum, ventral view **D** spermatheca **E** lamina of sternum VIII.

#### Male.

Similar to female, except where noted. Length 2.78–3.52 mm, width 1.12–1.38 mm, length/width ratio 2.49–2.63. Rostrum length 0.39–0.60, rostrum/pronotum length ratio 0.47–0.67, rostrum length/width ratio 1.15–1.55. Pronotum length/width ratio 0.82–0.91. Profemur/pronotum length ratio 0.83–1.16, protibia/profemur length ratio 0.92–0.97. Elytra length/width ratio 2.97–3.34.

*Elytra.* Generally narrower relative to pronotum, but otherwise as female.

*Abdomen.* Sternum VII slightly more broadly arcuate posteriorly, 3/5 as long as wide. Pygidium (tergum VIII) with posterior margin evenly arcuate; posterior 1/2 punctate; anterior 1/2 rugose.

*Sternum VIII.* Consisting of 2 sub-contiguous, sub-triangular sclerites; posterior margins widely angulate; without median, sub-trapezoidal projection.

*Aedeagus.* Length/width ratio 3.54–3.76; lateral margins very slightly converging posteriorly, abruptly constricted and more strongly converging in region of ostium. In lateral view, width of pedon becoming gradually narrower posteriorly in anterior 3/4, ventral margins in posterior 1/4 becoming straight towards apex, then abruptly curving to meet dorsal margins at a sharp apical point; apex acutely angulate. Flagellum apically with a large, narrowly elongate, tortuous, ampullate sclerite, sclerite anteriorly gradually widened, constricted in anterior 1/4 and slightly widening anteriorly to form a small bulb.

#### Material examined.

Holotype – female “●Ari [Casey locality code: Yuma Gila Valley, December 1886]/ Casey bequest 1925/ Type USNM 35006/ *Elissa constricta* Cas.” (USNM). Additional specimens examined: “Cal. Kelso Sand Dunes, S. Berdo. Co. IV-7-1966, L & C.W. O’Brien” (CWOB: 2 females, 2 males); “CAL. S. Berdo. Co. 3 mi. NW. Vidal W. Gagne, III-17-1967” (CWOB: 3 females, 1 male); “Ariz. 13 mi. S. Ajo, Pima Co., IV-2-1966, L & C.W. O’Brien/ *Larrea tridentata* [non-focal]” (CWOB: 8 females, 4 males) [4 females, 2 males deposited at CMNC]; “Boulder Dam, Ariz/ Collection of Vasco M. Tanner” (CAS: 1 female, 2 males; BYU: 3 females, 1 male); “CALIF: Inyo Co., Eureka Valley, January 1978, Giuliani, Hardy, and Andrews/ Eureka Valley, transect anti-freeze pit trap, no. 8” (CSCA: 1 female, 2 males); “CALIF: Inyo Co., Eureka Valley, January 1978, Giuliani, Hardy, and Andrews/ Eureka Valley, transect anti-freeze pit trap, no. 8” (CSCA: 1 female, 2 males); “CALIF: Inyo Co., Eureka Valley, February 1978, Giuliani, Hardy, and Andrews/ Eureka Valley, transect anti-freeze pit trap, no. 7 [part of series, includes 7 & 8]” (CSCA: 1 female, 1 male); “CALIF: Inyo Co., Eureka Valley, March 1978, Giuliani, Hardy, and Andrews/ Eureka Valley, transect anti-freeze pit trap, no. 8” [part of series, includes 8, 9, & two individuals from 20] (CSCA: 2 females, 2 males); “CALIF: Inyo Co., Eureka Valley, April 1978, Giuliani, Hardy, and Andrews/ Eureka Valley, transect anti-freeze pit trap, no. 7” [part of series, includes three individuals from 7 & two individuals from 8] (CSCA: 5 females); “CALIF: Inyo Co., Eureka Valley, March 1978, Giuliani, Hardy, and Andrews/ Eureka Valley, transect anti-freeze pit trap, no. 8” [part of series, includes 8, 9, & two individuals from 20] (CSCA: 2 females, 2 males); “El Cajon, San Diego Co., CA. 18 Feb 71/ Ex. *Pyrus kawakami* [non-focal]/ J.F. Johnson Coll.” (CSCA: 1 female).

#### Distribution.

This species has been found in the desert and arid regions of Arizona and California (USA). It is likely that its range also includes Nevada (USA), northern Baja California and Sonora (Mexico), based on similarity in habitat to the currently known distribution (Fig. 50).

#### Natural history.

Associated with creosote bush (*Larrea tridentata* [DC.] Coville [non-focal]; Zygophyllaceae [non-focal]) and evergreen pear (*Pyrus kawakami* Tang S. Liu & H.J. Su [non-focal]; Rosaceae [non-focal]).

### Minyomerus laticeps [JF2015]

Taxon classificationAnimaliaColeopteraCurculionidae

(Casey, 1888) sec. Jansen & Franz (2015)

Figs 9 , 10 , 11

== AND = Elissa laticeps Casey, 1888: 272 sec. Casey (1888)

#### Diagnosis.

*Minyomerus laticeps* [JF2015] is most easily distinguished from other congenerics by the unique shape of the head, which is very wide and only somewhat swollen between the eyes. The rostrum is also much wider than long, and the nasal plate is weakly impressed. Additionally, the anterior margin of the prothorax is larger than the posterior margin and bears a well-developed tuft of post-ocular vibrissae. The spermatheca is unique in having the ramus stalked, with the apical bulb abruptly constricted, not tapering, at the point of connection with the stalk. The small, sub-recumbent setae and lack of separation of the procoxae help to distinguish this species from *Minyomerus griseus* [JF2015] and *Minyomerus rutellirostris* [JF2015].

#### Redescription – female.

*Habitus.* Length 3.90–5.74 mm, width 1.51–2.22 mm, length/width ratio 2.37–2.63, widest at anterior 1/4 of elytra. Integument tan, orange-brown to dark brown. Scales with variously interspersed colors ranging from slightly off-white to manila/tan to dark coffee brown, in some specimens appearing semi-translucent (in others opaque). Setae sparse throughout, short, sub-recumbent.

*Mandibles.* Covered with opalescent scales, with 4 longer setae, and a number of shorter interspersed setae.

*Maxillae.* Cardo bifurcate at base with an inner angle of ca. 90°, arms of equal length, inner (mesal) arm 1.5 × thicker than outer arm, both arms of bifurcation equal in length to apically outcurved arm. Stipes sub-quadrate, slightly longer than wide, roughly equal in length to each bifurcation of cardo, glabrous. Galeo-lacinial complex mesally membranous, laterally sclerotized, with sharp demarcation of sclerotized region separating palpiger from galeo-lacinial complex; setose in membranous area just adjacent to sclerotized region, setae covering half of dorsal surface area; dorsally with 8 apicomesal lacinial teeth; ventrally with 5 reduced lacinial teeth. Palpiger with a row of setae extending laterally along basal 1/2, thereafter becoming abruptly transverse; anterior 1/2 membranous.

*Maxillary palps.* I and II both apically oblique, apical ends forming a 45° angle with base, I and II each with 2 apical setae; II with 1 mesoventral seta in addition to 2 apical setae.

*Labium.* Prementum roughly hexagonal; apical margin laterally incurved, angulate. Labial palps 3-segmented, II with apical 1/3 projecting beyond margin of prementum, but not reaching apex of ligula; III slightly longer than II.

*Rostrum.* Length 0.52–0.73 mm, anterior portion 3–4 × broader than long, rostrum/pronotum length ratio 0.53–0.61, rostrum length/width ratio 0.73–0.89; shape in cross section elongate-rectangular. Dorsal outline of rostrum sub-rectangular, posterior half of dorsal surface strongly rugose. Rostrum in lateral view nearly square; apical margin with 2 large vibrissae. Nasal plate weakly defined by V-shaped, impressed lines, slightly convex, with opalescent scales. Margins of mandibular incision directed 15° outward dorsally in frontal view; ventrolateral sulci usually weakly defined or obscure, if strongly defined then only as a notch dorsad of insertion point of mandibles. Dorsal surface of rostrum transversely impressed (strongly or weakly in some specimens), with median sulcus running from fovea at posterior end of rostrum to imaginary transverse line between anterior margins of eyes. Oral cavity with lateral margins feebly curved.

*Antennae.* Scrobe continuing to anterior 1/2 of eye; small tooth formed by overhanging dorsal margin of scrobe ventrad of anterior 1/3 of eye. Scape nearly extending to posterior margin of eye. Terminal funicular antennomere lacking appressed scales, having instead a covering of apically-directed pubescence with interspersed sub-erect setae. Club nearly 3 × as long as wide, with setation and pubescence as in final segment of funicle.

*Head.* Eyes with posterior margin slightly elevated from lateral surface of head; eyes separated in dorsal view by 4 × their anterior-posterior length, set off from anterior prothoracic margin by 1/3 of their anterior-posterior length. Head between eyes rugose and slightly bulging, appearing flat in some specimens. Head without any transverse post-ocular impression.

*Pronotum.* Length/width ratio 0.75–0.89, sub-cylindrical to slightly globular; widest after midpoint before anterior constriction. Anterior margin nearly straight, subtly incurving mesally, lateral margins feebly curved, posterior margin incurved. Pronotum in lateral view with setae that barely reach beyond anterior margin. Anterolateral margin with a tuft of post-ocular vibrissae present, emerging near dorsal margin of eye, becoming gradually, evenly longer ventrally, stopping just above ventral margin of eye; vibrissae achieving a maximum length nearly as long as anterior-posterior length of eye.

*Pleurites.* Metepisternum covered by elytron near posterior 1/4 of metasternum.

*Thoracic sterna.* Mesocoxal cavities separated by distance 1/3–2/5 × width of mesocoxal cavity. Metasternum with transverse sulcus usually apparent (faint in some specimens); metacoxal cavities widely separated by 3–4 × their width.

*Legs.* Profemur/pronotum length ratio 0.96–1.09; distal 1/5 of profemur produced ventrally as a semicircular projection covering tibial joint. Protibia/profemur length ratio 0.88–0.91; protibia with ventral setal comb recessed in a broadly concave groove, setal comb unbroken, but becoming thinner and sparser anteriorly; mucro reduced to a very small laterally projected tooth. Protarsus with tarsomeres II and III similar in length, equilateral. Metatibial apex with almond shaped convex ity ringed by 8 short, widely separated, spiniform setae.

*Elytra.* Length/width ratio 2.74–2.95; widest at anterior 1/4; anterior margins jointly 1.25–1.50 × wider than posterior margin of pronotum; lateral margins sub-parallel after anterior 1/4, more strongly rounded and converging in posterior 1/2. Posterior declivity angled at nearly 70° to main body axis. Elytra with striae shallow, punctate, punctures faint beneath appressed scales, separated by 3–4 × their diameter; intervals very slightly elevated.

*Abdominal sterna.* Ventrite III elevated and set off from IV along lateral 1/4s of its length, anteriorly planar. Sternum VII mesally 3/5–2/3 × as long as wide; setae darkening, lengthening, and becoming more erect in posterior 1/4; anterior margin weakly curved.

*Tergum.* Pygidium sub-cylindrical; medial 1/5 of anterior 2/3 of pygidium less sclerotized, with a patch of very short, fine setae.

*Sternum VIII.* Anterior laminar edges of spiculum ventrale each incurved forming a 110° angle with lateral margin; lamina more sclerotized medially. *Ovipositor.* Coxites nearly as broad as long.

*Spermatheca.* ?-shaped; collum short, apically with a large, hood-shaped projection sub-parallel to ramus, nearly equal in length and contiously aligned with curvature of bulb of ramus; collum sub-contiguous with, and angled at 45°-60° to ramus; ramus basally elongate, forming a stalk (length variable among examined specimens), bulbous apically, 4 × thicker than stalk; corpus not swollen, of equal thickness to collum and cornu; cornu elongate, apically, gradually narrowed, strongly recurved in basal 1/4, straight along mesal 1/2, and curved near apical 1/4 such that apex is parallel to collum and corpus.

**Figure 9.**
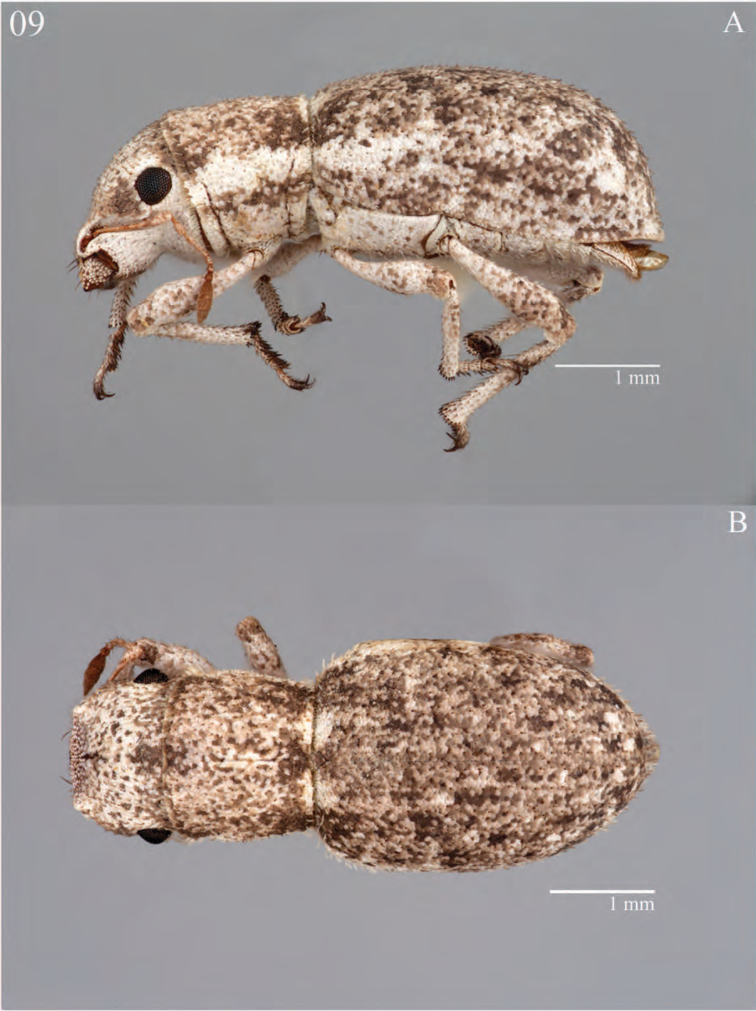
Habitus of *Minyomerus laticeps* [JF2015], female **A** lateral view **B** dorsal view.

**Figure 10.**
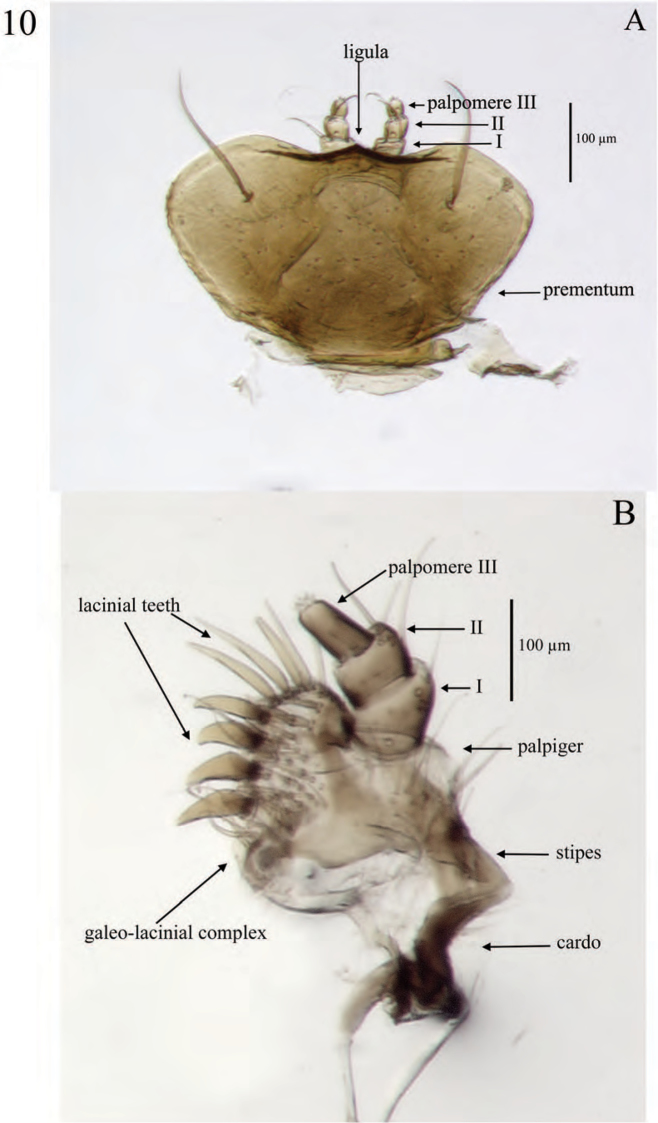
Mouthparts of *Minyomerus laticeps* [JF2015] **A** labial prementum, ventral view **B** left maxilla, ventral view.

**Figure 11.**
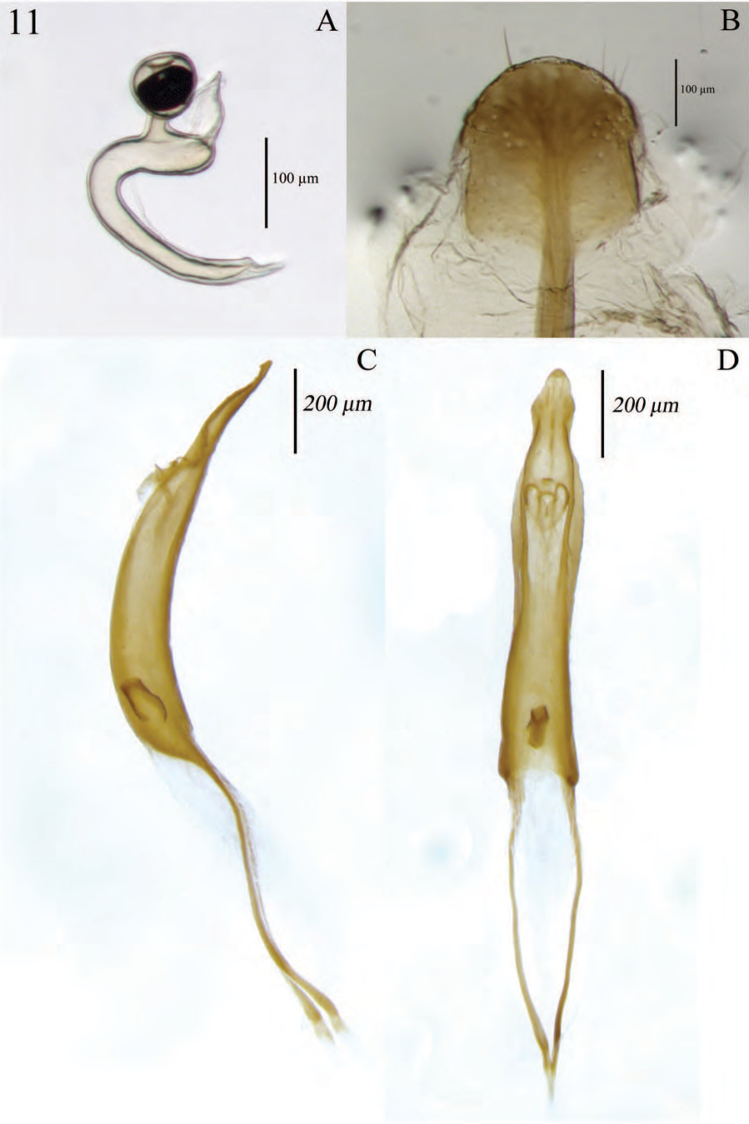
Terminalia of *Minyomerus laticeps* [JF2015], female **A** spermatheca **B** lamina of sternum VIII **C** aedeagus, lateral view **D** aedeagus, dorsal view.

#### Male.

Similar to female, except where noted. Length 3.50–4.67 mm, width 1.26–1.70 mm, length/width ratio 1.77–1.96. Rostrum length 0.60–0.77 mm, rostrum/pronotum length ratio 0.53–0.61, rostrum length/width ratio 0.73–0.89. Pronotum length/width ratio 0.75–0.82. Profemur/pronotum length ratio 1.17–1.25, protibia/profemur length ratio 0.85–0.96. Elytra length/width ratio 2.88–3.10.

*Elytra.* Generally shorter and narrower relative to pronotum, but otherwise as female.

*Abdomen.* Sternum VII slightly more broadly arcuate posteriorly, 1/2 -3/5 as long as wide. Pygidium (tergum VIII) with mesal 1/3 of posterior margin nearly straight; posterior 1/2 punctate; anterior 1/2 rugose.

*Aedeagus.* Length/width ratio 4.25–4.71; lateral margins very slightly converging posteriorly, diverging in region of ostium, then converging into a large, spadiform projection that is roughly the size of the ostium. In lateral view, width of pedon becoming gradually narrower posteriorly in anterior 2/3, ventral margins in posterior 1/3 becoming straight towards apex; apex acutely angulate. Flagellum apically with a small, sub-cylindrical sclerite, sclerite posteriorly with two small, lateral flanges.

#### Comments.

Specimens can vary somewhat in color, from dark brown to beige. Some specimens exhibit a relatively narrower prothorax, depending on the locality. The bulging of the head and form of the rostrum can also vary slightly, with some specimens exhibiting a smaller rostrum and more bulbous head.

#### Material examined.

Holotype – female “●Tex [Casey locality code: El Paso including both banks of the River Grande which is here about 100 ft. in width]/ Casey bequest 1925/ Type USNM 35005 [red]/ *Elissa laticeps* Cas” (USNM). Syntypes – “Ari● [Casey locality code: Benson (Collected by G.W. Dunn)]/ Type USNM ? Syntype [red]/ *Elissa laticeps* 10785 Casey” (USNM: 1 female); “Ari●/ Casey bequest 1925/ Casey determ. *laticeps*-2 [part of series, ranging 2-6]/ Type USNM Syntype ? [red]” (USNM: 5 females). Additional specimens examined: “Cochise Co., Ariz, T17S-R31-E-3--, 27 VIII 59, 092/ *Minyomerus laticeps*, det. D.G. Kissinger” (CSCA: 3 females); “N. Mex. 17 mi. NE. Las Cruces, Jornada Expt. Rge. X-3-1970/ *Larrea tridentata* [non-focal] at night, C.W. O’Brien/ Compared with type *Elissa laticeps* Csy 1977 [green]” (CWOB: 8 females); “TEX. Big Bend N.P. Grapevine Hills Ranch, III-27-1970/ on mesquite, C.W. O’Brien” (CWOB: 1 female); “TEX. 57 mi. S. Alpine, Brewster Co. 6-6-1970/ Collectors: L&C.W. O’Brien” (CWOB: 1 female); “AZ, Pima Co. 1 mi. N. Hwy 86, 2 mi. S, San Pedro, Sept. 8 2008 CW. O’Brien/ on *Larrea tridentata* [non-focal]” (CWOB: 4 females); “Ariz.: Coch. Co., 5 mi N Portal, IX-21-1976, DS Chandler” (CWOB: 1 female); “MEX., Coah. 23 mi. W. Saltillo 4200’, night 8-20-1971, O’Briens & Marshall” (CWOB: 59 females) [15 females deposited at CMNC]; “MEX., Coah. 4 mi. W. Saltillo 5600’, night 8-20-1971, O’Briens & Marshall” (CWOB: 3 females); “MEX., Coah. 13 mi. W. Saltillo night, 4850’ 8-20-1971, O’Briens & Marshall” (CWOB: 3 females); “MEX. 14 mi. W. Monterrey, x-18-1970, C.W. O’Brien” (CWOB: 5 females); “Presidio Tex, X-28-44, JHRussel/ on *Larrea divaricata* [non-focal], foliage, 44-26374” (USNM: 10 males, 10 females).

#### Distribution.

This species has been found in the desert and arid regions of Arizona, New Mexico, Texas (USA), Nuevo Leon, Coahuila, and Baja California Sur (Mexico). It is likely that its range also includes southern California (USA), Baja California, northern Chihuahua, and Sonora (Mexico), based on similarity in habitat to the currently known distribution (Fig. 50).

#### Natural history.

Associated with creosote bush (*Larrea tridentata* [DC.] Coville [non-focal]; Zygophyllaceae [non-focal]) and mesquite (*Prosopis* [non-focal] spp.; Fabaceae [non-focal]).

### Minyomerus conicollis [JF2015]

Taxon classificationAnimaliaColeopteraCurculionidae

Green, 1920 sec. Jansen & Franz (2015)

**Figure 12.**
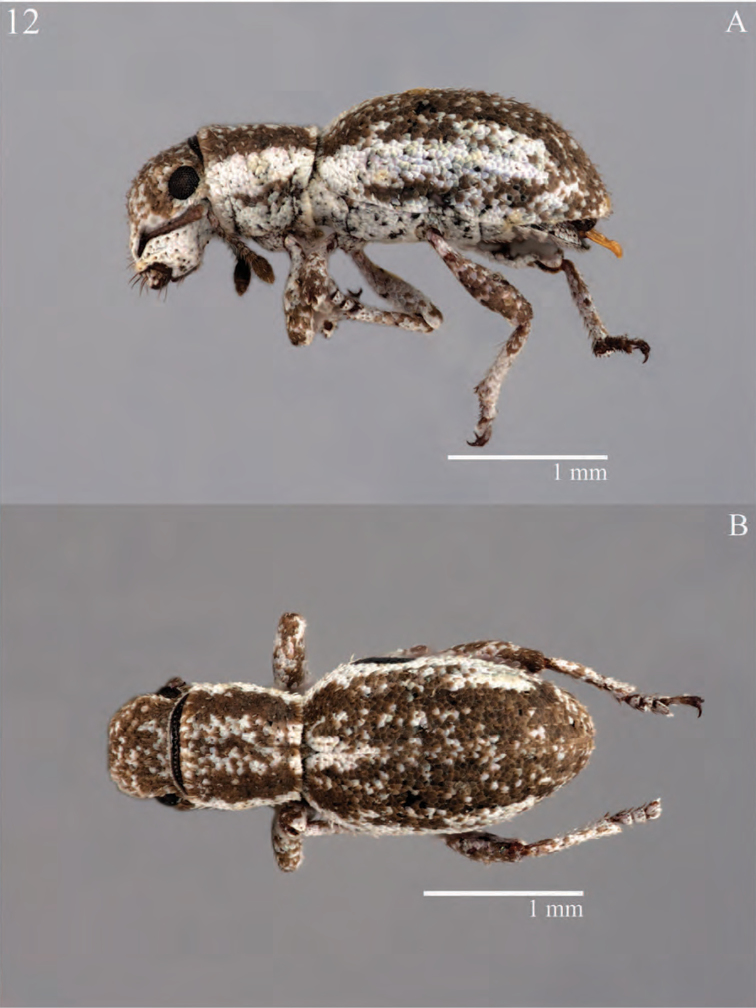
Habitus of *Minyomerus conicollis* [JF2015], male **A** lateral view **B** dorsal view.

**Figure 13.**
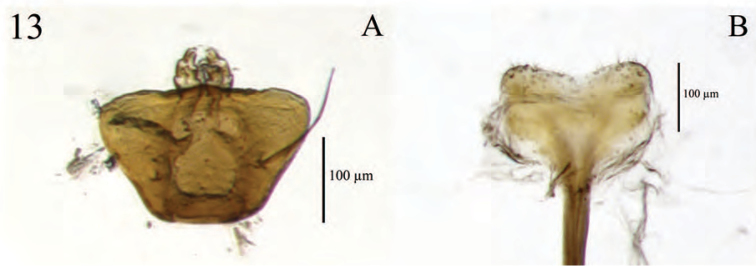
Diagnostic features and terminalia of *Minyomerus conicollis* [JF2015] **A** labial prementum, ventral view **B** lamina of sternum VIII.

**Figure 14.**
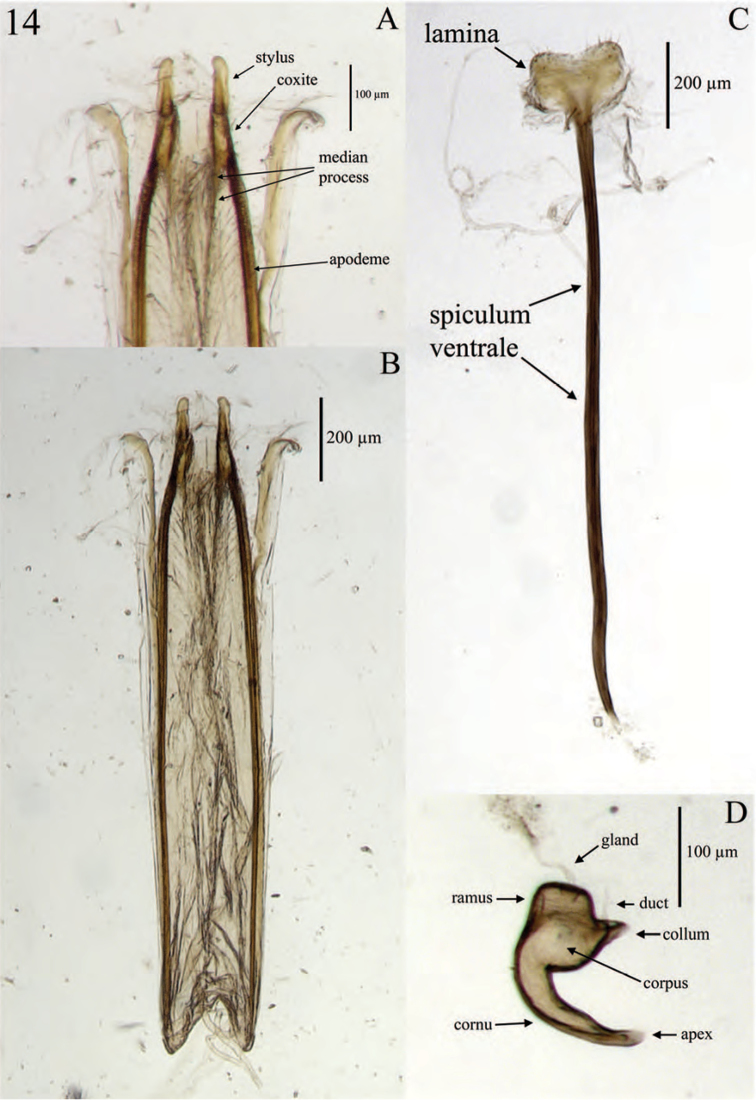
Terminalia of *Minyomerus conicollis* [JF2015], female **A** ovipositor, apex, dorsal view **B** ovipositor, dorsal view **C** sternum VIII **D** spermatheca.

**Figure 15.**
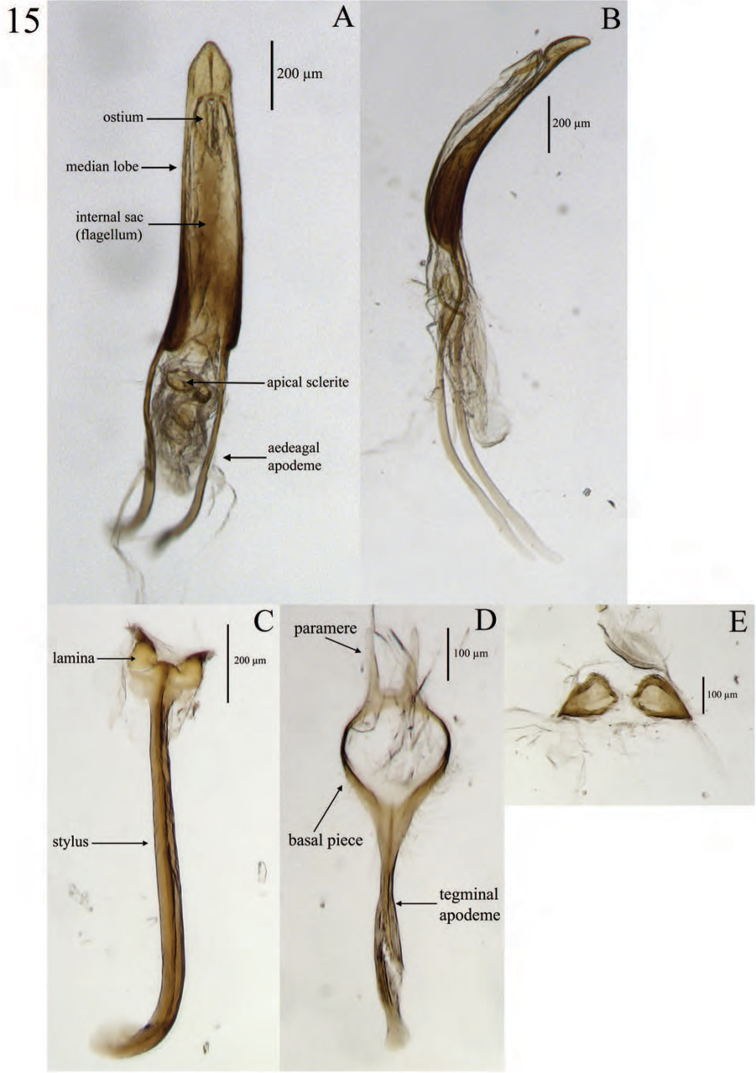
Terminalia of *Minyomerus conicollis* [JF2015], male **A** aedeagus, dorsal view **B** aedeagus, lateral view **C** spiculum gastrale **D** aedeagal tegmen **E** sternum VIII.

Figs 12 , 13 , 14 , 15

== Minyomerus conicollis Green, 1920: 194 sec. Green (1920)

#### Diagnosis.

*Minyomerus conicollis* [JF2015] is readily distinguished from other congenerics by the highly convex elytra, and by the pronotum, in which the posterior margin is distinctly shorter than the anterior margin. The spermatheca is uniquely comma-shaped, with the ramus reduced, apically flattened and sub-contiguous with the collum. The aedeagus is unique in being membranous ventrally, and not fully sclerotized.

#### Redescription – female.

*Habitus.* Length 2.80–3.42 mm, width 1.02–1.27 mm, length/width ratio 2.64–2.76, widest at anterior 1/5–1/4 of elytra. Integument orange-brown to black. Scales with variously interspersed colors ranging from white to manila/tan to dark coffee brown, in some specimens appearing semi-translucent (in others opaque) or with metallic reflections. Setae sub-recumbent.

*Mandibles.* Covered with opalescent scales, with 6 longer setae.

*Maxillae.* Cardo bifurcate at base with an inner angle of ca. 100°, inner (mesal) arm 2 × longer than outer arm, inner arm of equal width to outer arm, inner arm of bifurcation equal in length to apically outcurved arm. Stipes square, roughly equal in length to inner arm of bifurcation of cardo, with a single lateral seta. Galeo-lacinial complex apically incurved (mesally); complex membranous; setose in basal 2/3; dorsally with 8 apicomesal lacinial teeth; ventrally with 3 reduced lacinial teeth. Palpiger with a transverse row of 3 setae, sclerotized on basal 2/3.

*Maxillary palps*. Palpomeres I and II with apical ends facing mesally and forming a 45° angle with base, each with 1 apical seta; II with 1 mesoventral seta in addition to apical seta.

*Labium.* Prementum roughly trapezoidal, ventrally sub-planar throughout; apical margins sinuate, strongly angulate; lateral margins incurved; basal margin mesally nearly straight. Labial palps 3-segmented, I with apical 1/2 projecting beyond margin of prementum, reaching past apex of ligula; III slightly longer than II.

*Rostrum.* Length 0.40–0.48 mm, anterior portion 2–2.25 × broader than long, rostrum/pronotum length ratio 0.56–0.69, rostrum length/width ratio 1.01–1.24. Dorsal outline of rostrum sub-rectangular, anterior half of dorsal surface strongly impressed, posterior half sometimes weakly rugose. Rostrum in lateral view rectangular; apical margin with 2 large vibrissae. Nasal plate strongly defined by V-shaped, impressed lines, convex, integument completely covered with scales similar to those of body. Margins of mandibular incision directed 30° outward dorsally in frontal view; ventrolateral sulci usually undefined or weakly defined, beginning as a broad, shallow sulcus dorsad of insertion point of mandibles, running parallel to scrobe, becoming fainter posteriorly and disappearing ventrally; occasionally with a short, well defined sulcus dursad of insertion point of mandibles. Dorsal surface of rostrum with median sulcus present as a short, linear fovea posteriad of base of nasal plate; ventrolateral margins slightly converging anteriorly. Rostrum ventrally lacking sulci at corners of oral cavity. Oral cavity with lateral margins strongly curved.

*Antennae.* Dorsal margin of scrobe overhangs slightly (broadly, not forming a sharp tooth) ventrad of anterior margin of eye. Club similar in length to funicular antennomeres IV-VII, nearly 2.5 × as long as wide.

*Head.* Eyes separated in dorsal view by 4 × their anterior-posterior length, touching anterior prothoracic margin.

*Pronotum.* Slightly, but distinctly, longer than wide, length/width ratio 0.84–0.90, sub-conical; widest just anteriad of midline; median sulcus present, sometimes obscure. Anterior margin arcuate, subtly incurved medially, lateral margins feebly curved and widening into a slight bulge just anteriad of midline, thence straight to posterior margin, posterior margin straight and shorter than anterior margin. Pronotum in lateral view with setae that just reach anterior margin at their maximum length. Anterolateral margin with a reduced tuft of post-ocular vibrissae present, consisting of 5–7 setae, emerging near ventral 1/3 of eye, becoming gradually, evenly longer ventrally, stopping just beneath ventral margin of eye; vibrissae achieving a maximum length 2/3–3/4 × anterior-posterior length of eye.

*Scutellum.* 1.5–2 × longer than wide at base, margins straight.

*Pleurites.* Metepisternum covered by elytron near posterior margin of metasternum.

*Thoracic sterna.* Mesocoxal cavities separated by distance 1/4–1/3 × width of mesocoxal cavity. Metasternum without apprent transverse sulcus; metacoxal cavities widely separated by 1.75–2.25 × their width.

*Legs.* Profemur/pronotum length ratio 0.98–1.06; profemur with distal 1/5 produced ventrally as a nearly square projection covering tibial joint. Protibia/profemur length ratio 0.83–0.90; mucro reduced to a small laterally projected tooth. Protarsus with tarsomere I nearly 1.5 × as long as II; I and III similar in length, III equilateral; I-III jointly similar in length to V. Metatibial apex with almond shaped convex ity ringed by 7 short, widely separated, spiniform setae.

*Elytra.* Length/width ratio 3.21–3.46; widest at anterior 1/5–1/4; anterior margins jointly 2 × wider than posterior margin of pronotum; lateral margins sub-parallel after anterior 1/5, more strongly rounded and converging in posterior 1/2. Elytra in lateral view sculpted with a depression at anterior 1/3; posterior declivity angled at nearly 60° to main body axis. Elytral striae defined, punctate; punctures visible, deep and distinct in some specimens, separated by 3–6 × their diameter.

*Abdominal sterna.* Ventrite III elevated and set off from IV along lateral 1/4–1/3s of its length. Sternum VII mesally 1/2 × as long as wide, with a strong medial concavity; anterior margin weakly curved.

*Tergum.* Pygidium (tergum VIII) sub-cylindrical; medial 1/3 of anterior 2/3 of pygidium less sclerotized.

*Sternum VIII.* Anterior laminar edges each incurved forming a 100° angle with lateral margin; less sclerotized medially between arms; posterior edge medially incurved.

*Ovipositor.* Coxites slightly sclerotized, in dorsal view 2 × as long as broad.

*Spermatheca.* Comma-shaped; collum short, apically with a reduced hood-shaped projection; collum sub-contiguous with, and angled at 90° to ramus; ramus bulbous, slightly larger than collum and corpus; corpus swollen, of nearly equal thickness ramus and cornu; cornu elongate, apically gradually narrowed, strongly recurved in basal 1/4, gently curved along mesal 1/2, and curved and somewhat flattened near apical 1/4 such that apex is sub-parallel to hood-shaped projection of collum.

#### Male.

Similar to female, except where noted. Length 2.41–2.85 mm, width 0.90–0.99 mm, length/width ratio 2.53–2.94. Rostrum length 0.38–0.48 mm, rostrum/pronotum length ratio 0.58–0.81, rostrum length/width ratio 1.11–1.31. Pronotum length/width ratio 0.82–1.00. Profemur/pronotum length ratio 1.01–1.21, protibia/profemur length ratio 0.86–0.91. Elytra length/width ratio 2.90–3.30.

*Elytra.* Elytral declivity slightly more angulate, forming a 70° angle to main body axis, but otherwise as female.

*Abdomen.* Sternum VII slightly more broadly arcuate posteriorly, 1/2 as long as wide. Tergum VII with posterior margin straight. Pygidium (tergum VIII) with posterior 1/2 punctate; anterior 1/2 rugose.

*Sternum VIII.* Consisting of 2 sub-contiguous, sub-triangular sclerites; posterior margins widely angulate. These projections located on lateral 2/5s of posterior margin, as long as sub-quadrate portion of lamina. Mesal 1/5 with a short, sub-trapezoidal projection.

*Aedeagus.* Length/width ratio 3.07–4.62; lateral margins gently converging posteriorly, abruptly more strongly converging posteriad of ostium. Width of pedon in lateral view becoming gradually narrower posteriorly in anterior 5/6, ventral margins in posterior 1/6 curved sharply outwardly to form a lobe, then abruptly curving to meet dorsal margins at a sharp apical point. Ostium elongate, basally open (sclerotized pedon not baso-ventrally entire), laterally emarginate, apical edge with a recurved invagination. Flagellum apically with a large, narrowly elongate, tortuous, ampullate sclerite, sclerite anteriorly gradually widened and recurved to form a small bulb, situated in anterior portion of flagellum.

#### Comments.

This species exhibits some variation in the sculpture of the prothorax, which can be more or less rugose depending on the specimen.

#### Material examined.

Paratypes – 1 female “Green Val, VII 15 Tex/ Brewster Co., J.W. Green/ Paratype, *Minyomerus conicollis* Green. [red]/ J.W. Green Collection/ *Minyomerus conicollis*” (CAS); “Green Val, VII 15 Tex/ Brewster Co., J.W. Green/ Paratype, *Minyomerus conicollis*, Green./ USNM, Paratype, 54992/ *Minyomerus conicollis* Green” (USNM: 1 female). Additional specimens examined: “Tex. 57 mi S Alpine, Brewster Co. 6-6-1970/ Collectors: L&C.W. O’Brien” (CWOB: 2 males); “Tex. 51 mi S Alpine, Brewster Co. 6-6-1970/ Collectors: L&C.W. O’Brien” (CWOB: 2 males); “TEXAS: Rio Grande Vill., Big Bend Nat’l. Park, June 7, 1972, W. E. Clark” (TAMU: 19 females, 27 males); “TEXAS: Rio Grande Villiage, Big Bend Nat’l. Park, 8-VI-1972, W. E. Clark” (TAMU: 1 female); “12 miles nw., Presidio, Texas, June 24, 1968, J. E. Hafernik (TAMU: 1 female)”; “6 miles south of Dell City, Texas, VI-29-1991, Creosote Bush, W.F. Chamberlain” (TAMU: 4 females, 4 males); “TEXAS: Brewster Co., Black Gap Wdlf. Mgt. Ar., VI-5-1994, Coll. E. G. Riley” (TAMU: 1 male); “3 miles north Presidio, Texas, June 17, 1968, J.E. Hafernik” (TAMU: 7 females, 6 males): “3 miles north Presidio, Texas, June 13, 1968, J.E. Hafernik” (TAMU: 5 females, 3 males): “12 mi. s. Marfa, Texas IX-30-66, C. L. Cole” (TAMU: 1 female).

#### Distribution.

This species has been found in the Big Bend region of Texas (USA), and is likely also found in Coahuila and Chihuahua (Mexico) (Fig. 51).

#### Natural history.

Associated with creosote bush (*Larrea tridentata* [DC.] Coville [non-focal]; Zygophyllaceae [non-focal]).

### Minyomerus languidus [JF2015]

Taxon classificationAnimaliaColeopteraCurculionidae

Horn, 1876 sec. Jansen & Franz (2015)

Figs 16 , 17 , 18

== (INT) AND > (OST) Minyomerus languidus Horn, 1876: 18 sec. Horn (1876)
== (INT) AND > (OST) AND = Pseudelissa cinerea Casey, 1888: 274 sec. Casey (1888) (synonymized by Pierce 1909: 359)

#### Diagnosis.

*Minyomerus languidus* [JF2015] is most readily discerned from other congenerics by a combination of characters. The elytral setae are very uniform, linear, sub-recumbent, and brown, never with interspersed, longer, white setae. The elytral striae are often well defined and impressed, occasionally with punctures evident. The anterior margin of the pronotum has a reduced tuft of ocular vibrissae and is lined with linear setae that are inserted as far as 1/2 their length from the anterior margin. The nasal plate is bounded by a broad, somewhat weakly impressed sulcus. Importantly, the lateral margins of the oral cavity are strongly rounded, never straight, and longer than the posterior margin. Furthermore, the spermatheca has the ramus unconstricted and slightly bulbous.

#### Redescription – female.

*Habitus.* Length 3.28–3.91 mm, width 1.33–1.63 mm, length/width ratio 2.39–2.56, widest at anterior 1/3 of elytra. Integument orange-brown to black. Scales with variously interspersed colors ranging from slightly off-white to manila/tan to dark coffee brown, in some specimens appearing semi-translucent (in others opaque). Setae sub-recumbent.

*Mandibles.* Covered opalescent scales, with 2 longer setae, and 1 shorter seta between these.

*Maxillae.* Cardo bifurcate at base with an inner angle of ca. 100°, inner (mesal) arm 2 × longer than outer arm, inner arm of equal width to outer arm, inner arm of bifurcation equal in length to apically outcurved arm, glabrous. Stipes sub-quadrate, 1.5–2 × longer than wide, roughly equal in length to inner arm of bifurcation of cardo, glabrous. Galeo-lacinial complex apically incurved (mesally); setose in basal 1/2; dorsally with 6 apicomesal lacinial teeth; ventrally with 3 reduced lacinial teeth. Palpiger with a transverse row of 3 setae, sclerotized on basal 2/3.

*Maxillary palps*. Palpomeres I and II both apically oblique, forming a 45° angle with base, I and II each with 2 apical setae.

*Labium.* Prementum roughly pentagonal; apical margins sinuate, strongly angulate; lateral margins incurved; basal margin arcuate. Labial palps 3-segmented, I with apical 1/2 projecting beyond margin of prementum, reaching apex of ligula; III slightly longer than II.

*Rostrum.* Length 0.50–0.64 mm, anterior portion 1.5–2 × broader than long, rostrum/pronotum length ratio 0.63–0.72, rostrum length/width ratio 1.37–1.43. Dorsal outline of rostrum sub-rectangular, anterior half of dorsal surface strongly impressed, posterior half strongly rugose. Rostrum in lateral view rectangular; apical margin with 2 large vibrissae. Nasal plate strongly defined by Y-shaped, impressed lines, convex, covered wth whitish scales. Margins of mandibular incision directed 30° outward dorsally in frontal view; ventrolateral sulci usually defined, beginning as a sulcus dorsad of insertion point of mandibles, running parallel to scrobe, becoming fainter posteriorly and disappearing ventrally. Dorsal surface of rostrum with median sulcus running from fovea at posterior end of anterior half rostrum to midpoint of posterior half of rostrum. Rostrum ventrally lacking foveae in line with insertion point of mandibles. Oral cavity with lateral margins strongly curved.

*Antennae.* Dorsal margin of scrobe overhangs slightly (broadly, not forming a sharp tooth) ventrad of anterior margin of eye. Scape just extending to posterior 1/2 of eye. Funicular antennomeres evenly progressing from elongate to broader than long; terminal segment of equal length but wider than preceding segment and lacking appressed scales, having instead a covering of apically-directed pubescence with interspersed sub-erect setae. Club nearly 2.5 × as long as wide, with setation and pubescence as in final segment of funicle.

*Head.* Eyes separated in dorsal view by 4–5 × their anterior-posterior length, touching anterior prothoracic margin.

*Pronotum.* Length/width ratio 0.80–0.93, sub-cylindrical to conical; widest near anterior 1/4. Anterior margin arcuate, lateral margins feebly curved and widening into a slight bulge just past anterior 1/4 of pronotum, thence straight to posterior margin, posterior margin straight. Pronotum in lateral view with setae that reach beyond anterior margin; these setae becoming evenly longer laterally, reaching into anterior 1/2 of eye at their maximum length. Anterolateral margin with a reduced tuft of post-ocular vibrissae present, consisting of 5–7 setae, emerging near dorsal 1/2 of eye, becoming gradually, evenly longer ventrally, stopping just beneath ventral margin of eye; vibrissae achieving a maximum length 1/2–3/5 × anterior-posterior length of eye.

*Scutellum.* Hidden in some specimens, narrowly exposed in others (visible area approximately equal to length of appressed scales), margins straight.

*Pleurites.* Metepisternum covered by elytron near posterior 1/5 of metasternum.

*Thoracic sterna.* Mesocoxal cavities separated by distance 1/3–1/2 × width of mesocoxal cavity. Metasternum without apprent transverse sulcus; metacoxal cavities widely separated by 4–5 × their width.

*Legs.* Profemur/pronotum length ratio 0.93–1.03; distal 1/5 of profemur produced ventrally as a nearly semicircular projection covering tibial joint. Protibia/profemur length ratio 0.84–0.93; mucro reduced to a very small laterally projected tooth. Protarsus with tarsomere III equilateral. Metatibial apex with almond shaped convex ity ringed by 8 short, widely separated, spiniform setae.

*Elytra.* Length/width ratio 2.94–3.46; widest at anterior 1/3; anterior margins jointly 1.5–2 × wider than posterior margin of pronotum; lateral margins sub-parallel after anterior 1/4, more strongly rounded and converging in posterior 1/2. Elytra sculpted with a depression at anterior 1/3; posterior declivity angled at nearly 70° to main body axis. Punctures visible, deep and distinct in some specimens, separated by 3–4 × their diameter.

*Abdominal sterna.* Ventrite III elevated and set off from IV along lateral 1/4s of its length. Sternum VII mesally 3/5 × as long as wide; setae darker, longer, and becoming more erect and setiform in mesal 1/2 of posterior 2/5; anterior margin weakly curved.

*Tergum.* Pygidium sub-conical; medial 1/3 of anterior 2/3 of pygidium less sclerotized, with a patch of very short, fine setae.

*Sternum VIII.* Anterior laminar edges of spiculum ventrale each incurved forming a 100° angle with lateral margin; lamina more sclerotized medially.

*Ovipositor.* Coxites in dorsal view 2 × as long as broad; styli 3/4 × length of coxites, with 4 long setae near base.

*Spermatheca.* Comma-shaped; collum short, apically with hood-shaped projection sub-parallel to ramus, 2/3 × length of ramus and contiously aligned with curvature of bulb of ramus; collum sub-contiguous with, and angled at 90° to ramus; ramus bulbous, slightly larger than collum; corpus not swollen, of equal thickness to collum and cornu; cornu elongate, apically gradually narrowed, strongly recurved in basal 1/4, gently curved along mesal 1/2, and curved and somewhat flattened near apical 1/4 such that apex is sub-parallel to hood-shaped projection of collum.

**Figure 16.**
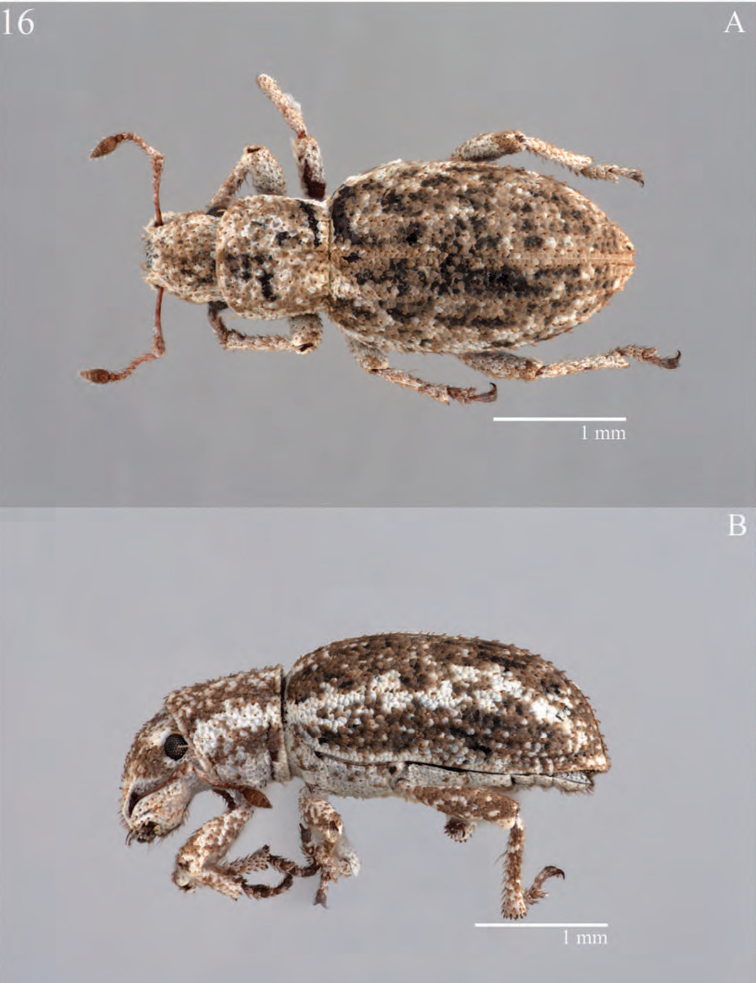
Habitus of *Minyomerus languidus* [JF2015], female **A** lateral view **B** dorsal view.

**Figure 17.**
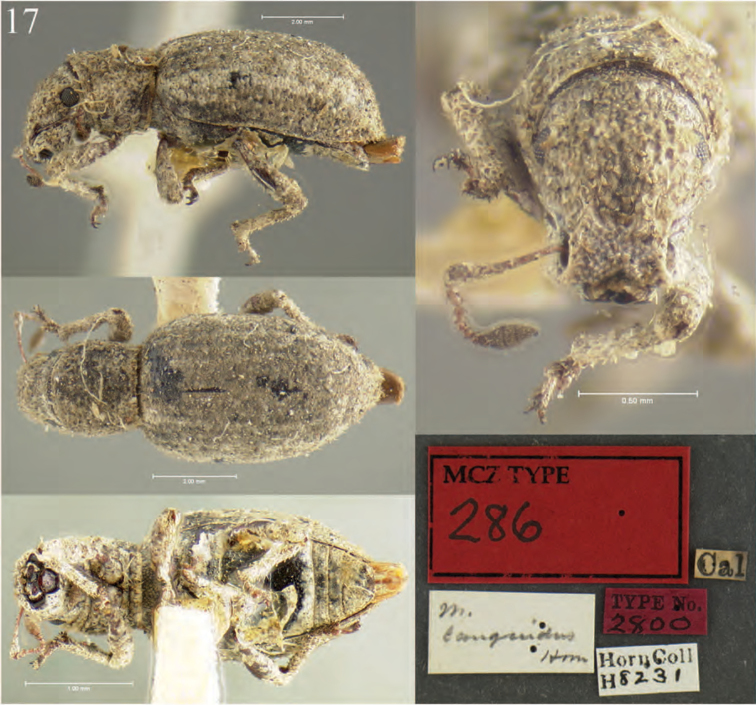
Habitus of female holotype of *Minyomerus languidus* [JF2015], located in the Harvard University Museum of Comparative Zoology, Cambridge, Massachusetts, USA. Images courtesy of MCZ Type Database, http://insects.oeb.harvard.edu/mcz/Species_record.php?id=286

**Figure 18.**
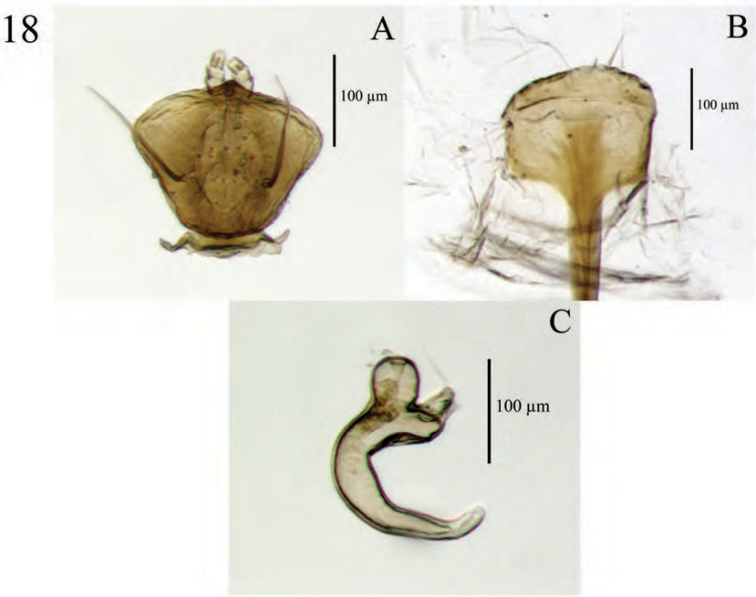
Diagnostic features and terminalia of *Minyomerus languidus* [JF2015] **A** labial prementum, ventral view **B** lamina of sternum VIII **C** spermatheca.

#### Male.

Not available or known.

#### Material examined.

Holotype (for *Minyomerus languidus* sec. Horn [1876]) – female (only images seen: MCZ Type Database) “Cal/ Type No. 2800 [old ANSP number, red]/ Horn Coll H 8231/ *Minyomerus languidus* Horn/ MCZ TYPE 286 [red]” (MCZ); holotype (for *Pseudelissa cinerea* sec. Casey [1888]; junior synonym) “●Tex [Casey locality code: El Paso including both banks of the Riv. Grande which is here about 100 ft. in width]/ Casey bequest 1925/ Type USNM 35007 [red]/ *Pseudelissa cinerea* Cas” (USNM: 1 female). Additional specimens examined: “PORTAL, Ariz, 1 mi. E. i 6-VII-59, D.G. Kissinger, Acc. No. 510/ *Minyomerus languidus*, Horn, det. D.G. Kissinger” (CSCA: 5 females); “TEX. Briscoe Co. 27 mi. NE. Silverton, Hwy 70, V-14-1970/ Collector: C.W. O’Brien” (CWOB: 8 females) [4 females deposited at CMNC]; “AZ. Cochise Co, Hwy 186, 3 mi SE. Wilcox, on Blue Sky Road, 4200’, Sept. 5, 2007/ sand dunes, LB. & C.W. O’Brien, on *Atriplex* [non-focal]” (CWOB: 3 females); “Ariz. 7 mi. S. Pearce 4150, Cochise Co., IX-7-1965/ on Astera[c]ea[e?] [non-focal], C.W. O’Brien” (CWOB: 4 females); “Tex. Pine Springs, Culberson Co., night 8-18-1970, C.W. O’Brien” (CWOB: 4 females); “Tex. Pine Springs, Culberson Co., night 8-18-1970, L.B. O’Brien” (CWOB: 13 females) [6 females deposited at CMNC]; “Socorro Co. NM, Sevilleta NWR, LTER Site 6, 11 Mar 1992/ 34 [series ID]” (CWOB: 1 female); “Socorro Co. NM, Sevilleta NWR, LTER Site 6, 27 May 1992/ 42 [series ID, part of series including 42, 43, 45, and 46]” (CWOB: 4 females).

#### Distribution.

This species has been found in the desert and arid regions of east-central California, Nevada, Arizona, New Mexico, Texas (USA); O’Brien and Wibmer (1982: 46) also have Baja California listed, but this might be a result of a misidentification and confusion with *Minyomerus gravivultus* [JF2015]. It is likely that its range also includes northern Chihuahua, Coahuila, and Sonora (Mexico), based on similarity in habitat to the currently known distribution (Fig. 51).

#### Natural history.

Associated with creosote bush (*Larrea tridentata* [DC.] Coville [non-focal]; Zygophyllaceae [non-focal]), broomweed (*Gutierrezia* [non-focal] sp.; Asteraceae [non-focal]), and saltbush (*Atriplex* [non-focal] sp.; Amaranthaceae [non-focal]). This species is putatively considered parthenogenetic given the lack of male specimens across a range of sampling events.

### Minyomerus microps [JF2015]

Taxon classificationAnimaliaColeopteraCurculionidae

(Say, 1831) sec. Jansen & Franz (2015)

**Figure 19.**
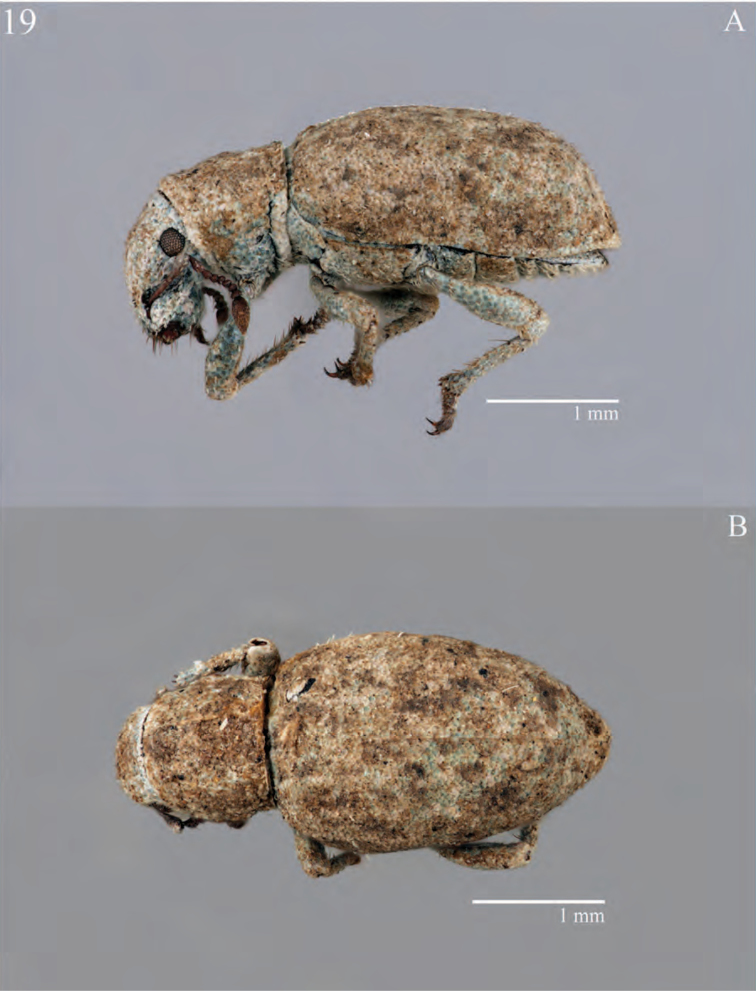
Habitus of *Minyomerus microps* [JF2015], female **A** lateral view **B** dorsal view.

**Figure 20.**
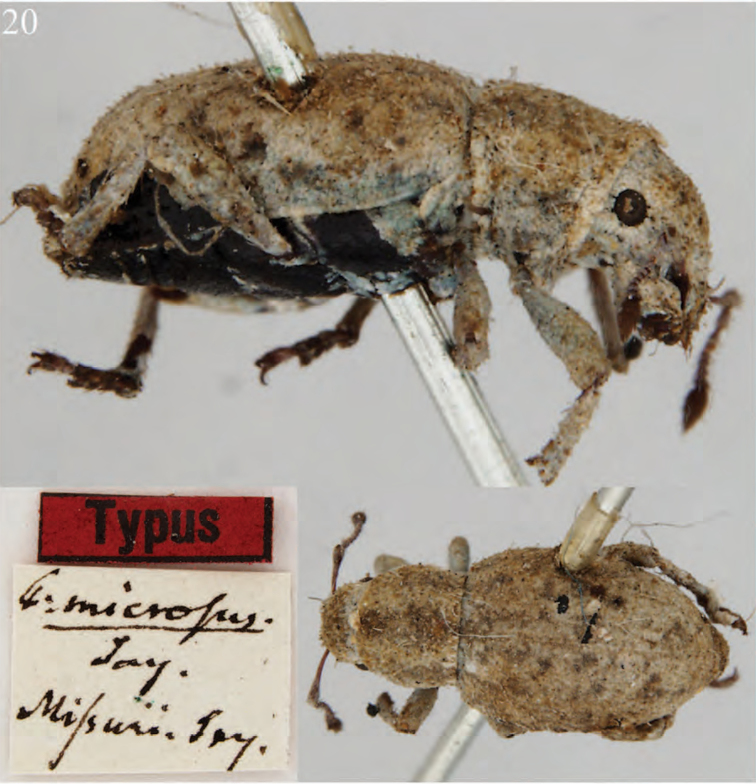
Habitus of female holotype of *Minyomerus microps* [JF2015], located in the Naturhistorika Riksmuseet, Stockholm, Sweden. Images courtesy of Jens Prena.

**Figure 21.**
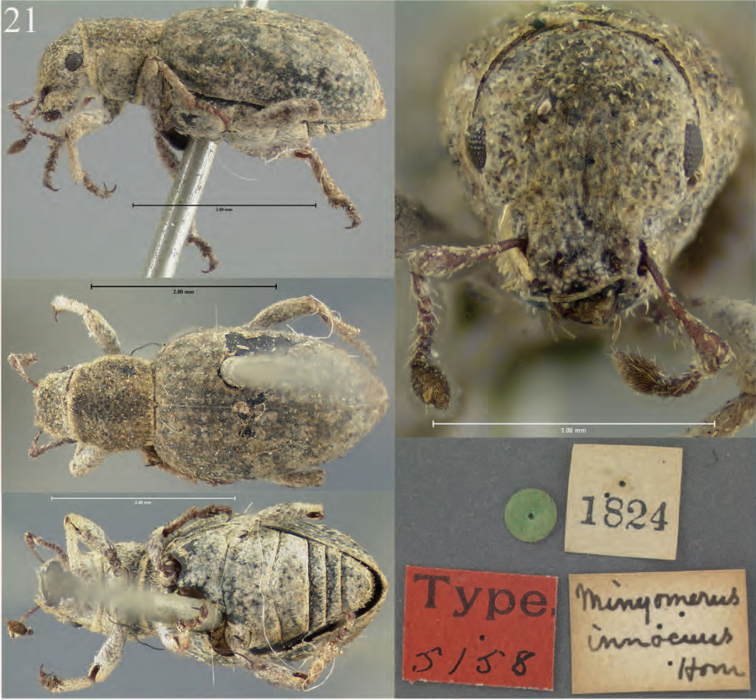
Habitus of female holotype of *Minyomerus innocuus* [JF2015], located in the Harvard University Museum of Comparative Zoology, Cambridge, Massachusetts, USA. Images courtesy of MCZ Type Database, http//insects.oeb.harvard.edu/mcz/Species_record.php?id=4892

**Figure 22.**
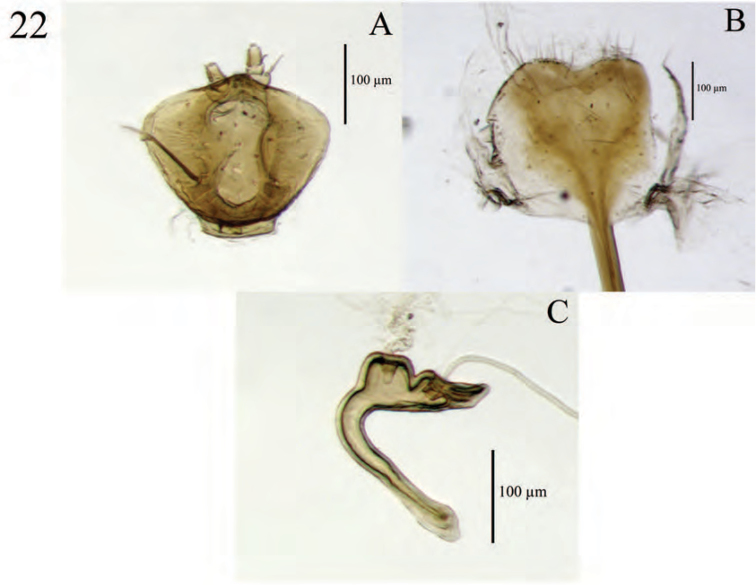
Diagnostic features and terminalia of *Minyomerus microps* [JF2015] **A** labial prementum, ventral view **B** lamina of sternum VIII **C** spermatheca.

Figs 19 , 20 , 21 , 22

== (INT) AND > (OST) AND = Thylacites microps Say, 1831: 9 sec. Say (1831) (transferred to *Minyomerus*Blackwelder and Blackwelder [1948] on the authority of Buchanan *in litt.* by Blackwelder and Blackwelder, 1948: 46)
== (INT) AND > (OST) AND = Thylacites microsus Boheman, 1833: 523 sec. Boheman (1833) (synonymized by LeConte, 1859: 286)
== (INT) AND > (OST) AND = Minyomerus innocuus Horn, 1876: 18 sec. Horn (1876) (formerly recognized type, designated by Pierce, 1913: 400), syn. n.

#### Nomenclatural and taxonomic emendations.

Taxonomic comparison of the type specimen of *Minyomerus microps* and the holotype of *Minyomerus innocuus* reveals that they are conspecific in the context of the present revision. We therefore propose that *Minyomerus innocuus* sec. Horn (1876) be changed to junior synonymy of *Minyomerus microps* [JF2015]. The Discussion further elucidates the historical and present use and status of this species.

#### Diagnosis.

*Minyomerus microps* [JF2015] is best differentiated from other congenerics by a combination of characters. The pronotum is anteriorly constricted, and has a reduced tuft of post-ocular vibrissae present on the anterior margin. The elytral setae are small, subrecumbent, and linear. The scales have a unique optical property that gives them a very ‘crusty’ appearance generally. The elytra and prothorax are often quite bulky compared to other species, and the elytral striae are often roughly and broadly sculpted, but not punctate. The spematheca is also very distinctive in having the ramus and collum appearing as two subcontiguous apically invaginated bulbs.

#### Redescription – female.

*Habitus*. Length 3.73–4.02 mm, width 1.834–1.557 mm, shape sub-cylindrical to ovate, length/width ratio 2.18–2.40, widest at anterior 1/4 of elytra. Integument dark brown to black. Scales with variously interspersed colors ranging from slightly beige to grey to dark coffee brown, in some specimens appearing semi-translucent (in others opaque) or having blue-green hues, optical effect of scales giving a distinctly ‘crusty’ appearance. Setae sub-recumbent.

*Mandibles.* Covered with elliptical, beige scales, with 2 pairs of longer setae, and a 1 shorter seta between these pairs.

*Maxillae.* Cardo 2 × as long as distance from base of palpomere I to base of palpiger, as wide as palpomere III, bifurcate at base with an inner angle of ca. 135°, inner (mesal) arm 3 × length of outer arm, inner arm as thick as outer arm, inner arm of bifurcation equal in length to apically outcurved arm. Stipes sub-quadrate, slightly longer than wide, roughly equal in length to inner arm of bifurcation of cardo, with 1 lateral seta. Galeo-lacinial complex membranous, with sharp demarcation separating palpiger from galeo-lacinial complex; setose in posterior 2/3; dorsally with 8 apicomesal lacinial teeth; ventrally with 1 reduced lacinial tooth. Palpiger with a transverse row of setae extending laterally just posteriad of palpomere I.

*Maxillary palps*. Three-segmented; I and II both apically oblique, apical ends facing mesally and forming a 45° angle with base, I and II each with 2 apical setae; II with 1 mesoventral seta in addition to 2 apical setae.

*Labium.* Prementum roughly pentagonal; apical margin nearly straight, angulate; each basolateral region with 1 long seta. Labial palps 3-segmented, I with apical 1/2 projecting beyond margin of prementum and reaching apex of ligula; both with 1 apical seta; III longer than II.

*Rostrum.* Length 0.46–0.57 mm, anterior portion 1.5–2 × broader than long, rostrum/pronotum length ratio 0.49–0.56, rostrum length/width ratio 1.02–1.26; shape in cross section rectangular to square. Dorsal outline of rostrum sub-rectangular, posterior half of dorsal surface strongly rugose. Rostrum in lateral view sub-rectangular; basal half of dorsolateral margins very strongly converging anteriorly; apical margin with 2 large vibrissae. Nasal plate defined (sometimes weakly) by Y-shaped, impressed lines, slightly convex, weakly elevated from impressed lines, integument posteriorly covered with scales like those of rest of body, anteriorly with elliptical, white to beige colored scales. Margins of mandibular incision ventrally slightly curved, directed 30° outward dorsally in frontal view. Ventrolateral sulci usually weakly defined (in some specimens entirely obscure) except for endpoints, beginning as a notch dorsad of insertion point of mandibles, continuing parallel to scrobe, and terminating in a fovea ventrad of anterior margin of eye. Dorsal surface of rostrum with short median sulcus running from fovea at posterior end of rostrum, nearly 1/2 × median length of nasal plate. Oral cavity with lateral margins feebly curved.

*Antennae.* Minute tooth formed by overhanging dorsal margin of scrobe anterior to margin of eye by 1/3 of length of eye. Club 3–3.5 × as long as wide.

*Head.* Eyes separated in dorsal view by 4–5 × their anterior-posterior length, set off from anterior prothoracic margin by 1/3 of their anterior-posterior length. Head between eyes rugose and slightly bulging, appearing nearly flat in some specimens.

*Pronotum.* Overall slightly wider than long, length/width ratio 0.80–0.91, sub-cylindrical to slightly globular; median sulcus absent. Anterior margin curved, posterior margin nearly straight. Pronotum in lateral view with setae that reach midpoint between base of seta and anterior margin. Anterolateral margin with a reduced tuft of 3–4 ocular vibrissae present, emerging near ventral 1/2 of eye, becoming longer ventrally, stopping just above ventral margin of eye; vibrissae achieving a maximum length nearly 3/5 × anterior-posterior length of eye.

*Scutellum.* Not or only very minutely exposed.

*Pleurites.* Metepisternum covered by elytron near posterior 1/2 of metasternum; metepimeron entirely covered by elytron.

*Thoracic sterna.* Mesocoxal cavities separated by distance 1/4–1/3 × width of mesocoxal cavity. Metasternum without apparent transverse sulcus; metacoxal cavities widely separated by 3–4 × their width.

*Legs.* Profemur/pronotum length ratio 0.88–0.97; profemur with distal 1/5 produced ventrally as a short, sub-rectangular projection covering tibial joint. Protibia/profemur length ratio 0.86–0.90; mucro reduced to a very small laterally projected tooth. Metatibial apex with almond shaped convex ity ringed by 10 short, widely separated, spiniform setae.

*Elytra.* Length/width ratio 2.58–2.86; widest at anterior 1/4; anterior margins jointly 1.5–1.75 × wider than posterior margin of pronotum, curved posteriorly; humeri broadly rounded, not strongly projected; lateral margins gently curving after anterior 1/4, more strongly rounded and converging in posterior 1/2. Elytra in lateral view broadly convex, in some specimens planar; posterior declivity angled at nearly 55° to main body axis. Elytral striae usually broadly sculpted, occasionally appearing shallow, punctate; punctures faint beneath appressed scales, separated by 6–8 × their diameter; intervals elevated (more or less depending on specimen); scales overall generally appearing grey and drably colored.

*Abdominal sterna.* Ventrite III with posterior 3/4 abruptly elevated, posterior margin inconspicuous mesally, III and IV folding into a sulcus along lateral 1/3s of their length. Sternum VII mesally 1/2 × as long as wide, sub-trapezoidal; setae lengthening, and becoming more erect and setiform in posterior 1/3; anterior margin straight; posterior margin nearly straight, emarginate, and rimmed with short, posteriorly directed setae.

*Tergum.* Pygidium (tergum VIII) sub-cylindrical.

*Sternum VIII.* Anterior laminar edges each incurved forming a 135° angle with lateral margin; more sclerotized medially; posterior edge mesally incurved.

*Ovipositor.* Coxites 1.5 × longer than wide; styli 1/3 × maximum length of coxites, with 2-3 long setae near base.

*Spermatheca.* “U”-shaped; collum long, 1/2 × length of cornu, thickening posteriorly to attain a width of 1/2–3/4 × length of ramus, thence constricted to base of ramus, appearing (from “above”) like a wide bowl, collum sub-contiguous with, and angled at 90° to ramus; ramus bulbous and short, 1/2 × length of collum; corpus not swollen, of equal thickness to collum and cornu; cornu elongate, narrowed slightly apically, roughly equal to joint length of collum and corpus, inner angle between cornu and collum nearly 50°.

#### Male.

Not available or known.

#### Comments.

Members of this species exhibit varying degrees of elevation of the elytral intervals and sculpture of the pronotum.

#### Material examined.

Holotype (for *Thylacites microps* sec. Say [1831]) – female (only images seen; Fig. 20) “*Curculio microsus*., Say., Missuri. Say./ typus [red]” (NHRS); holotype (for *Minyomerus innocuus* sec. Horn [1876]; junior synonym) – female (only images seen; Fig. 21) “[Pale green disk: United States – Nebraska, Kansas, North Dakota, South Dakota, Oklahoma, Colorado, Wyoming, Montana]/ 1824/ *Minyomerus innocuus* Horn/ Type 5158” (MCZ). Additional specimens examined: “Havre. Mon., Wickham, June 10-11/ compared with type, *Minyomerus innocuus* [this label appears on CWOB specimens]” (BYU: 1 female; CAS: 5 females; CWOB: 2 females); “CAN. Alta., 0.5 mi. W. Bindloss, VI-20-1967/ sagebrush, at night, A.G. Raske” (CWOB: 3 females); “Medicine Hat, Alta., 9-VI-1928/ F.S. Carr, Collector” (CAS: 2 females); “Medicine Hat, Alta., F.S. Carr” (BYU: 2 females); “Medicine Hat, Alb-5-28” (BYU: 3 females); “ALBERTA, Hwy. 3, Purple Springs, 2000’, VI-18-1962/ compared with type, *Minyomerus innocuus*/ *Minyomerus innocuus* Horn, Det. C.W. O’Brien, 1965” (CWOB: 1 female); “Chugwater, Wyo, 1955-VI-22/ 207/ *Minyomerus innocuus*, Det. C.W. O’Brien 1971” (CWOB: 1 female); “WY, Natrona Co., Sweetwater River, Hwy 220 nr. Independence Rock/ 5900’, 42°29.6'N, 107°02.8'W, 2-vi-2012, S.M. Clark” (CWOB: 1 female); “COLO. Weld Co., 3 mi. N. Rockport, 5794’ VII-22-1971, O’Briens & Marshall” (CWOB: 1 female).

#### Distribution.

This species has been found in the grassland and semi-arid regions of Alberta (Canada), Colorado, Kansas, Montana, New Mexico, and Wyoming (USA). It is likely that its range also includes other Great Plains states, such as North and South Dakota, Nebraska, Oklahoma, northern Texas, and Saskatchewan (Canada), based on similarity in habitat to the currently known distribution (Fig. 51).

#### Natural history.

Associated with sagebrush (*Artemisia* [non-focal] sp.; Asteraceae [non-focal]). This species is putatively considered parthenogenetic, given the lack of male specimens across a range of sampling events.

### Minyomerus aeriballux [JF2015]

Taxon classificationAnimaliaColeopteraCurculionidae

Jansen & Franz sec. Jansen & Franz (2015) sp. n.

http://zoobank.org/99015B2C-51C7-47AF-AEE7-7F171711F46D

Figs 23 , 24

#### Diagnosis.

*Minyomerus aeriballux* [JF2015] is distinct from other congenerics in having irregular rows of setae on the elytra, where the setae do not form regular rows as in most other species. The setae are generally a lighter color, and are arranged in offset rows on the intervals. The elytra are strongly, distinctly punctate. The punctured elytral striae give this species a uniquely ‘pin-striped’ appearance (see Etymology). The pronotum is medially incurved on both the anterior and posterior margins. The head is distinctly conical in appearance, and is curved medially. The metatibiae are apically strongly convex and covered with setae similar in length to the surrounding setae, somewhat translucent, and slightly lamelliform. The spermatheca has the ramus elongate, annulate, and sub-apically situated on the corpus. The aedeagus is broad, and tapers to the apex. The aedeagal flagellum terminates in an apical sclerite that is irregularly sinuate and tortuous, and is nearly as large as the Aedeagal pedon itself.

#### Description – female.

*Habitus.* Length 5.25–6.49 mm, width 2.00–2.41 mm, shape sub-cylindrical to pyriform, length/width ratio 2.50–2.69, widest at posterior 1/2–2/5 of elytra. Integument dark reddish-brown to black. Scales with variously interspersed colors ranging from white to gold, in some specimens appearing semi-translucent (in others opaque) or with reddish or golden opalescent reflections; dorsal patterning fairly stable in this species, having alternating gold and white stripes on prothorax and elytra. Setae white.

*Mandibles.* Covered with white-opalescent scales, with 4–6 longer setae, and 1–3 shorter interspersed setae.

*Maxillae.* Cardo bifurcate at base with an inner angle of ca. 105°, inner (mesal) arm 2–3× longer and 2× thicker than outer arm, inner arm of bifurcation equal in length to apically outcurved arm. Stipes nearly square, equilateral, roughly equal in length to inner arm of bifurcation of cardo. Galeo-lacinial complex membranous and setose in posterior 2/3, sclerotized and somewhat emarginate anteriorly; dorsally with 7 apicomesal lacinial teeth; ventrally with 3 reduced lacinial teeth. Palpiger with a transverse row of setae; anterior 1/2–1/3 membranous, posteriorly sclerotized. Maxillary palps. I with apical end facing mesally and forming a 45° angle with base, I and II each with 2 apical setae; II with 1 mesoventral seta in addition to 2 apical setae.

*Labium.* Prementum roughly pentagonal; apical margins sinuate, angulate, lateral margins broadly curved, posterior margin mesally incurved; each lateral region with 1 long seta. Labial palps 3-segmented, III with apical 1/3 projecting beyond margin of prementum, but not reaching apex of ligula; both with 2 apical setae.

*Rostrum.* Length 0.70–0.84 mm, anterior portion 2–2.5× broader than long, rostrum/pronotum length ratio 0.55–0.59, rostrum length/width ratio 1.16–1.34. Dorsal outline of rostrum sub-conical, posterior half of dorsal surface strongly rugose. Rostrum in lateral view sub-triangular; apical margin with 2 large vibrissae. Nasal plate weakly defined by Y-shaped, impressed lines, slightly convex, integument partially covered with white-opalescent scales. Margins of mandibular incision directed 15° outward dorsally in frontal view. Ventrolateral sulci weakly defined as a broad concavity dorsad of insertion point of mandibles, running parallel to scrobe, becoming flatter posteriorly and disappearing ventrally. Dorsal surface of rostrum with median sulcus running dorsally from fovea at posterior end of rostrum, equal in length to anterior-posterior length of nasal plate. Rostrum ventrally with integument between 2 converging sulci (beginning at corners of oral cavity) slightly elevated. Oral cavity with lateral margins feebly curved.

*Antennae.* Dorsal margin of scrobe overhanging slightly and forming a small tooth, anterior to margin of eye by 2/5 of length of eye. Funicle slightly longer than scape. Club similar in length to funicular antennomeres V-VII, nearly 2.5× as long as wide.

*Head.* Anterodorsal margin of each eye unimpressed, posterior margin very slightly elevated from lateral surface of head; eyes separated in dorsal view by 4× their anterior-posterior length, set off from anterior prothoracic margin by 1/3 of their anterior-posterior length.

*Pronotum.* Length/width ratio 0.83–0.89, sub-cylindrical to globular; widest between midpoint and anterior constriction; surface punctate, though punctures somewhat obscured by scales; median sulcus absent. Anterior margin nearly straight, subtly incurving mesally, lateral margins curved and widening into a bulge just anteriad of midpoint of pronotum, posterior margin incurved. Pronotum with scales forming 2 parenthesis-shaped, whitish stripes dorsally, laterally with a whitish stripe that continues onto elytron; in lateral view with setae that barely reach beyond anterior margin. Anterolateral margin with a greatly reduced tuft of 1–3 ocular vibrissae present, usually not emerging much beyond fringe of appressed scales, sometimes not apparent.

*Scutellum.* Margins straight.

*Pleurites.* Metepisternum covered by elytron near posterior 1/4 of metasternum.

*Thoracic sterna.* Anterior 1/4 of Mesosternum with a few plumose scales; mesocoxal cavities separated by distance 1/4x width of mesocoxal cavity. Metasternum without apparent transverse sulcus; metacoxal cavities separated by 1.5–2× their width.

*Legs.* Tibiae and trochanters of all legs with a single, hair-like, brown seta positioned on mesal surface, approximately 1.5–2× length of adjacent setae. Profemur/pronotum length ratio 0.93–1.00; profemur with distal 1/5 produced ventrally as a semicircular projection covering tibial joint. Protibia/profemur length ratio 0.89–0.95; protibia moderately stout, in cross section sub-circular, apically expanded; protibial apex with ventral setal comb recessed in a broadly concave groove, setal comb unbroken, but becoming thinner and sparser anteriorly; mucro present as a laterally projected tooth equal in length to nearby setae, triangular and equilateral. Protarsus with tarsomeres II and III equilateral; I-III jointly similar in length to V. Metatibial apex with almond shaped convex ity ringed by numerous longer, spiniform setae.

*Elytra.* Length/width ratio 2.94–3.32; widest at posterior 1/2–2/5; anterior margins jointly 1.5–2× wider than posterior margin of pronotum; lateral margins sub-parallel after anterior 1/4, more strongly rounded and converging in posterior 1/2. Posterior declivity angled at nearly 60° to main body axis. Elytral striae deeply and distinctly punctate, appearing pin-striped, 1/4× width of intervals; punctures distinct, separated by 2–3× their diameter; colors variously interspersed, dorsally with a median longitudinal whitish stripe, laterally with a white stripe continuing from pronotum, these stripes more or less defined on some specimens; each interval medially with 1–2 rows of setae.

*Abdominal sterna.* Ventrite III anteriorly concave, posterior margin elevated and set off from IV along lateral 1/4s of its length. Sternum VII mesally 1/2 as long as wide; anterior margin weakly curved.

*Tergum.* Pygidium (tergum VIII) sub-conical, medial 1/2 of anterior 2/3 of pygidium less sclerotized.

*Sternum VIII.* Anterior laminar edges each incurved forming a 115° angle with lateral margin; lamina more sclerotized medially.

*Ovipositor.* Coxites 1/2 as broad as long in dorsal view.

*Spermatheca.* Comma-shaped; collum short, apically with a large, hood-shaped projection perpendicular to ramus, nearly equal in length and contiously aligned with curvature of bulb of ramus; collum short, cylindrical, sub-contiguous with, and angled at 90° to ramus; ramus elongate, somewhat bulbous, 2/3× thickness of corpus and collum; corpus swollen, slightly thicker than collum, 2× thickness of cornu; cornu elongate, apically, gradually narrowed, strongly recurved in basal 1/4, straight thereafter, extending nearly to extent of projection of collum, forming an inner angle of ca. 45° to collum and corpus.

**Figure 23.**
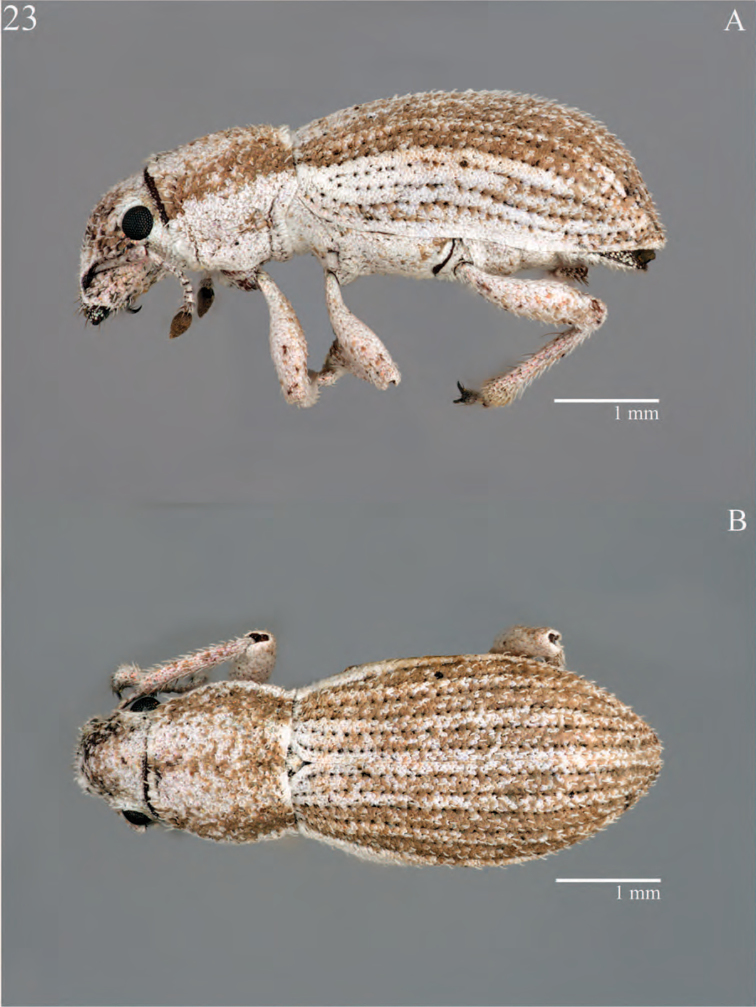
Habitus of *Minyomerus aeriballux* [JF2015], female **A** lateral view **B** dorsal view.

**Figure 24.**
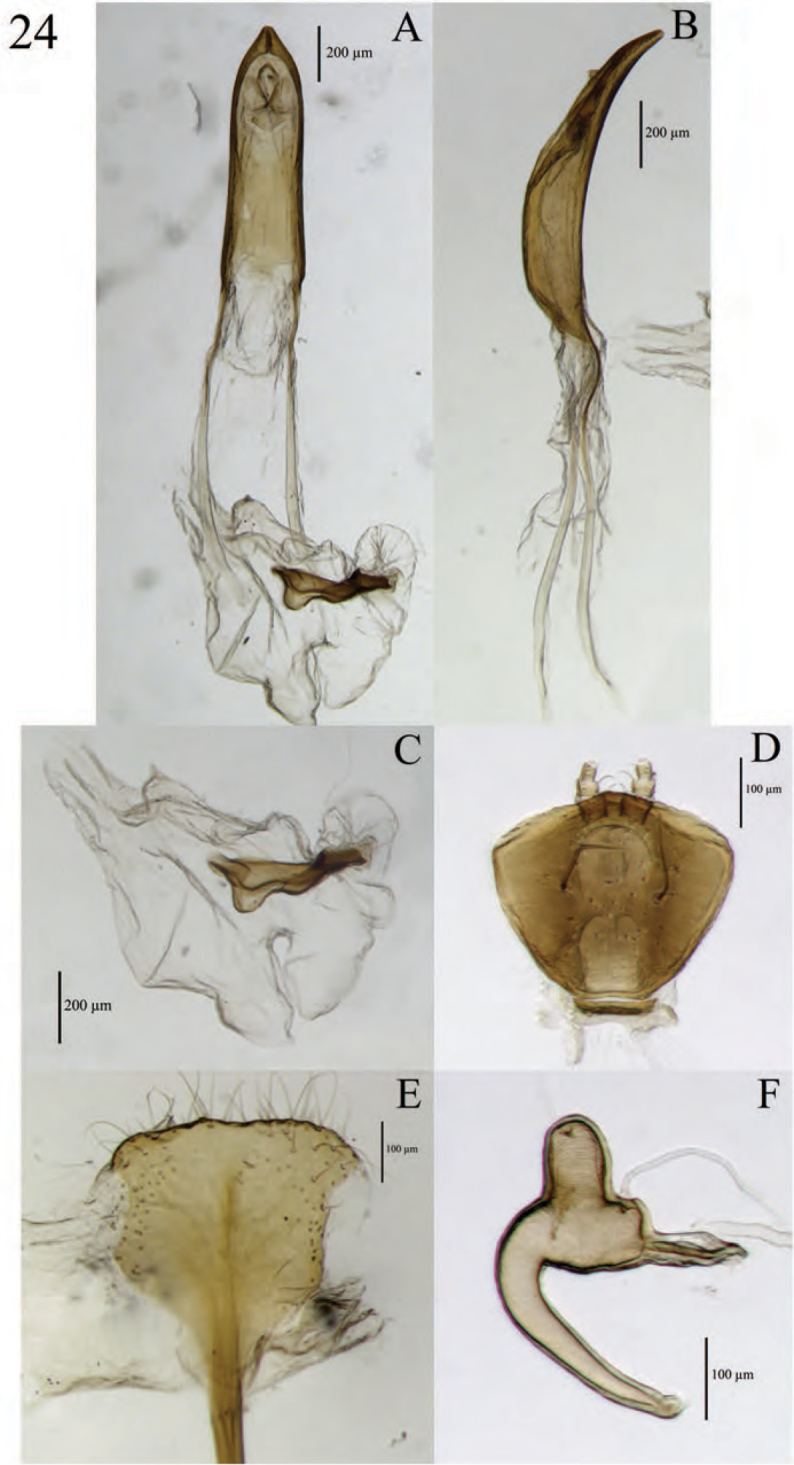
Diagnostic features and terminalia of *Minyomerus aeriballux* [JF2015] **A** aedeagus, dorsal view **B** aedeagus, lateral view **C** apical sclerite of aedeagal flagellum **D** labial prementum, ventral view **E** lamina of sternum VIII **F** spermatheca.

#### Male.

Similar to female, except where noted. Length 4.74–4.97 mm, width 1.65–1.83 mm, length/width ratio 2.66–2.98. Rostrum length 0.63–0.74 mm, rostrum/pronotum length ratio 0.54–0.63, rostrum length/width ratio 1.14–1.41. Pronotum length/width ratio 0.85–0.95. Profemur/pronotum length ratio 0.92–1.03, protibia/profemur length ratio 0.91–0.97. Elytra length/width ratio 3.15–3.43.

*Elytra.* Elytral declivity more angulate, forming a 75° angle to main body axis, but otherwise as female.

*Abdomen.* Sternum VII similar to female. Tergum VII with posterior margin straight. Pygidium (tergum VIII) with posterior 3/4 punctate; anterior 1/4 rugose. Posterior 1/4 constricted and depressed, with posterior margin flaring out and slightly projected dorsally.

*Aedeagus.* Length/width ratio 3.09; lateral margins very slightly converging posteriorly, gradually, more strongly converging in region of ostium. Pedon in lateral view becoming gradually narrower posteriorly in anterior 1/2, ventral margins in posterior 1/2 becoming straight towards apex, then curving to meet dorsal margins at a sharp apical point. Flagellum with large, elonage, tortuous apical sclerite, sclerite nearly as long as pedon, with sinuate margins.

#### Etymology.

Named in reference to the dorsal coloration of the scales and the propensity of living specimens to traverse the open sand habitat in which they can be found, thus appearing as moving pieces of gold in the desert; *aeris* = bronze, brass, or copper, *ballux* = gold-dust or gold-sand; *aeriballux* = bronzy gold-dust; Latin noun in apposition (Brown 1956).

#### Material examined.

Holotype – female “TEX. Winkler Co., 4 mi. N.E. Kermit, X-I-1971, C.W.O’Brien/ on *Artemisia filifolia* [non-focal]” (CWOB). Paratypes, same label information as female holotype (CWOB: 17 females, 19 males) [8 females, 8 males deposited at CMNC]; “USA: NEW MEX: Lea Co., 7 mi. SE Jal, 2980 ft., 32.03275°N, 103.14474°W, VIII-11-2012, E.G. Riley” (TAMU: 1 female); “USA: TEXAS: Winkler Co., Jct. TX hwy 115 & TX FM, 874, 16 rd. km NE Kermit, 31°57’13’’N 102°58’15’’W, X-6-7-2005, J.D. Oswald & Field Ento. Class-393” (TAMU: 1 female); “NEW MEXICO: Eddy Co., 32° 19.8'N, 103° 47.3'W, (Site 7) 23 Sept. 1979, R. R. Murray and J. C. Schaffner, sand dunes” (TAMU: 3 females, 3 males); “NEW MEXICO: Eddy Co., 32° 20.8'N, 103° 46.6'W, (Site 11) 24 April 1979, Burke, Selorme, Schaffner” (TAMU: 1 female).

#### Distribution.

This species has been found in western Texas as well as southeastern New Mexico (Fig. 51).

#### Natural history.

Associated with sagebrush (*Artemisia filifolia* Torrey [non-focal]; Asteraceae [non-focal]).

### Minyomerus reburrus [JF2015]

Taxon classificationAnimaliaColeopteraCurculionidae

Jansen & Franz sec. Jansen & Franz (2015) sp. n.

http://zoobank.org/8D8A2070-17D3-40E5-B757-13B8BC542567

Figs 25 , 26

#### Diagnosis.

*Minyomerus reburrus* [JF2015] is distinct from other congenerics in having irregular rows of copious setae on the elytra, where the setae do not form regular rows as in most other species. The setae are generally a lighter color, and are arranged in offset rows on the intervals, particularly near the elytral suture and declivity. The elytra are somewhat pyriform and weakly punctate. The pronotum is medially incurved on both the anterior and posterior margins. The head is distinctly conical in appearance, and is curved medially. The metatibiae are apically strongly convex and covered with setae similar in length to the surrounding setae, somewhat translucent, and slightly lamelliform. The spermatheca has the ramus elongate, somewhat swollen and sub-apically situated on the corpus.

#### Description – female.

*Habitus.* Length 4.15–4.37 mm, width 1.66–1.83 mm, shape greatly elongate and ovate, length/width ratio 2.39–2.52, widest at anterior 1/3 of elytra. Integument dark brown to black. Scales with variously interspersed colors ranging from white to manila/tan to dark coffee brown, in some specimens appearing semi-translucent (in others opaque) or with bluish or yellowish undertones; dorsal patterning fairly stable in this species, having alternating brown and whitish stripes on prothorax and elytra. Setae short, recumbent, off-white to yellow.

*Mandibles.* Covered with white to yellow scales, with 3 longer setae and 1–2 shorter interspersed setae.

*Maxillae.* Cardo bifurcate at base with an inner angle of 160–170°, inner (mesal) arm 2 × longer than outer arm, inner arm of bifurcation equal in length to apically outcurved arm. Stipes sub-quadrate, 1.5 × wider than long, roughly equal in length to inner arm of bifurcation of cardo, with 1 lateral seta. Galeo-lacinial complexmembranous; setose in posterior 2/3; dorsally with 5 apicomesal lacinial teeth; ventrally with 4 reduced lacinial teeth. Palpiger with a transverse row of setae near anterior 1/3; anterior 1/3 membranous, posterior 2/3 sclerotized.

*Maxillary palps.* Palpomere I with apical end facing mesally and forming a 60° angle with base, I and II each with 2 apical setae.

*Labium.* Prementum roughly pentagonal, convex laterally; apical margins incurved ventrally, straight dorsally, medially projected (ligula), angulate; lateral margins weakly incurved; posterior margin rounded; each lateral region with 1 long seta. Labial palps 3-segmented, I with apexnot projecting beyond margin of prementum, II reaching beyond apexof ligula; I and II both with 1 apical seta; III slightly longer than II, with articulation faint between segments.

*Rostrum.* Length 0.50–0.58 mm, anterior portion 1.5–1.75 × broader than long, narrower than head, rostrum/pronotum length ratio 0.62–0.66, rostrum length/width ratio 1.19–1.37. Dorsal outline of rostrum sub-rectangular, anterior half of dorsal surface strongly impressed. Rostrum in lateral view rectangular; apical margin with 2 groups of 3 large vibrissae, each group inserted just laterad of each sinuation. Nasal plate well defined by V-shaped, impressed lines, mesally planar, integument covered with white scales. Margins of mandibular incision curved, directed 25–30° outward dorsally in frontal view. Ventrolateral sulci defined, beginning as a sulcus dorsad of insertion point of mandibles, running parallel to scrobe, becoming fainter posteriorly and running into a weakly impressed fovea ventrally. Dorsal surface of rostrum with a short, linear, median fovea at posterior end of nasal plate. Rostrum ventrally lacking median fovea and foveae in line with insertion point of mandibles. Oral cavity with lateral margins curved.

*Antennae.* Dorsal margin of scrobe overhanging slightly and forming a small tooth, anterior to margin of eye by 1/3 of length of eye. Club similar in length to funicular antennomeres IV-VII, 2.25–2.5 × as long as wide.

*Head.* Eyes with posterior margin not elevated from lateral surface of head; eyes separated in dorsal view by 3.5–4 × their anterior-posterior length, set off from anterior prothoracic margin by 1/3 of their anterior-posterior length. Head between eyes coarsely, deeply punctate and bulging.

*Pronotum.* Length/width ratio 0.74–0.81, sub-cylindrical to globular; median sulcus absent. Anterior margin incurved mesally, posterior margin incurved mesally. Constricted region elevated and produced dorsally; scales forming 2 parenthesis-shaped, whitish stripes dorsally, laterally with a whitish stripe that continues onto elytron. Pronotum in lateral view with setae extending beyond anterior margin by 1/2 their length. Anterolateral margin with a tuft of post-ocular vibrissae present, emerging near ventral 2/5–1/2 of eye, becoming gradually, evenly longer ventrally, stopping below ventral margin of eye; vibrissae achieving a maximum length 1/2–3/5 × anterior-posterior length of eye.

*Pleurites.* Metepisternum exposed only as a minute triangle anteriorly, covered by elytron posteriorly.

*Thoracic sterna.* Mesosternum with anterior 1/3 incompletely covered by plumose scales, posterior portion as remainder of body surface; mesocoxal cavities separated by distance 1/5 × width of mesocoxal cavity. Metasternum with transverse sulcus apparent; metacoxal cavities separated by 1.5-2.0 × their width.

*Legs.* Profemur/pronotum length ratio 1.03-1.15; profemur with distal 1/5 produced ventrally as a semicircular projection covering tibial joint. Protibia/profemur length ratio 0.94-0.98; protibia in cross section sub-circular; protibial mucro present as a laterally projected tooth. Protarsus with tarsomere II 2/3 × length of III, equilateral, globular; I and II jointly similar in length to V. Metatibial apexwith almond shaped convex ity ringed by 11-13 stout, spiniform setae.

*Elytra.* Length/width ratio 2.69–3.53; widest at anterior 1/3; anterior margins after constriction jointly 2-2.5 × wider than posterior margin of pronotum; lateral margins gently curving after anterior 1/3, more strongly rounded and converging in posterior 1/3. Posterior declivity angled at nearly 60° to main body axis. Elytral striae punctate; punctures clearly visible, separated by 2–4 × their diameter; intervals slightly elevated; dorsally with a median longitudinal whitish stripe, laterally with a white stripe laterally continuing from pronotum.

*Abdominal sterna.* Ventrite III with midregion ventrally concave anteriorly, posterior margin elevated and set off from IV along lateral 1/3 of its length. Sternum VII mesally 1/2 × as long as wide, sub-triangular; anterior margin straight; posterior margin arcuate.

*Tergum.* Pygidium (tergum VIII) sub-cylindrical; medial 1/3 of anterior 2/3 of pygidium less sclerotized, sclerotized regions porose.

*Sternum VIII.* Lamina sub-triangular; lateral edges each incurved forming a 60° angle with spiculum ventrale; sclerotized region porose; posterior margin mesally slightly incurved.

*Ovipositor.* Coxites slightly sclerotized anteriorly, strongly sclerotized in posterior 1/2, 2 × as long as broad; styli 1/3–1/4 × length of coxites, attachment to each coxite nearly straight, with 3-5 long setae near base.

*Spermatheca.* Comma-shaped; collum short, 1/3 × as long as corpus; collum sub-contiguous with, and angled at 90° to ramus; ramus elongate and slightly bulbous, nearly equal in length and width to corpus; corpus not swollen, of equal thickness to collum and ramus; cornu elongate, apically, gradually narrowed, strongly recurved in basal 1/5, sinuate along mesal 2/5, and curved near apical 2/5 such that apexis parallel to collum and corpus, 2–2.5 × joint length of collum and corpus.

**Figure 25.**
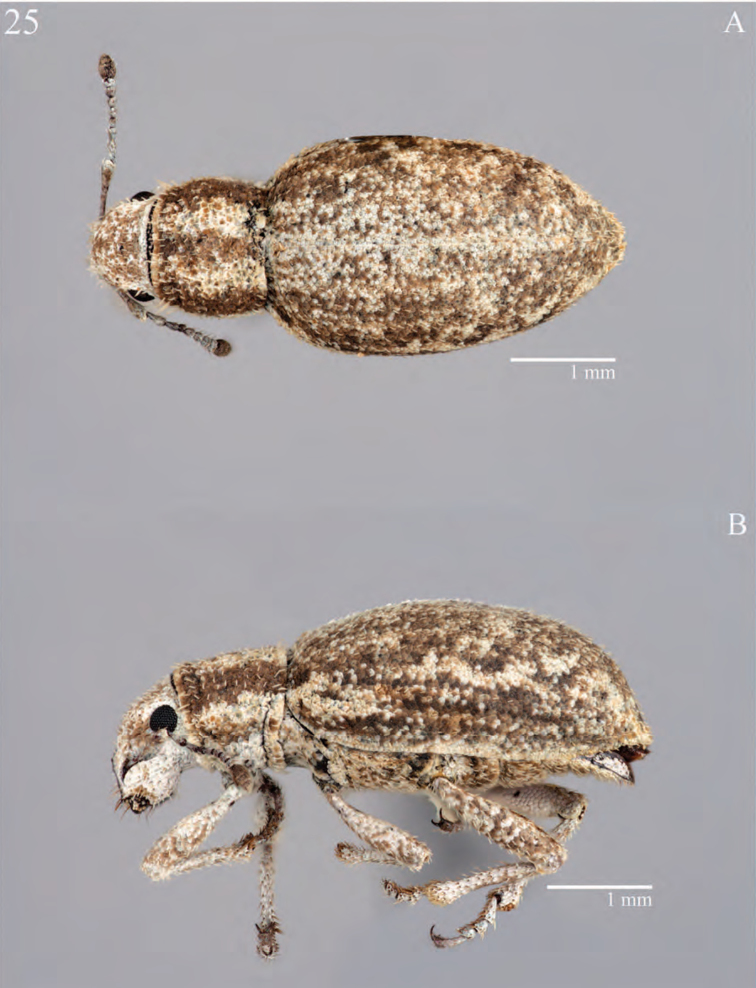
Habitus of *Minyomerus reburrus* [JF2015], female **A** lateral view **B** dorsal view.

**Figure 26.**
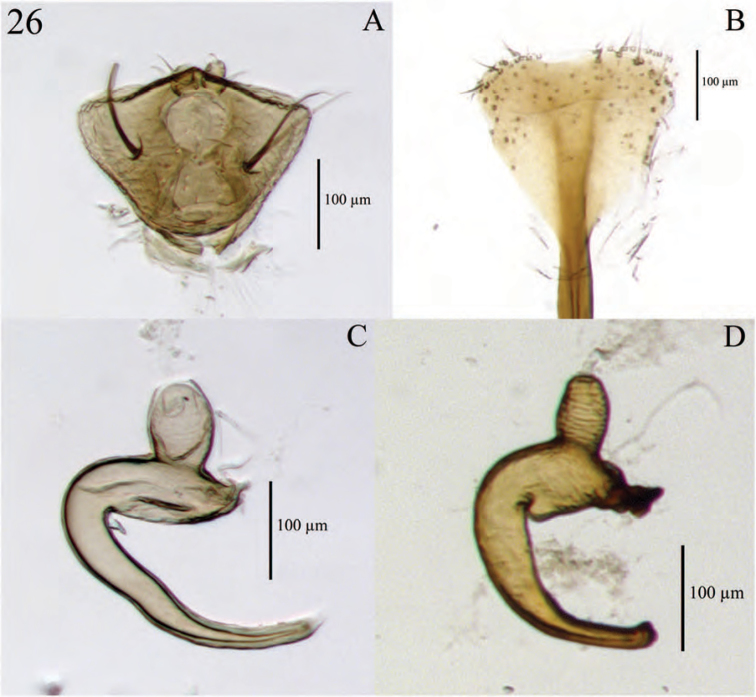
Diagnostic features and terminalia of *Minyomerus reburrus* [JF2015] **A** labial prementum, ventral view **B** lamina of sternum VIII **C** spermatheca **D** spermatheca, variant.

#### Male.

Not available or known.

#### Etymology.

Named in reference to the highly setose aspect of the dorsum; *reburrus* = one with bristling hair; Latin noun in apposition, thence invariable (Brown 1956).

#### Material examined.

Holotype – female “TEX. Bailey Co., 3 1/2 mi. SW. Muleshoe, 7-V-1971, G.B. Marshall/ on *Artemisia* [sic.] [non-focal]” (CWOB). Paratypes, same label information as female holotype (CWOB: 80 females); “NEW MEXICO: Eddy Co., 32°23.2'N, 103°46.6'W, (Site 13) 24 April 1979, Burke, Delorme, Schaffner” (TAMU: 6 females).

#### Distribution.

This species has been found in the desert and arid regions of New Mexico and Texas (USA) (Fig. 51).

#### Natural history.

Associated with sagebrush (*Artemisia* [non-focal] sp.; Asteraceae [non-focal]). This species is putatively considered parthenogenetic, given the lack of male specimens across a range of sampling events.

### Minyomerus cracens [JF2015]

Taxon classificationAnimaliaColeopteraCurculionidae

Jansen & Franz sec. Jansen & Franz (2015) sp. n.

http://zoobank.org/37030793-157B-49D7-B67B-81C302B7DBBB

Figs 27 , 28

#### Diagnosis.

*Minyomerus cracens* [JF2015] is best differentiated from other congenerics by virtue of its elytra, which are each 4–5 × as long as broad in dorsal view. The elytral striae are strongly punctate. The elytra are constricted anteriorly, and narrower than the pronotum, widening thereafter near the humeri. This species also has a somewhat protuberant frons. The spermatheca of this species is unusually shaped, with the corpus somewhat bulbous, and the ramus either flattened somewhat or slightly elongate.

#### Description – female.

*Habitus.* Length 4.29–5.69 mm, width 1.57–1.97 mm, shape greatly elongate and cylindrical, length/width ratio 2.39–2.68, widest at anterior 1/5–1/4 of elytra. Integument dark brown to black. Scales with variously interspersed colors ranging from white to manila/tan to brown, in some specimens appearing semi-translucent (in others opaque) or with a pinkish opalescent sheen. Setae sub-recumbent.

*Mandibles.* Covered with white to opalescent scales, with 4 longer setae, dorsal pair arising from same fovea, and 1–2 shorter interspersed setae.

*Maxillae.* Cardo bifurcate at base with an inner angle of ca. 120°, inner (mesal) arm 2 × thicker and longer than outer arm, inner arm of bifurcation equal in length to apically outcurved arm, glabrous. Stipes sub-quadrate, 1.5 × longer than wide, roughly equal in length to inner arm of bifurcation of cardo. Galeo-lacinial complexsclerotized in apical 1/3, membranous and setose in posterior 2/3; dorsally with 8 apicomesal lacinial teeth; ventrally with 5 reduced lacinial teeth. Palpiger with a transverse row of setae near anterior 1/3; anterior 1/2 membranous, posterior 1/2 sclerotized, mesal and posterior margins emarginate.

*Maxillary palps*. Three-segmented; I with apical end facing mesally and forming a 45° angle with base, I and II each with 2 apical setae; II with 1 mesoventral seta in addition to 2 apical setae.

*Labium.* Prementum roughly trapezoidal, convex laterally; apical margins feebly sinuate, angulate; lateral margins weakly incurved; posterior margin rounded. Labial palps 3-segmented, I with apexprojecting beyond margin of prementum, but not reaching apexof ligula; both with 1 apical seta; III slightly longer than II.

*Rostrum.* Length 0.46–0.73 mm, anterior portion 2–2.5 × broader than long, rostrum/pronotum length ratio 0.41–0.54, rostrum length/width ratio 0.91–1.19. Dorsal outline of rostrum sub-rectangular, anterior half of dorsal surface strongly impressed, posterior half deeply punctate. Rostrum in lateral view rectangular; anterior half of dorsolateral margins diverging somewhat; apical margin with 2 pairs of large vibrissae, each pair inserted just laterad of apexof each sinuation in a single fovea. Nasal plate well defined by V-shaped, impressed lines, slightly convex, integument partially covered with opalescent scales. Margins of mandibular incision directed 35° outward dorsally in frontal view. Ventrolateral sulci deeply and distinctly defined beginning as a notch dorsad of insertion point of mandibles, continuing parallel to scrobe, and terminating in a fovea ventrad of anterior margin of eye, interrupted and appearing as two sulci; sulci on either side of interruption offset from each other such that posterior portion begins ventrad of end of anterior portion. Dorsal surface of rostrum with short, linear, median fovea. Rostrum ventrally lacking foveae in line with insertion point of mandibles. Oral cavity with lateral margins broadly curved.

*Antennae.* Dorsal margin of scrobe overhangs slightly (broadly, not forming a sharp tooth) anterior to margin of eye by 1/4 of length of eye. Club similar in length to funicular antennomeres IV-VII, nearly 2.25 × as long as wide.

*Head.* Eyes separated in dorsal view by 4–5 × their anterior-posterior length, set off from anterior prothoracic margin by 1/3 of their anterior-posterior length. Head between eyes coarsely, deeply punctate and bulging.

*Pronotum.* Length/width ratio 0.98–1.06; widest between midpoint and anterior constriction. Anterior margin arcuate, lateral margins feebly curved and widening into a slight bulge just past midpoint of pronotum, posterior margin straight. Pronotum in lateral view with anterior sulcus continuing dorsally as a series of impressed punctures, anteriorly constricted region elevated and produced dorsally; with setae inserted 2 × their length from anterior margin. Anterolateral margin with a reduced tuft of 8–10 ocular vibrissae present, emerging below ventral margin of eye at a distance of 1/2 × dorsal-ventral length of eye, dorsal half of tuft with short setae, ventral half with setae 2–3 × longer, stopping just below ventral margin of scrobe; vibrissae achieving a maximum length nearly as long as 1/2–3/5 anterior-posterior length of eye.

*Scutellum.* Lateral margins slightly curved outward.

*Pleurites.* Metepisternum covered by elytron near posterior 1/5 of metasternum.

*Thoracic sterna.* Mesocoxal cavities separated by distance 1/4 × width of mesocoxal cavity. Metasternum with transverse sulcus apparent; metacoxal cavities widely separated by 3–4 × their width.

*Legs.* Hind tibiae with numerous longer setae. Profemur/pronotum length ratio 0.80–0.95; profemur with distal 1/5 produced ventrally as a short, semicircular projection covering tibial joint. Protibia/profemur length ratio 0.81–0.90; protibia relatively short and stout, in cross section sub-circular; protibial apexwith ventral setal comb set on curved surface; mucro present as a laterally projected tooth, longer than setal comb and 2 × width of stoutest setae. Protarsus with tarsomere I 1.5–2.0 × as long as II; II 2/3 × length of III, 2–3 × wider than long; II globular; I-III jointly similar in length to V. Metatibial apexwith oblique, almond shaped convex ity ringed by 10 short, widely separated, spiniform setae.

*Elytra.* Length/width ratio 1.74–3.54; widest at anterior 1/5–1/4; anterior 1/10 strongly constricted, narrower than posterior margin of pronotum, and more deeply punctured than remainder of elytra; anterior margins after constriction jointly 1.5–1.75 × wider than posterior margin of pronotum; lateral margins gently curving after anterior 1/4, more strongly rounded and converging in posterior 1/3. Posterior declivity very broadly and evenly arcuate dorsally, ventrally angled at 90° to main body axis. Elytral striae punctate; punctures clearly visible, separated by ca. 2 × their diameter; intervals slightly elevated; each interval medially with a row of sub-recumbent setae.

*Abdominal sterna.* Ventrite III elevated and set off from IV along lateral 1/4 of its length. Sternum VII mesally 1/2–3/5 × as long as wide, sub-trapezoidal; anterior margin weakly curved; posterior margin very broadly arcuate mesally.

*Tergum.* Pygidium (tergum VIII) sub-conical; posterior margin with many long, thin setae inserted along rim; medial 1/4 of anterior 1/2 of pygidium less sclerotized, sclerotized regions porose.

*Sternum VIII.* Lamina sub-quadrate; anterior edges each incurved forming a 90° angle with lateral margin; sclerotized region porose throughout, more sclerotized medially; posterior margin curved.

*Ovipositor.* Coxites slightly sclerotized anteriorly, strongly sclerotized in posterior 1/2, 2 × as long as broad; styli 1/2 × length of coxites, with 3–5 long setae near base.

*Spermatheca.* Comma-shaped; collum as long as corpus, swollen, equilateral; collum sub-contiguous with, and angled at 90° to ramus; ramus with apical edge straight, nearly equal in length to collum, posterior edge tapering off into cornu at a 45° angle, generally rounded in shape; corpus swollen, of equal thickness to collum, 2 × maximum width of cornu; cornu elongate, apically, gradually narrowed, strongly recurved in basal 1/5, nearly perpendicular to corpus along mesal 3/5, sinuate, and curved near apical 1/5 such that apexis parallel to collum and corpus.

**Figure 27.**
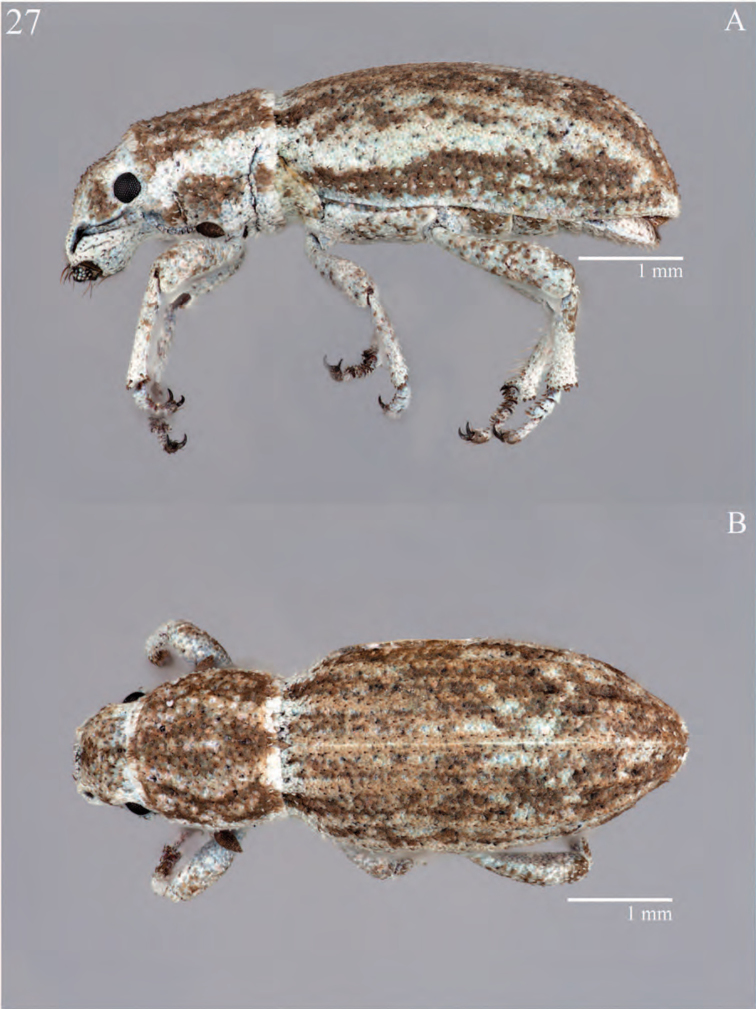
Habitus of *Minyomerus cracens* [JF2015], female **A** lateral view **B** dorsal view.

**Figure 28.**
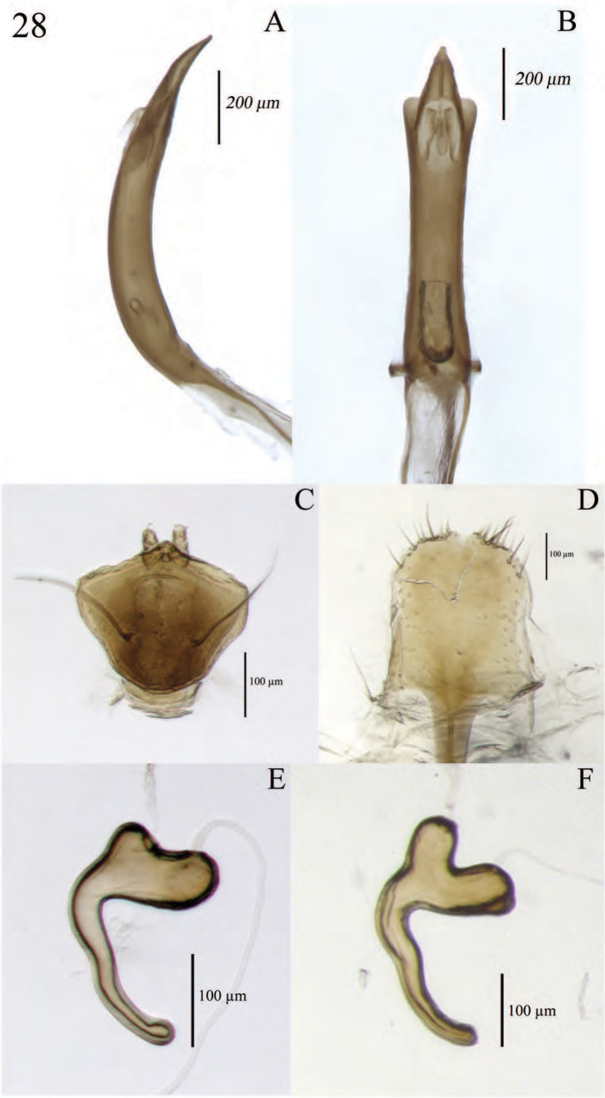
Diagnostic features and terminalia of *Minyomerus cracens* [JF2015] **A** aedeagus, lateral view **B** aedeagus, dorsal view **C** labial prementum, ventral view **D** lamina of sternum VIII **E** spermatheca **F** spermatheca, variant.

#### Male.

Similar to female, except where noted. Length 3.86–4.18 mm, width 1.39–1.56 mm, length/width ratio 2.67–2.85. Rostrum length 0.56–0.62 mm, rostrum/pronotum length ratio 0.44–0.52, rostrum length/width ratio 1.28–1.30. Pronotum length/width ratio 1.04–1.11. Profemur/pronotum length ratio 0.76–0.80, protibia/profemur length ratio 0.76–0.83. Elytra length/width ratio 3.14–3.43.

*Elytra.* Generally shorter, but otherwise as female.

*Abdomen.* Sternum VII slightly more broadly arcuate posteriorly, 1/2–3/5 as long as wide. Pygidium (tergum VIII) with posterior margin with mesal 1/3 straight; posterior 1/3 punctate; anterior 2/3 rugose.

*Aedeagus.* Length/width ratio 4.24–4.70; lateral margins very slightly converging posteriorly, abruptly constricted and more strongly converging in region of ostium. In lateral view, width of pedon becoming gradually narrower posteriorly in anterior 3/4, ventral margins in posterior 1/4 becoming straight towards apex, then abruptly curving to meet dorsal margins at a sharp apical point; apexacutely angulate. Flagellum apically with a small, annuliform sclerite.

#### Etymology.

Named in reference to to the generally slender and elongate appearance of the elytra; *cracens* = slender or graceful; Latin invariable adjective (Brown 1956).

#### Material examined.

Holotype – female “TEX. Gaines, Co. 5 mi. N, Seminole, 22 Aug-1970/ Collector: G.B. Marshall” (CWOB). Paratypes, same label information as female holotype (CWOB: 2 females); “TEX. Bailey Co., Muleshoe, 9-18-1970 night, G.B. Marshall/ *Xanthocephalum sarothrae* [non-focal]” (CWOB: 2 females); “TEX. Bailey Co., Muleshoe, 9-21-1970 night, G.B. Marshall/ *Xanthocephalum sarothrae* [non-focal]” (CWOB: 2 females); “TEX. Bailey Co., Muleshoe, 9-24-1970 night, G.B. Marshall/ *Xanthocephalum sarothrae* [non-focal]” (CWOB: 1 female); “N. Mex. 4 mi., E. Loco Hills, Otero Co., Oct. 4, 1970/ C.W. O’Brien, G. Richardson” (CWOB: 5 females); “N. Mex. Lea, Co. 19 mi. NE., Lovington, Oct. 4, 1970/ G. Richardson, C.W. O’Brien” (CWOB: 9 females); “N. Mex. Lea, Co. 1 mi. W, Lovington, Oct. 4, 1970/ C.W. O’Brien, G. Richardson” (CWOB: 1 female); “N. Mex. Jal, Lea Co., x-11-1970, L. & C.W. O’Brien/ *Xanthocephalum* [non-focal]” (CWOB: 6 females); “Seagraves, TEX. Gaines, Co. x-10-1970, C.W. O’Brien/ *Xanthocephalum* [non-focal]” (CWOB: 25 females) [10 females deposited at CMNC]; “TEX. Bailey Co., Muleshoe 9 30, -1970 night L. &, C.W. O’Brien/ *Xanthocephalum sarothrae* [non-focal]” (CWOB: 21 females); “TEXAS: Pecos Co., Jct. FM 2023 & I-10, 30.86447°N, 102.58662°W, VIII-24-2007, E. G. Riley” (TAMU: 10 males, 13 females).

#### Distribution.

This species has been found in the desert and arid regions of eastern New Mexico and western Texas. It is likely that its range also includes western Oklahoma, based on similarity in habitat to the currently known distribution (Fig. 50).

#### Natural history.

Associated with broomweed (*Gutierrezia* [non-focal] sp., including *Gutierrezia sarothrae* [Pursh] Britt. & Rusby [non-focal]; Asteraceae [non-focal]).

### Minyomerus caseyi [JF2015]

Taxon classificationAnimaliaColeopteraCurculionidae

(Sharp, 1891) sec. Jansen & Franz (2015)

Figs 29 , 30

== AND = Pseudelissa caseyi Sharp, 1891: 151 sec. Sharp (1891) (genus synonymized by Pierce, 1909: 359).

#### Diagnosis.

*Minyomerus caseyi* [JF2015] is readily distinguished from other congenerics by the presence of apically explanate elytral setae. The corpus of the spermatheca is uniquely elongate.

#### Redescription – female.

*Habitus.* Length 3.61–3.96 mm, width 1.52–1.72 mm, length/width ratio 2.31–2.55, widest at anterior 1/4 of elytra. Integument black on tagmata and elytra, light to dark orange-brown on other appendages. Scales with variously interspersed colors ranging from slightly off-white to manila/tan to dark coffee brown, in some specimens appearing to have opalescent reflections. Setae apically explanate, appearing spatulate, sub-recumbent.

*Mandibles.* Covered with white scales, with 4 longer setae, and 1–2 shorter interspersed setae.

*Maxillae.* Cardo 2 × as long as distance from base of palpomere I to base of palpiger, bifurcate at base with an inner angle of ca. 90°, inner (mesal) arm 2 × longer than outer arm, inner arm 1.5 × width of outer arm, inner arm of bifurcation equal in length to apically outcurved arm. Stipes sub-quadrate, slightly wider than long, roughly equal in length to inner bifurcation of cardo, glabrous. Galeo-lacinial complexmembranous and setose in posterior 3/4, sclerotized and somewhat emarginate anteriorly; dorsally with 8 apicomesal lacinial teeth; ventrally with 3 reduced lacinial teeth. Palpiger with a row of transverse setae; membranous in anterior 1/3.

*Maxillary palps.* I and II both apically oblique, apical ends facing mesally and forming a 45° angle with base, I and II each with 2 apical setae; II with 1 mesoventral seta in addition to 2 apical setae.

*Labium.* Prementum completely covering Maxillary palps; roughly trapezoidal, ventrally broadly convex, planar laterally; apical margin laterally incurved, medially projected (ligula) with a distinct anterior face, angulate. Labial palps 3-segmented, I with apical 1/2 projecting beyond margin of prementum, but not reaching apexof ligula; all 3 segments with 1 apical seta; III slightly longer than II.

*Rostrum.* Length 0.41–0.47 mm, anterior portion 2.5–3.0 × broader than long, rostrum/pronotum length ratio 0.43–0.49, rostrum length/width ratio 0.92–0.98. Separation of rostrum from head generally obscure. Dorsal outline of rostrum sub-rectangular, anterior half of dorsal surface impressed, posterior half strongly rugose. Rostrum in lateral view nearly square; apical margin broadly bisinuate, with 2 large vibrissae. Nasal plate defined by V-shaped, impressed lines, anteromesally slightly convex, integument partially covered with white scales. Margins of mandibular incision directed 20° outward dorsally in frontal view. Ventrolateral sulci strongly defined as a deep notch or sulcus dorsad of insertion point of mandibles. Dorsal surface of rostrum with short, linear, median fovea. Rostrum ventrally with median fovea near base of rostrum that continuing very shallowly towards posterior margin of head.

*Antennae.* Small tooth formed by overhanging dorsal margin of scrobe anterior to margin of eye by 1/3 of length of eye. Club similar in length to funicular antennomeres IV-VII, nearly 2.5 × as long as wide.

*Head.* Eyes globular to ovate, posterior margin level with lateral surface of head, slanted antero-ventrally ca. 45°; eyes separated in dorsal view by 4 × their anterior-posterior length, set off from anterior prothoracic margin by 1/5 of their anterior-posterior length.

*Pronotum.* Length/width ratio 0.93–0.98; widest between anterior constriction and midpoint. Anterior margin feebly arcuate, lateral margins feebly curved and widening into a slight bulge just anteriad of midpoint of pronotum, posterior margin straight, with a slight mesal incurvature. Pronotum in lateral view with setae that barely reach anterior margin. Anterolateral margin with a reduced tuft of 3–5 ocular vibrissae present, emerging near ventral 1/2 of eye, becoming gradually, evenly longer ventrally, stopping just below ventral margin of eye; vibrissae achieving a maximum length similar to anterior-posterior length of eye.

*Scutellum.* Minute and not apparent.

*Pleurites.* Metepisternum nearly hidden by elytron except for triangular extension.

*Thoracic sterna.* Prosternal process not elevated. Mesocoxal cavities separated by 1/5 × width of mesocoxal cavity. Metasternum with transverse sulcus apparent but obscured somewhat by scales; metacoxal cavities widely separated by 3 × their width.

*Legs.* Profemur/pronotum length ratio 0.88–0.95; profemur with distal 1/5 produced ventrally as a sub-rectangular projection covering tibial joint; condyle of tibial articulation occupying 2/3 of distal surface and 1/5 length of femur. Protibia/profemur length ratio 0.83–0.91; protibial apexwith ventral setal comb recessed in a subtly incurved groove; mucro reduced to a very small laterally projected tooth. Protarsus with tarsomere III 1.5 × as long as II; wider than long. Metatibial apexwith almond shaped convex ity ringed by 10–12 short, spiniform setae.

*Elytra.* Length/width ratio 2.84–3.00; widest at anterior 1/4; anterior margins jointly 1.5–1.75 × wider than posterior margin of pronotum; lateral margins sub-parallel after anterior 1/4, more strongly rounded and converging in posterior 1/2. Posterior declivity, angled at nearly 75° to main body axis. Elytra with 10 complete striae; striae shallow; punctures faint beneath appressed scales, separated by 4–6 × their diameter; intervals very slightly elevated.

*Abdominal sterna.* Ventrite III anteromesally incurved around a fovea located mesally on anterior margin, posterior margin elevated and set off from IV along lateral 1/4s of its length. Sternum VII mesally 1/2–3/5 × as long as wide; setae darkening, lengthening, and becoming more erect in posterior 1/4; anterior margin weakly curved.

*Tergum.* Pygidium (tergum VIII) sub-cylindrical, posterior margin cultellate, with a few minute setae inserted along rim.

*Sternum VIII.* Anterior 9/10 narrowly stylate; posterior 1/10 (lamina) sub-quadrate; anterior edges each forming a 90° angle with lateral margin; sclerotized region with anterior 1/6 less sclerotized; posterior edge mesally incurved.

*Ovipositor.* Coxites slightly sclerotized, nearly as broad as long; styli 3/4 × length of coxites, glabrous.

*Spermatheca.* “U”-shaped; collum long, equal in length to cornu, sinuate, narrowed to 1/2 × width of base in apical 1/3, collum sub-contiguous with, and angled at 90° to ramus; ramus bulbous, 1/3 × length of collum; corpus not swollen, of equal thickness to collum and cornu; cornu elongate, narrowed to 2/3 × width of base in apical 1/3, strongly recurved in basal 1/3, straight along apical 2/3, inner angle between cornu and collum nearly 55°.

**Figure 29.**
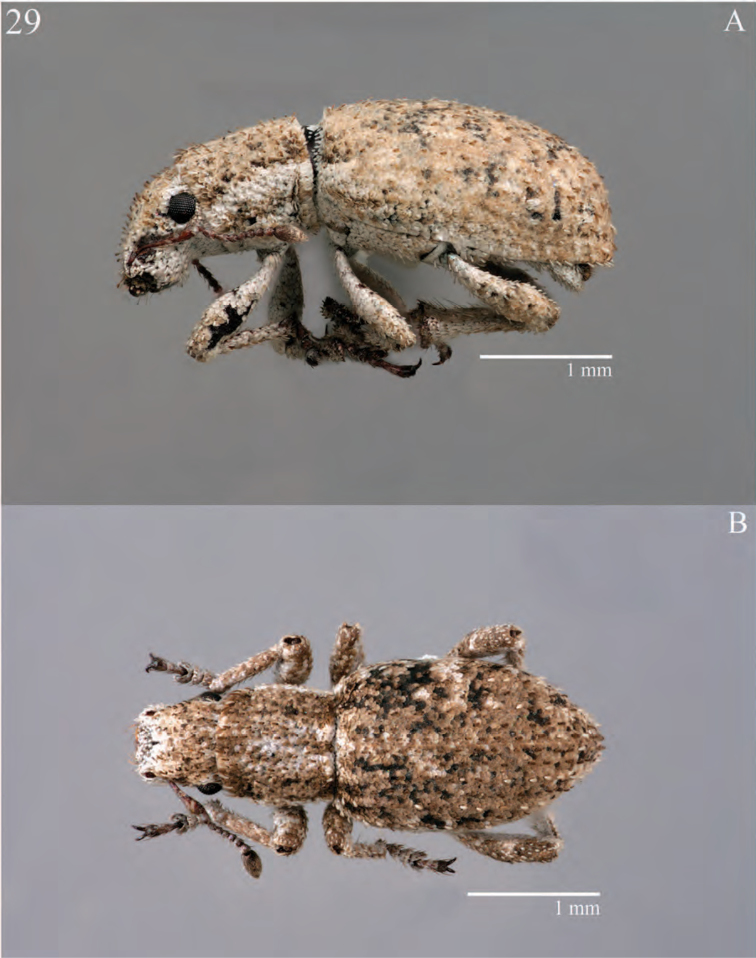
Habitus of *Minyomerus caseyi* [JF2015], female **A** lateral view **B** dorsal view.

**Figure 30.**
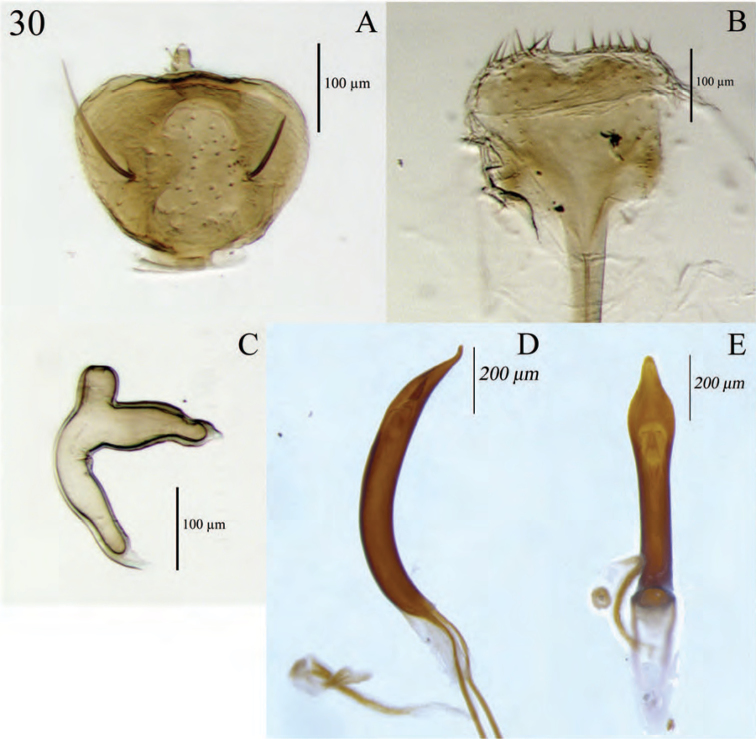
Diagnostic features and terminalia of *Minyomerus caseyi* [JF2015] **A** labial prementum, ventral view **B** spermatheca **C** lamina of sternum VIII **D** aedeagus, lateral view **E** aedeagus, ventral view.

#### Male.

Similar to female, except where noted. Length 3.07–3.64 mm, width 1.18–1.42 mm, length/width ratio 1.98–2.60. Rostrum length 0.41–0.53, rostrum/pronotum length ratio 0.49–0.60, rostrum length/width ratio 1.02–1.30. Pronotum length/width ratio 0.97–1.06. Profemur/pronotum length ratio 0.90–0.96, protibia/profemur length ratio 0.79–0.91. Elytra length/width ratio 2.86–3.37.

*Elytra.* Generally narrower relative to pronotum, but otherwise as female.

*Abdomen.* Sternum VII slightly more broadly arcuate posteriorly, 3/5 as long as wide. Pygidium (tergum VIII) with posterior margin evenly arcuate; posterior 1/2 punctate; anterior 1/2 rugose.

*Aedeagus.* Length/width ratio 5.61–5.84; lateral margins very slightly converging posteriorly, widening in region of ostium, then narrowing more strongly into a small posterior projection. In lateral view, width of pedon even in anterior 3/4, ventral margins in posterior 1/4 becoming straight towards apex, then abruptly curving to meet dorsal margins at a sharp apical point; apexacutely angulate. Flagellum apically with a large, narrowly elongate, micro-denticulate sclerite.

#### Material examined.

“MEXICO, SLP, Hwy 57, 23 mi. SE. San Luis Potosi, 6000’, 10 Sept. 1982 C. & L. O’Brien & G. Wibmer” (CWOB: 6 females, 6 males); “MEX. N, Leon, Hwy 57, 79 mi. SW. Linares, X-16-1970/ at night, L & C.W. O’Brien” (CWOB: 1 female); “MEXICO: Vera Cruz, 10 mi. sw. Perote, July 27, 1974, Clark, Murray, Ashe, Schaffner” (TAMU: 1 female).

#### Distribution.

This species has been found in San Luis Potosí and Nuevo León (Mexico). It is likely to be found throughout the Chihuahuan Desert and arid regions of south-central Mexico based on habitat similarity (Fig. 50).

#### Natural history.

No host plant associations have been documented.

### Minyomerus trisetosus [JF2015]

Taxon classificationAnimaliaColeopteraCurculionidae

Jansen & Franz sec. Jansen & Franz (2015) sp. n.

http://zoobank.org/FD032919-862C-44B8-AB1F-E1305BC87379

Figs 31 , 32

#### Diagnosis.

*Minyomerus trisetosus* [JF2015] can be differentiated from other congenerics by the presence of long, erect, white setae interspersed among the regular rows of shorter, sub-recumbent, brown setae. Additionaly the procoxae are apparently contiguous, and the margins of the oral cavity are straight and slightly divergent. The anterior margin of the pronotum bears a reduced tuft of post-ocular vibrissae as well as a row of setae inserted 1/2 their length from the anterior margin. The spermatheca has the ramus basally constricted.

#### Description – female.

*Habitus.* Length 3.01–3.87 mm, width 1.24–1.70 mm, length/width ratio 2.28–2.44, widest at anterior 1/4–1/3 of elytra. Integument black on tagmata and elytra, light to dark orange-brown on other appendages. Scales with variously interspersed colors ranging from white to manila/tan to dark coffee brown, occasionally opalescent or with undertones of red or yellow, in some specimens appearing semi-translucent (in others opaque). Three kinds of linear setiform scales (‘setae’) sparse throughout; first kind short, erect to sub-erect, brown, arranged in rows on elytral intervals, present on dorsum of thorax and head; second sparser and up to 4 × length and ca. 2 × girth of former, longer and thicker on lateral surfaces, erect to sub-erect, white, interspersed among brown setae on dorsum; venter with setae thinner and more setiform than others, of similar length to brown setae, sub-recumbent, translucent white, becoming longer posteriorly and somewhat darker on terminal abdominal segment.

*Mandibles.* Covered with white to golden scales, with 3 longer setae, and 1 shorter seta ventrad of these.

*Maxillae.* Cardo 1.5 × as long as distance from base of palpomere I to base of palpiger, bifurcate at base with an inner angle of ca. 90°, inner (mesal) arm longer than outer arm, inner arm of equal width to outer arm, inner arm of bifurcation 2 × length of apically outcurved arm. Stipes sub-quadrate, 0.5 × longer than wide, roughly equal in width to inner arm of bifurcation of cardo, with 1 lateral seta. Galeo-lacinial complexapically incurved (mesally); complex membranous; setose in basal half; dorsally with 8 apicomesal lacinial teeth; ventrally with 4 reduced lacinial teeth. Palpiger with a 1 lateral seta and 1 mesal seta, sclerotized on basal 2/3.

*Maxillary palps.* I and II both apically oblique, apical ends facing mesally and forming a 45° angle with base, I and II each with 2 apical setae. *Labium.* Prementum roughly trapezoidal; apical margins sinuate, angulate; lateral margins weakly incurved; basal margin arcuate. Labial palps 3-segmented, I with apical 1/2 projecting beyond margin of prementum, reaching apexof ligula; III slightly longer than II.

*Rostrum.* Length 0.38–0.52 mm, anterior portion 2.5–3.0 × broader than long, rostrum/pronotum length ratio 0.46–0.53, rostrum length/width ratio 0.91–1.10. Dorsal outline of rostrum sub-rectangular, anterior half of dorsal surface strongly impressed, posterior half strongly rugose. Rostrum in lateral view rectangular; apical margin with 2 large vibrissae. Nasal plate strongly defined by Y-shaped, impressed lines, convex, integument covered with white scales. Margins of mandibular incision curved, directed 30° outward dorsally in frontal view; ventrolateral sulci usually weakly defined as a somewhat shallow to moderately deep, but broad, impression dorsad of insertion point of mandibles. Dorsal surface of rostrum with median sulcus running from fovea at posterior end of nasal plate to midpoint of posterior half of rostrum. Rostrum ventrally lacking foveae in line with insertion point of mandibles.

*Antennae.* Dorsal margin of scrobe overhangs slightly (broadly, not forming a sharp tooth) ventrad of anterior margin of eye. Funicular antennomeres evenly progressing from elongate to broader than long; terminal funicular segment lacking appressed scales, having instead a covering of apically-directed pubescence with interspersed sub-erect setae. Club nearly 3 × as long as wide.

*Head.* Eyes strongly impressed; eyes separated in dorsal view by 4–5 × their anterior-posterior length, set off from anterior prothoracic margin by 1/3–1/2 of their anterior-posterior length.

*Pronotum.* Length/width ratio 0.84–0.91, sub-cylindrical to globular; widest near anterior 2/5; surface rugoso-punctate, though punctures somewhat obscured by scales. Anterior margin slightly arcuate, lateral margins feebly curved and widening into a bulge near anterior 2/5 of pronotum, thence straight to posterior margin, posterior margin incurved mesally. Pronotum in lateral view with sub-erect to erect setae that reach just beyond anterior margin in lateral view, but are inserted less than half their length from anterior margin; these setae becoming evenly longer laterally, attaining a maximum length 3/5 × anterior-posterior length of eye. Anterolateral margin with a reduced tuft of post-ocular vibrissae present, consisting of 3–5 setae, emerging dorsad ventral margin of eye, becoming longer ventrally, stopping just beneath ventral margin of eye; vibrissae achieving a maximum length 2/5–3/5 × anterior-posterior length of eye.

*Scutellum.* Hidden in some specimens, narrowly exposed in others (visible area less than 2 × length of appressed scales), margins straight.

*Pleurites.* Metepisternum covered by elytron near posterior 1/6 of metasternum.

*Thoracic sterna.* Mesocoxal cavities separated by distance1/3–1/2 × width of mesocoxal cavity. Metasternum without apprent transverse sulcus; metacoxal cavities widely separated by 3–4 × their width.

*Legs.* Profemur/pronotum length ratio 0.87–0.96; proximal 5/6 of profemur gradually widening, then abruptly constricted with distal 1/6 produced ventrally as a short, nearly semicircular projection covering tibial joint; condyle of tibial articulation occupying 1/6 length of femur. Protibia/profemur length ratio 0.84-0.90; protibia with ventral setal comb situated on a curved edge; mucro reduced to a very small laterally projected tooth. Protarsus with tarsomere I slightly longer than II; I and III similar in length, III equilateral. Metatibial apexwith thin, almond shaped convex ity narrowly ringed by 6-8 short, widely separated, spiniform setae.

*Elytra.* Length/width ratio 2.79–3.23; widest at anterior 1/3; anterior margins jointly 1.5–1.75 × wider than posterior margin of pronotum; lateral margins sub-parallel after anterior 1/4, more strongly rounded and converging in posterior 1/2. Elytra in lateral view sculpted with a depression at anterior 1/4; posterior declivity angled at nearly 80° to main body axis. Elytral striae not well defined, punctures not visible beneath scales, separated by 3–4 × their diameter; elytral suture sometimes slightly elevated; each interval medially with a row of erect to sub-erect setae.

*Abdominal sterna.* Ventrite III with posterior margin slightly elevated and set off from IV along lateral 1/3s of its length; anterior margin of V incurved. Sternum VII mesally 1/2 × as long as wide, sub-trapezoidal; anterior margin weakly curved; posterior margin mesally nearly straight, laterally arcuate. *Tergum.* Pygidium (tergum VIII) sub-conical; medial 1/3 of anterior 2/3 of pygidium less sclerotized.

*Sternum VIII.* Lamina sub-rectangular; anterior edges each incurved forming a 90° angle with lateral margin, not produced to a point anteromedially at connection to spiculum ventrale; less sclerotized medially.

*Ovipositor.* Coxites in dorsal view 2 × as long as broad; styli 3/4 × length of coxites, with 3–5 long setae near base.

*Spermatheca.* Comma-shaped; collum short, not readily distinguished, apically with hood-shaped projection sub-parallel to ramus, 2/3 × length of ramus and contiously aligned with curvature of bulb of ramus; collum sub-contiguous with, and angled at 90° to ramus; ramus bulbous, sharply constricted beneath, as long as 1.5 × length of corpus and collum, as wide as length of corpus and collum; corpus not swollen, of equal thickness to cornu; cornu elongate, apically slightly narrowed, strongly recurved in basal 1/4, nearly straight thereafter, forming a nearly 45° angle with corpus and collum.

**Figure 31.**
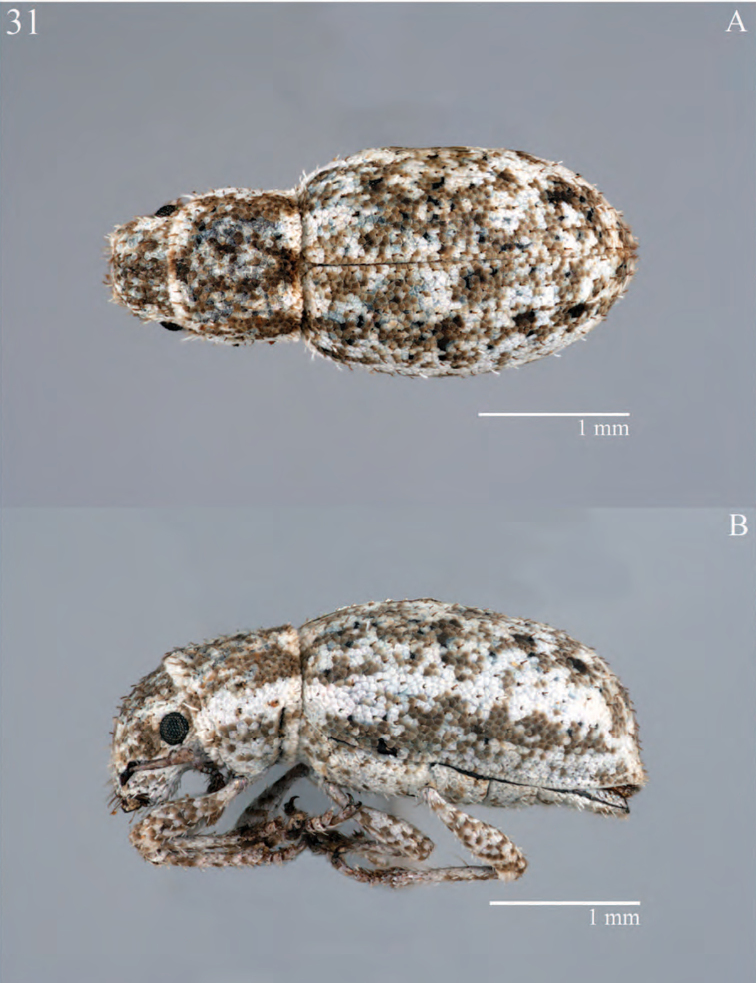
Habitus of *Minyomerus trisetosus* [JF2015], female **A** dorsal view **B** lateral view.

**Figure 32.**
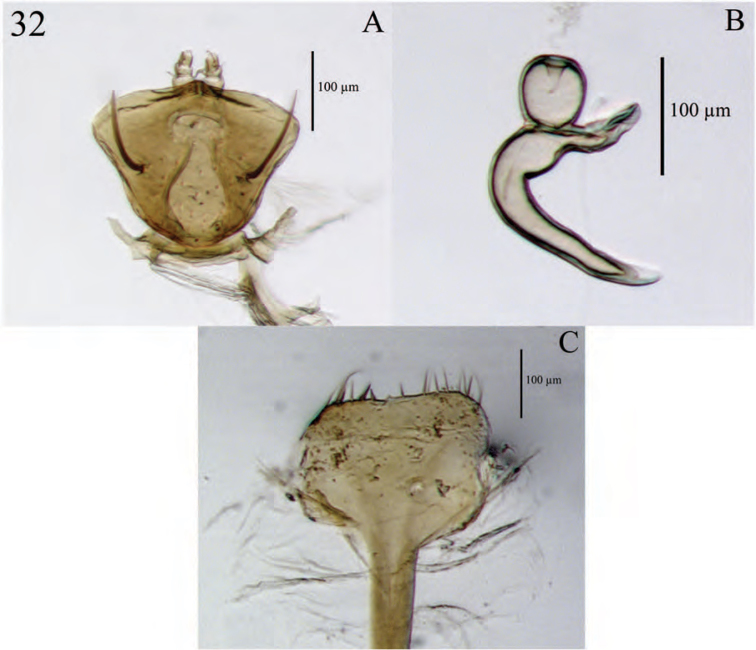
Diagnostic features and terminalia of *Minyomerus trisetosus* [JF2015] **A** labial prementum, ventral view **B** spermatheca **C** lamina of sternum VIII.

#### Male.

Not available or known.

#### Etymology.

Named in reference to the three distinct types of setae present on the body; *tri*- = three, *setosus* = bristly, hence *trisetosus* = with three kinds of bristles; Latin adjective (Brown 1956).

#### Material examined.

Holotype – female “TEX. Lamb Co. 9 mi. W Littlefield, IV-21-1971, C.W. O’Brien” (CWOB). Paratypes, same label information as female holotype (CWOB: 4 females); “TEX. Lubbock, 12-18-1970, pitfall trap, C.W. O’Brien” (CWOB: 6 females); “TEX. Howard Co., 4 mi. S. Big Spring, V-2-1971 night, O’Brien & Marshall” (CWOB: 20 females) [8 females deposited at CMNC]; “TEX. Howard Co., 5 mi. S. Big Spring, V-2-1971 night, O’Brien & Marshall” (CWOB: 9 females); “NM: Lea Co., 19 m Denver City Hwy., 20-X-1980, 2:45 pm, Tony Martin, warm, cloudy, snkweed” (NMSU: 19 females); “TEX. Hangford Co., 8 mi. W. Spearman, VI-3-1971, C.W. O’Brien” (TTUZ: 17 females).

#### Distribution.

This species has been found in the desert and arid regions of New Mexico and Texas (USA). It is likely that its range also includes northern Chihuahua and Coahuila (Mexico), based on similarity in habitat to the currently known distribution (Fig. 50).

#### Natural history.

Associated with broomweed (*Xanthocephalum* [non-focal] sp.; Asteraceae [non-focal]), creosote bush (*Larrea tridentata* (DC.) Coville [non-focal]; Zygophyllaceae [non-focal]), and snakeweed (*Gutierrezia* [non-focal] sp.; Asteraceae [non-focal]). This species is putatively considered parthenogenetic, given the lack of male specimens across a range of sampling events.

### Minyomerus puticulatus [JF2015]

Taxon classificationAnimaliaColeopteraCurculionidae

Jansen & Franz sec. Jansen & Franz (2015) sp. n.

http://zoobank.org/D64AB10B-BE4C-4899-92FB-7F1FB710993D

Figs 33 , 34 , 35

#### Diagnosis.

*Minyomerus puticulatus* [JF2015] is best distinguished from other congenerics by a combination of characters. The elytral striae are usually strongly punctate, with regular rows of brown setae on the intervals. The elytra appear somewhat flattened in lateral view, and do not project far above or below the pronotum. The pronotum has a reduced tuft of post-ocular vibrissae on the anterior margin. Additionally there is a row of setae that are inserted by approximately their own length from the anterior margin, and never less than 3/4 of their length. The margins of the oral cavity are nearly straight, and usually sub-parallel. The nasal plate is strongly impressed and well defined, and the frons is somewhat bulbous. The spermatheca is quite distinct, with the ramus basally tapered, and the corpus possessing an annulate, cylindrical projection nearly 2/3 × length of the ramus. The aedeagus is uniquely narrow and elongate, and bears a very minute apical flagellar sclerite.

#### Description – female.

*Habitus.* Length 3.37–4.19 mm, width 1.33–1.58 mm, length/width ratio 2.53–2.65, widest at anterior 1/3–2/5 of elytra. Integument dark reddish-brown to black. Scales slightly off-white to manila/tan to brown, in some specimens appearing semi-translucent (in others opaque). Setae minute, becoming longer on sides of pronotum and venter.

*Mandibles.* Covered with white to yellowish scales, with 4 longer setae, and 1–2 shorter interspersed setae.

*Maxillae.* Cardo bifurcate at base with an inner angle of ca. 100°, inner (mesal) arm 2 × longer and of equal thickness, inner arm of bifurcation equal in length to apically outcurved arm. Stipes nearly square, equilateral, roughly equal in length to inner arm of bifurcation of cardo, with 1 long lateral seta. Galeo-lacinial complexmembranous and setose in posterior 2/3, sclerotized and somewhat emarginate anteriorly; dorsally with 9 apicomesal lacinial teeth; ventrally with 3 reduced lacinial teeth. Palpiger with a transverse row of setae; anterior 1/5 membranous, posteriorly sclerotized.

*Maxillary palps.* Palpomere 1 with apical end facing mesally and forming a 45° angle with base, I and II each with 2 apical setae; II with 1 mesoventral seta in addition to 2 apical setae.

*Labium.* Prementum quadrangular, ventrally sub-planar throughout; apical margins nearly straight, angulate, lateral margins slightly incurved, posterior margin broadly curved. Labial palps 2-segmented, I with apical 1/3 projecting beyond margin of prementum, but not reaching apex of ligula; I with 1 apical seta; II slightly shorter than I, apically constricted and with sensilla.

*Rostrum.* Length 0.48–0.59 mm, anterior portion ca. 2 × broader than long, rostrum/pronotum length ratio 0.56–0.63, rostrum length/width ratio 1.01–1.19. Dorsal outline of rostrum square, posterior half of dorsal surface strongly rugose. Rostrum in lateral view sub-rectangular; apical margin with 2 large vibrissae. Nasal plate very strongly defined by Y-shaped, impressed lines, convex, integument partially covered with white-opalescent scales. Margins of mandibular incision directed 30° outward dorsally in frontal view. Ventrolateral sulci strongly defined, beginning as a narrow sulcus dorsad of insertion point of mandibles, running parallel to scrobe, terminating in a ventral fovea. Dorsal surface of rostrum with median sulcus running dorsally from fovea at posterior end of nasal plate to midpoint between posterior margins of eyes. Rostrum ventrally lacking foveae in line with insertion point of mandibles. Oral cavity with lateral margins weakly curved.

*Antennae.* Dorsal margin of scrobe overhanging slightly and forming a minute tooth, anterior to margin of eye by 2/5 of length of eye. Terminal funicular segment somewhat oblong in dorsal view, lacking appressed scales, having instead a covering of apically-directed pubescence with interspersed sub-erect setae. Club similar in length to funicular antennomeres IV–VII, 2.25–2.5 × as long as wide.

*Head.* Eyes separated in dorsal view by 4–5 × their anterior-posterior length, set off from anterior prothoracic margin by 2/5 of their anterior-posterior length.

*Pronotum.* Length/width ratio 0.83–0.86, sub-cylindrical to conical; widest near anterior 1/4 just before anterior constriction; surface deeply and coarsely punctate. Anterior margin nearly straight, subtly incurving mesally, lateral margins curved anteriorly and widening into a bulge near anterior 1/4, nearly straight to posterior margin thereafter; anterior constriction subtle in some specimens, posterior margin incurved. Pronotum in lateral view with setae that reach beyond anterior margin; these setae becoming evenly longer laterally, nearly reaching posterior margin of eye at their maximum length. Anterolateral margin with a reduced tuft of post-ocular vibrissae present, consisting of 3–6 setae, usually only 1–2 setae emerging beyond fringe of appressed scales near ventral margin of eye; vibrissae achieving a maximum length 3/5–2/3 × anterior-posterior length of eye.

*Scutellum.* Margins straight.

*Pleurites.* Metepisternum covered by elytron near posterior 1/2 of metasternum.

*Thoracic sterna.* Mesocoxal cavities separated by distance 1/4 × width of mesocoxal cavity. Metasternum with transverse sulcus apparent; metacoxal cavities separated by 2–2.5 × their width.

*Legs.* Tibiae and trochanters of all legs with a single, hair-like, brown seta positioned on mesal surface, approximately 1.5–2 × length of adjacent setae. Profemur/pronotum length ratio 0.93–1.06; profemur with distal 1/5 produced ventrally as an obliquely rounded projection covering tibial joint. Protibia/profemur length ratio 0.84–0.93; protibia moderately stout; mucro present as a laterally projected tooth equal in length and nearly 2 × as wide as nearby setae, triangular. Protarsus with tarsomeres I-II jointly similar in length to V. Metatibial apexwith almond shaped convex ity ringed by 9–10 short, spiniform setae.

*Elytra.* Length/width ratio 3.14–3.24; widest at anterior 1/3–2/5; anterior margins jointly 1.5–1.75 × wider than posterior margin of pronotum; lateral margins sub-parallel after anterior 1/6, more strongly rounded and converging in posterior 1/3. Elytra in lateral view slightly convex; posterior declivity angled at nearly 80° to main body axis. Elytral striae deeply and distinctly punctate; punctures separated by 2–4 × their diameter.

*Abdominal sterna.* Ventrite III elevated and set off from IV along lateral 1/3s of its length. Sternum VII mesally 1/2–3/5 as long as wide, sub-triangular; anterior margin weakly curved. *Tergum.* Pygidium (tergum VIII) sub-conical; medial 1/2 of anterior 1/3 of pygidium less sclerotized.

*Sternum VIII.* Anterior laminar edges each incurved forming a 115° angle with lateral margin; a less sclerotized region present anteriorly with anterior and lateral edges straight, latter diverging anteriorly; sclerotized region with pores throughout, less sclerotized medially; posterior edge strongly incurved and alate.

*Ovipositor.* Coxites less sclerotized postero-laterally, becoming more sclerotized anteriorly and medially, sclerotized regions porose, 2/3 as broad as long in dorsal view; styli 2/3 × length of coxites (latter short).

*Spermatheca.* Comma-shaped; collum expanded, apically with a long, perpendicular, cylindrical projection, nearly equal in length to collum; collum short, slightly swollen, 2/3 length of ramus, sub-contiguous with, and angled at 90° to ramus; ramus elongate, bulbous, 2 × thickness of corpus, basally constricted; corpus not swollen, equal in width to cornu; cornu elongate, apically, gradually narrowed, strongly recurved in basal 1/2, forming an inner angle of ca. 45° to collum and corpus, abruptly bent outward ca. 60°, then incurved.

#### Male.

Similar to female, except where noted. Length 2.77–3.12 mm, width 0.98–1.16 mm, length/width ratio 2.58–2.90. Rostrum length 0.40–0.50 mm, rostrum/pronotum length ratio 0.57–0.73, rostrum length/width ratio 1.05–1.17. Pronotum length/width ratio 0.86–0.91. Profemur/pronotum length ratio 0.97–1.22, protibia/profemur length ratio 0.84–0.88. Elytra length/width ratio 1.23–3.15.

*Elytra.* Elytral declivity less angulate, forming a 60° angle to main body axis, but otherwise as female.

*Abdomen.* Sternum VII 2/5–1/2 × as long as wide. Tergum VII with posterior margin straight. Pygidium (tergum VIII) with posterior margin arcuate laterally, straight along mesal 1/3; posterior 2/3 punctate; anterior 1/3 rugose.

*Sternum VIII.* Lateral and mesal margins straight. Laminar alae located on lateral 1/3 of posterior margin, 1/2 as long as sub-quadrate portion of lamina. Mesal 1/3 without projection. Tegmen. 2/3 × length of pedon. Aedeagal pedon. Length/width ratio 5.89–7.82; lateral margins parallel, abruptly, more strongly converging in region of ostium. In lateral view, width of pedon even throughout in anterior 2/3, ventral margins in posterior 1/3 becoming straight towards apex, then curving to meet dorsal margins at a sharp apical point; apexacutely angulate. Flagellum apically with a reduced conical sclerite. Aedeagal apodemes slightly shorter than pedon.

**Figure 33.**
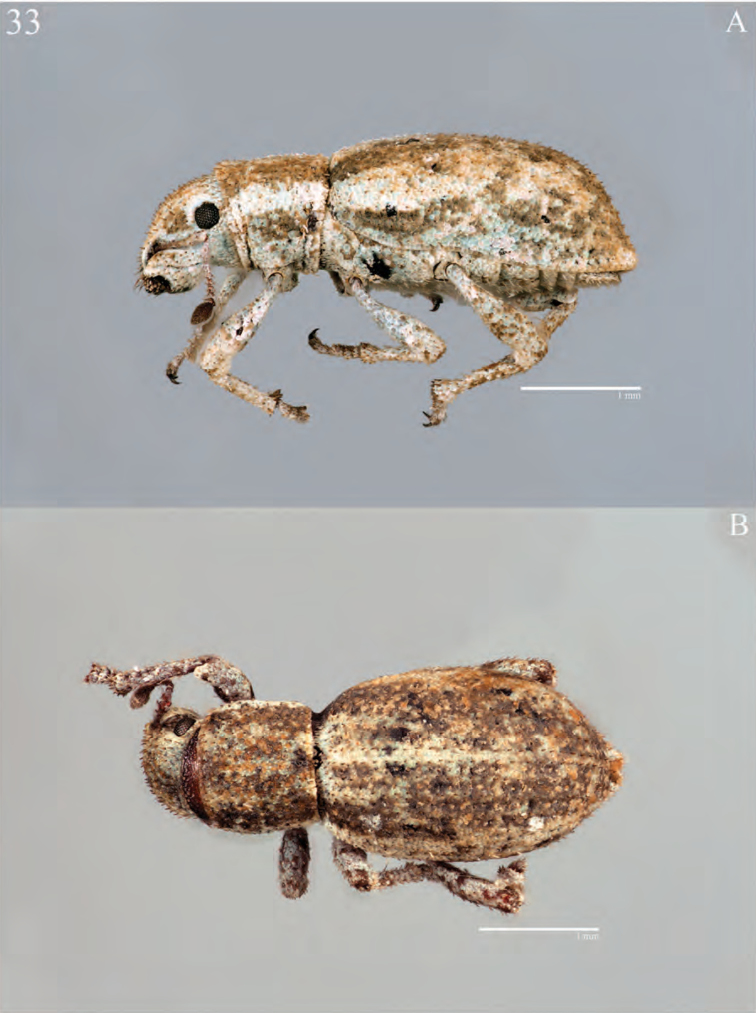
Habitus of *Minyomerus puticulatus* [JF2015], female **A** lateral view **B** dorsal view.

**Figure 34.**
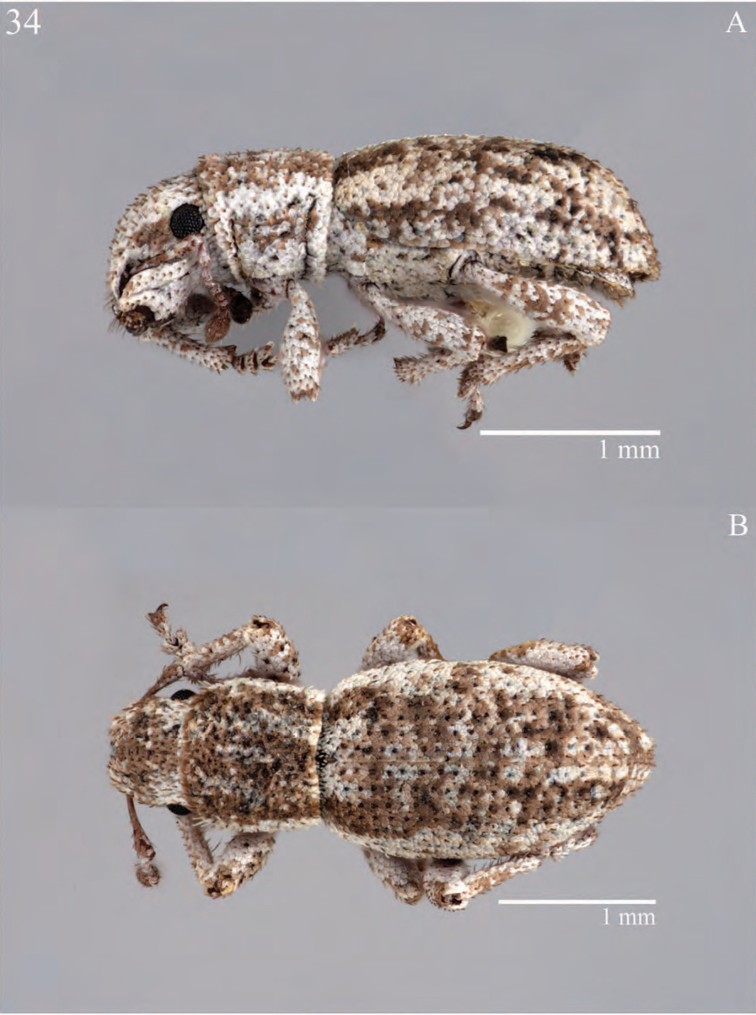
Habitus of *Minyomerus puticulatus* [JF2015], male **A** lateral view **B** dorsal view.

**Figure 35.**
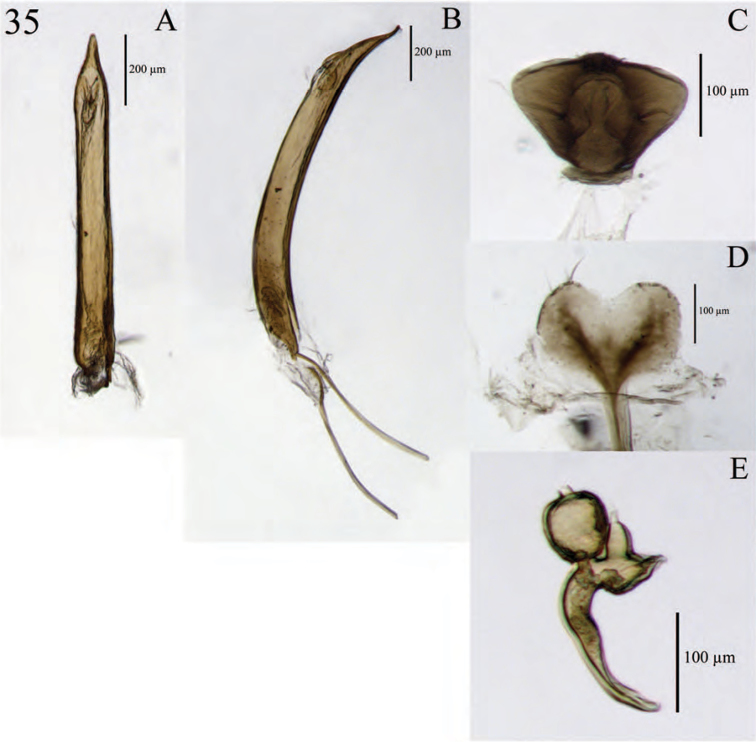
Diagnostic features and terminalia of *Minyomerus puticulatus* [JF2015] **A** aedeagus, dorsal view **B** aedeagus, lateral view **C** labial prementum, ventral view **D** lamina of sternum VIII **E** spermatheca.

#### Etymology.

Named in reference to the deep and distinct punctures covering the dorsum; *puteus* = pit, *puticulus* = diminutive of pit (small pit), *puticulatus* = with little pits; Latin adjective (Brown 1956).

#### Material examined.

Holotype – female “TEX. Big Bend, N.P. 6.5 mi. S, W. Grapevine, Hills Ranch/ at night, VI-3-1970, L & C.W. O’Brien” (CWOB); Paratypes, same label information as female holotype (CWOB: 10 females, 8 males) [3 females, 2 males deposited at CMNC]; “TEX. Terrel Co., 25 mi. SE. Dryden, 8-X-1982, E.G. Riley” (CWOB: 6 females, 10 males) [2 females, 3 males deposited at CMNC]; “TEX. Big Bend, N.P. 6.5 mi., SW. Grapevine, Hills Ranch/ *Parthenium incanum* [non-focal], at night, 3-26-1970” (CWOB: 4 females, 1 male); “TEX. Big Bend, N.P. 6.5 mi., SW. Grapevine, Hills Ranch/ at night, L & C.W. O’Brien, III-27-1970” (CWOB: 2 females, 5 males); “TEX. Big Bend, N.P. 2.5 mi. S, W. Grapevine, Hills Ranch/ at night, L & C.W. O’Brien, VI-3-1970” (CWOB: 2 females, 2 males); “TEX. Big Bend, N.P. 5300’, Green Gulch, VI-8-1970/ *Parthenium incanum*[non-focal], at night, L & C.W. O’Brien” (CWOB: 1 male); “Dryden Tex, VI-11-1930/ B.E. WHITE, Collection, 1962 Gift/ *Minyomerus*, Det. R.S. Anderson 1994” (CAS: 2 males); “Chisos Mts, VII 18 Tex/ J.W. Green, Collector/ J.W. Green, Collection” (CAS: 2 females); “Chisos Mts, VII 19 Tex/ J.W. Green, Collector/ J.W. Green, Collection” (CAS: 1 females); “Sanderson, Texas, VI-10-30/ JO Martin, Collector/ *Isodacrys ovipennis* Schffr. [misidentification]” (CAS: 4 females, 5 males).

#### Distribution.

This species has been found in the Big Bend region of Texas (USA), and is likely also found in Coahuila and Chihuahua (Mexico) (Fig. 52).

#### Natural history.

Associated with creosote bush (*Larrea tridentata* [DC.] Coville [non-focal]; Zygophyllaceae [non-focal]) and Mariola (*Parthenium incanum* Kunth [non-focal]; Asteraceae [non-focal]).

### Minyomerus bulbifrons [JF2015]

Taxon classificationAnimaliaColeopteraCurculionidae

Jansen & Franz sec. Jansen & Franz (2015) sp. n.

http://zoobank.org/A371520C-4132-4F1F-817B-A8B3D27FFFE8

Figs 36 , 37

#### Diagnosis.

*Minyomerus bulbifrons* [JF2015] is readily differentiated from other congenerics by the heavily protuberant frons (which can extend up to 3 × the length of the eye from the anterior margin of the eye), strongly impressed nasal plate, and punctate elytra. The elytra are angled at their point of contact with the pronotum such that they appear confluent with the posterior margin of the pronotum. The elytral setae are arranged in regular rows, and are small, subrecumbent, and brown. The spermatheca is distinct, with the ramus basally tapered, and the corpus possessing an annulate, cylindrical projection nearly 1/2 × length of the ramus. The aedeagal flagellum is unique in possessing a spiriform apical sclerite that spirals counterclockwise and is as long as the aedeagal pedon.

#### Description – female.

*Habitus.* Length 3.67–4.10 mm, width 1.41–1.60 mm, length/width ratio 2.49–2.61, widest at anterior 1/5 of elytra. Integument dark reddish-brown to black. Scales slightly off-white to manila/tan to beige, in some specimens appearing semi-translucent (in others opaque) or metallic; occasionally monotonic and off-white, but usually with interspersed colors forming small maculae, bands and other variously scattered patterns; scales generally off-white ventrally, including rows of setae. Setae minute, sub-recumbent, of equal length throughout.

*Mandibles.* Covered with whitish scales, with 2–3 longer setae, and 3–5 shorter interspersed setae.

*Maxillae.* Cardo bifurcate at base with an inner angle of ca. 105°, arms roughly equal in length and width, arms of bifurcation equal in length to apically outcurved arm. Stipes nearly square, equilateral, roughly equal in length to inner arm of bifurcation of cardo, with a single lateral seta. Galeo-lacinial complexmembranous and setose in posterior 2/3, sclerotized and somewhat emarginate anteriorly; dorsally with 9 apicomesal lacinial teeth; ventrally with 4 reduced lacinial teeth. Palpiger with a transverse row of setae; anterior 1/3 membranous, posteriorly sclerotized.

*Maxillary palps*. Palpomere I with apical end facing mesally and forming a 30° angle with base, I with 2 apical setae, II with 3 dorsal, 1 apical, and 1 mesoventral setae.

*Labium.* Prementum roughly pentagonal; apical margins sinuate, ligula broadly rounded, lateral margins broadly sinuate, posterior margin curved. Labial palps 3-segmented, I with apical 1/3 projecting beyond margin of prementum and apexof ligula.

*Rostrum.* Length 0.53–0.59 mm, anterior portion ca. 2.5 × broader than long, rostrum/pronotum length ratio 0.60–0.75, rostrum length/width ratio 1.00–1.14. Dorsal outline of rostrum square, anterior half of dorsal surface mesally concave, posterior half strongly convex and punctate. Rostrum in lateral view nearly square; anterior half dorsolateral margins slightly diverging; apical margin with 2 groups of 3 large vibrissae, each group inserted just laterad of apexof each sinuation. Nasal plate defined by V-shaped, impressed lines, concave, integument covered with white scales. Margins of mandibular incision only very slightly diverging dorsally in frontal view. Ventrolateral sulci strongly defined as a deep notch dorsad of insertion point of mandibles.

*Antennae.* Dorsal margin of scrobe overhanging slightly and forming a minute tooth, anterior to margin of eye by 1/3 of length of eye. Club 2.5–3.0 × as long as wide.

*Head.* Eyes separated in dorsal view by 5–6 × their anterior-posterior length, set off from anterior prothoracic margin by 2/5–1/2 of their anterior-posterior length. Head between eyes punctate and strongly protuberant.

*Pronotum.* Wider than long, length/width ratio 0.75–0.83. Anterior margin slightly curved and somewhat produced dorsally; anterior constriction subtle in some specimens, posterior margin incurved. Pronotum in lateral view with setae inserted 2 × their length from anterior margin. Anterolateral margin with a reduced tuft of post-ocular vibrissae present, consisting of 3–5 setae, emerging near ventral 1/4 of eye, stopping just beyond ventral margin of eye; vibrissae achieving a maximum length 1/2 × anterior-posterior length of eye.

*Pleurites.* Metepisternum covered by elytron near posterior 1/8 of metasternum.

*Thoracic sterna.* Mesocoxal cavities separated by 1/6 × width of mesocoxal cavity. Metasternum with a somewhat obscure transverse sulcus; metacoxal cavities separated by 2–2.5 × their width.

*Legs.* Tibiae and trochanters of all legs with a single, hair-like, brown seta positioned on mesal surface, approximately 1.5–2 × length of adjacent setae. Profemur/pronotum length ratio 0.97–1.061; profemur with distal 1/5 produced ventrally as an obliquely rounded projection covering tibial joint. Protibia/profemur length ratio 0.91–0.99; protibia moderately stout; mucro present as a laterally projected tooth equal in length and nearly 2 × as wide as nearby setae, triangular. Protarsus with tarsomeres I-II jointly similar in length to V. Metatibial apexwith almond shaped convex ity ringed by 9–10 short, spiniform setae.

*Elytra.* Length/width ratio 3.16–3.46; widest at anterior 1/5; anterior margins jointly 1.5–1.75 × wider than posterior margin of pronotum; lateral margins slightly converging after anterior 1/5, more strongly rounded and converging in posterior 1/3. Posterior declivity broadly arcuate dorsally, slightly inflected thereafter, angled at 45–50° to main body axis. Punctures distinct, separated by 3–5 × their diameter; intervals elevated and with small inapparent puntures, separated by 10–15 × their diameter; scales occasionally monotonic off-white.

*Abdominal sterna.* Ventrite III elevated and set off from IV along lateral 1/3 of its length. Sternum VII mesally 1/2–3/5 as long as wide; anterior margin straight. *Tergum.* Pygidium (tergum VIII) entirely sub-cylindrical; medial 1/3 of anterior 1/2 of pygidium less sclerotized.

*Sternum VIII.* Anterior laminar edges each incurved forming a 105° angle with lateral margin, sclerotized region less sclerotized medially. *Ovipositor.* Coxites 2/3 as broad as long in dorsal view.

*Spermatheca.* Comma-shaped; collum short, apically with a large, hood-shaped projection perpendicular to ramus, nearly equal in length and contiously aligned with curvature of bulb of ramus and ante-apically with a long, perpendicular, cylindrical projection, nearly equal in length to collum and 1/2 length of ramus; collum short, cylindrical, sub-contiguous with, and angled at 90° to ramus; ramus elongate, bulbous, equal in thickness to corpus; corpus swollen, slightly thicker than collum, 1.5 × thickness of cornu; cornu elongate, apically, gradually narrowed, strongly recurved in basal 1/2, forming an inner angle of ca. 60° to collum and corpus, abruptly bent outward ca. 30°, then incurved.

**Figure 36.**
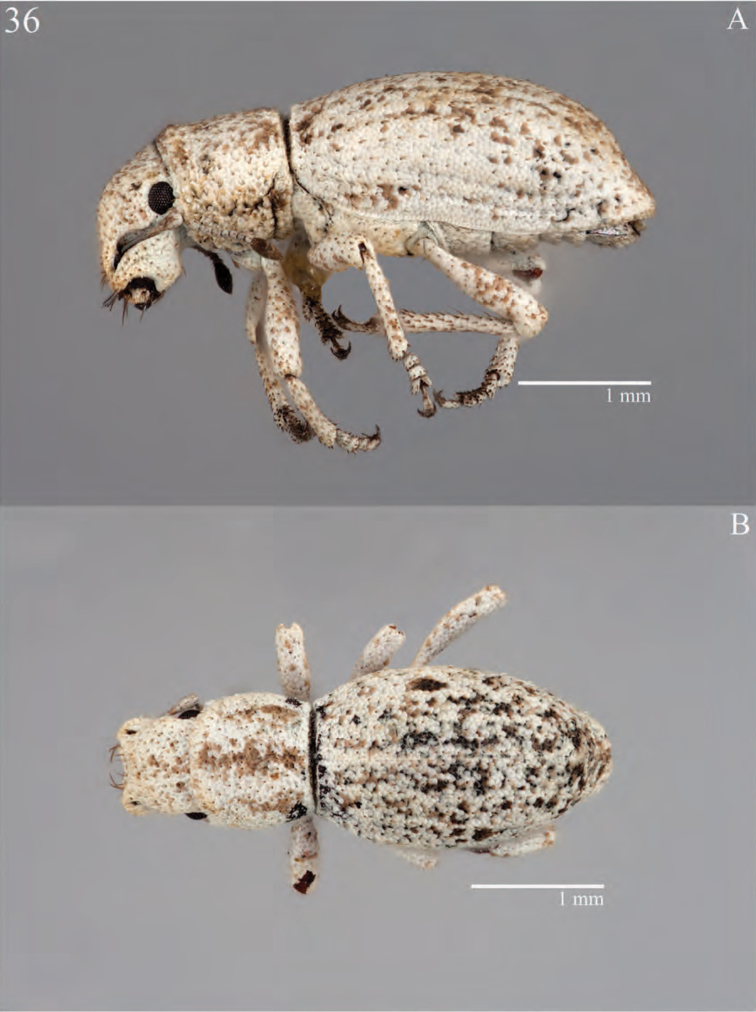
Habitus of *Minyomerus bulbifrons* [JF2015], female **A** lateral view **B** dorsal view.

**Figure 37.**
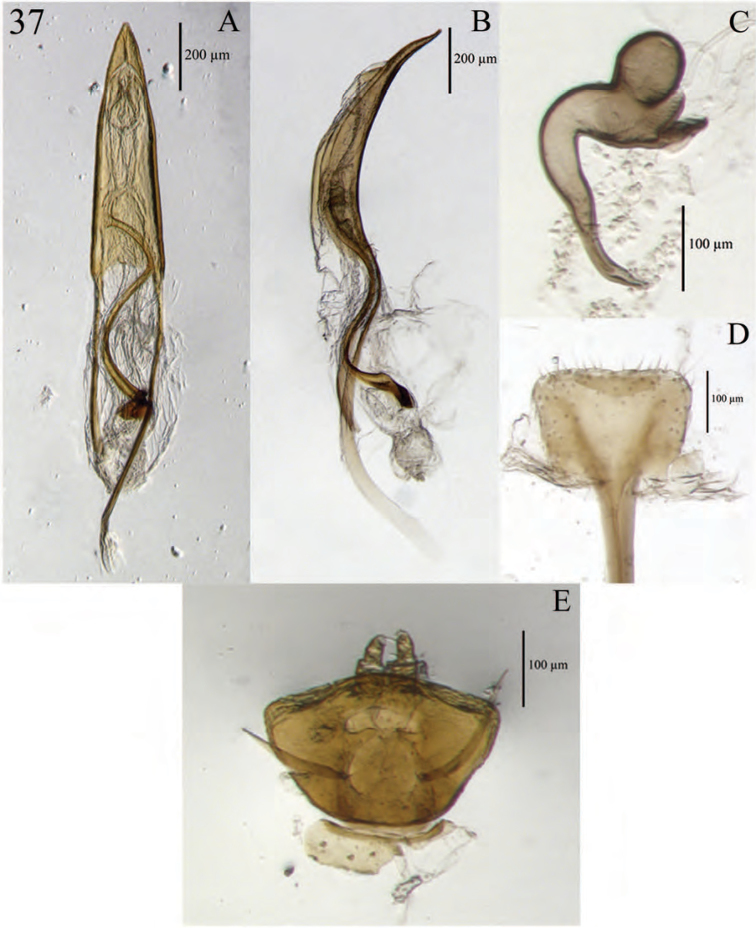
Diagnostic features and terminalia of *Minyomerus bulbifrons* [JF2015] **A** aedeagus, dorsal view **B** aedeagus, lateral view **C** spermatheca **D** lamina of sternum VIII **E** labial prementum, ventral view.

#### Male.

Similar to female, except where noted. Length 3.26–3.44 mm, width 1.20–1.28 mm, length/width ratio 2.59–2.78. Rostrum length 0.46–0.56 mm, rostrum/pronotum length ratio 0.61–0.78, rostrum length/width ratio 0.95–1.08. Pronotum length/width ratio 0.72–0.83. Profemur/pronotum length ratio 1.05–1.30, protibia/profemur length ratio 0.92–0.96. Elytra length/width ratio 3.24–3.59.

*Elytra.* Elytral declivity slightly more angulate, forming a 55–60° angle to main body axis, but otherwise as female.

*Abdomen.* Sternum VII 2/5–1/2 × as long as wide. Pygidium (tergum VIII) with posterior 2/3 punctate; anterior 1/3 rugose.

*Aedeagus.* Length/width ratio 2.83–3.29. Flagellum with a large, narrowly elongate, tortuous, spiriform sclerite, sclerite anteriorly gradually widened and more sclerotized, constricted slightly in anterior 1/8 and slightly widening anteriorly to form a small bulb and long counterclockwise-spiraling projection, situated in anterior portion of flagellum, and as long as pedon.

#### Etymology.

Named in reference to the greatly expanded and protuberant frons; *bulbus* = bulbous, *frons* = forehead; *bulbifrons* = with a bulbous forehead; Latin invariable adjective (Brown 1956).

#### Material examined.

Holotype – female “Gila Bend, Ariz. 10m N, July 22, 1924/ EP Van Duzee, Collector” (CAS). Paratypes, same label information as female holotype (CAS: 22 females, 19 males); “Cal, Providence Mts. S. Bdo. Co., Bonanaza King Mine, 4-23-1966/ on *Larrea tridentata* [non-focal], at night, C.W. O’Brien” (CWOB: 1 male); “ARIZONA, Ehrenberg, V-21-1939, EP Van Duzee/ “*Elissa*” DET. A.T. HOWDEN” (CAS: 1 female); “USA: Ariz.: Yuma Co., 12 mi. W. Dateland, Hwy 80, 13-II-1970, P.H. Arnaud, Jr./ Swept from flowers, *Encilia farinosa* A. Gray [non-focal]” (CAS: 1 female); “Potholes Cal., Imperial Co., April 10, 1923/ EP Van Duzee, Colletor/ ex. *Larria mexicana* [non-focal]” (CAS: 1 female, 1 male); “NEVADA, Clark Co., 5 mi. NE Jct I-15 & Carp Rd., VIII-27-1977, Andrew H. Barnum” (BYU: 1 female, 2 males); “ARIZ., Mohave Co., Mt. Trumbull Road, 18 mi. S Utah Line, VIII-28-1977, Andrew H. Barnum” (BYU: 1 female); “UTAH, Washington Co., Beaver Dam Mts. at Ariz. Line, VII-16-1973, A. H. BARNUM” (BYU: 1 female); “UT: Washington Co., Beaver Dam Well, Beaver Dam Wash, 28 Sept 1985, Mower & Nelson” (BYU: 1 female); “Telegraph Pass, Yuma, Ariz, 2-18-1936/ A. T. McClay, Collector/ *Elissa constricta*?” (UCDC: 5 females, 3 males).

#### Distribution.

This species has been found in the Mojave and Sonoran Desert regions of Arizona, California, Nevada, and Utah (USA). It is likely that this species is also present in Sonora and Baja California (Mexico), based on the availability and proximity of similar habitat (Fig. 52).

#### Natural history.

Associated with creosote bush (*Larrea tridentata* [DC.] Coville [non-focal]; Zygophyllaceae [non-focal]) and brittlebush (*Encilia farinosa* Torr. & A. Gray [non-focal]; Asteraceae [non-focal]).

### Minyomerus politus [JF2015]

Taxon classificationAnimaliaColeopteraCurculionidae

Jansen & Franz sec. Jansen & Franz (2015) sp. n.

http://zoobank.org/BD6DDF9E-296F-4E6E-8F92-5F759D91F4FA

Figs 38 , 39 , 40

#### Diagnosis.

*Minyomerus politus* [JF2015] can be distinguished by the protuberant frons, impressed nasal plate, smooth, unsculpted elytra, and minute, white, elytral setae. The spermatheca is distinct, with the ramus elongate and apically swollen, and the corpus possessing an annulate, cylindrical projection nearly 1/2 × length of the ramus. The aedeagal flagellum is unique in possessing a spiriform apical sclerite that spirals clockwise and is as long as the aedeagal pedon.

#### Description – female.

*Habitus.* Length 3.77–4.13 mm, width 1.53–1.70 mm, length/width ratio 2.35–2.47, widest at anterior 1/4 of elytra. Integument dark reddish-brown to black. Scales opaque white with semi-metallic golden scales interspersed throughout, occasionally semi-translucent, appearing black, with opalescent reflections. Linear setiform scales (‘setae’) sparse throughout, minute, sub-erect to sub-recumbent, translucent, white, arranged in rows on elytral intervals, longer on humeri and venter.

*Mandibles.* Covered with white scales, with 3 long setae.

*Rostrum.* Length 0.44–0.55 mm, anterior portion 1.75–2 × broader than long, slightly narrower than head, rostrum/pronotum length ratio 0.50–0.58, rostrum length/width ratio 1.08–1.20. Dorsal outline of rostrum sub-rectangular, anterior half of dorsal surface mesally concave and impressed, posterior half strongly convex and rugose. Rostrum in lateral view sub-trapezoidal; anterior half of dorsolateral margins slightly diverging; apical margin roughly bisinuate, with 2 large vibrissae. Nasal plate defined by V-shaped, impressed lines, medially slightly concave, integument covered with white scales. Margins of mandibular incision curved, directed 30° outward dorsally in frontal view, bounded by same type of scales as those on remainder of body surface. Ventrolateral sulci strongly defined, beginning as a narrow sulcus dorsad of insertion point of mandibles, running parallel to scrobe, disappearing ventrally. Ventrolateral margins narrowly converging. Rostrum ventrally lacking foveae in line with insertion point of mandibles.

*Antennae.* Dorsal margin of scrobe overhanging slightly and forming a minute tooth, ventrad of anterior margin of eye. Club 2.5 × as long as wide.

*Head.* Eyes separated in dorsal view by 3–4 × their anterior-posterior length, set off from anterior prothoracic margin by 2/5–1/2 of their anterior-posterior length. Head between eyes rugose and strongly bulging.

*Pronotum.* Wider than long, length/width ratio 0.83–0.88, sub-cylindrical to slightly globular; median sulcus absent. Anterior margin arcuate and somewhat produced dorsally, lateral margins evenly curved and widening into a bulge near midpoint; posterior margin straight. Pronotum in lateral view with setae inserted 2 × their length from anterior margin. Anterolateral margin with a reduced tuft of post-ocular vibrissae present, consisting of 3–5 setae, emerging near ventral 1/4 of eye, stopping just beyond ventral margin of eye; vibrissae achieving a maximum length 1/2–3/5 × anterior-posterior length of eye.

*Scutellum.* Not exposed.

*Pleurites.* Metepisternum covered by elytron near posterior margin of metasternum.

*Thoracic sterna.* Mesocoxal cavities separated by distance 1/2 × width of mesocoxal cavity. Metasternum with transverse sulcus somewhat obscure; metacoxal cavities separated by 2.5–3.0 × their width.

*Legs.* Tibiae and trochanters of all legs with a single, hair-like, brown seta positioned on mesal surface, approximately 1.5–2 × length of adjacent setae. Profemur/pronotum length ratio 0.99–1.15; profemur with distal 1/5 produced ventrally as a rounded projection covering tibial joint; condyle of tibial articulation occupying 2/3 of distal surface and 1/5 length of femur. Protibia/profemur length ratio 0.84–0.97; mucro present as a laterally projected tooth sub-equal in length and nearly 2 × as wide as nearby setae, triangular. Metatibial apexwith almond shaped convex ity ringed by 9–11 short, spiniform setae.

*Elytra.* Length/width ratio 2.96–3.04; widest at anterior 1/4; anterior margins jointly 1.5–1.75 × wider than posterior margin of pronotum; lateral margins slightly converging after anterior 1/4, more strongly rounded and converging in posterior 1/2. Posterior declivity angled at 45–50° to main body axis. Elytral striae minutely punctate; punctures separated by 6–10 × their diameter; intervals not noticeably elevated.

*Abdominal sterna.* Ventrite III elevated and set off from IV along lateral 1/3 of its length, somewhat concave anteriorly. Sternum VII mesally 1/2 as long as wide; anterior margin straight.

*Tergum.* Pygidium (tergum VIII) sub-cylindrical; medial 1/3 of anterior 2/3 of pygidium less sclerotized.

*Sternum VIII.* Lamina sub-trapezoidal; anterior edges each incurved forming a 105° angle with lateral margin; sclerotized region with pores anteriorly. *Ovipositor.* Coxites 1/3 as broad as long in dorsal view and with sclerotized regions porose; styli with a patch of pores near base.

*Spermatheca.* Comma-shaped; collum short, apically with a tongue-shaped projection perpendicular to ante-apical projection, nearly equal in length and contiously aligned with curvature of ante-apical projection; spermatheca ante-apically with a long, elongate-conical projection, angled at ca. 25° to ramus, slightly longer than collum and 1/2 length of ramus; collum short, cylindrical, sub-contiguous with, and angled at 90° to ramus; ramus elongate, length 2 × width of corpus, basal 1/2 equal in width to corpus, apical 1/2 slightly bulbous; corpus swollen, slightly thicker than collum, wider than cornu; cornu elongate, apically, gradually narrowed, strongly recurved near mesal 1/3, forming an inner angle of ca. 90° to collum and corpus, slightly incurved near apical 1/3.

#### Male.

Similar to female, except where noted. Length 2.61–3.40 mm, width 1.01–1.32 mm, length/width ratio 2.54–2.57. Rostrum length 0.41–0.53 mm, rostrum/pronotum length ratio 0.65–0.71, rostrum length/width ratio 1.16–1.38. Pronotum length/width ratio 0.82–0.88. Profemur/pronotum length ratio 1.12–1.33, protibia/profemur length ratio 0.82–0.90. Elytra length/width ratio 2.72–3.12.

*Elytra.* Elytral declivity slightly more angulate, forming a 65° angle to main body axis, but otherwise as female. Abdomen – sternum IV relatively shorter, mesally slightly longer and laterally shorter than V and VI jointly. Sternum VII 2/5–1/2 × as long as wide. Pygidium (tergum VIII) posterior 2/3 punctate; anterior 1/3 rugose.

*Sternum VIII.* Dorsal surface with a patch of short, fine setae laterally. Aedeagal pedon. Length/width ratio 3.37. Ventral margins in region of ostium straight. Flagellum with a large, narrowly elongate, tortuous, spiriform sclerite, sclerite anteriorly gradually widened and more sclerotized, constricted slightly in anterior 1/3 and slightly widening anteriorly to form a small bulb and long counterclockwise-spiraling projection, situated in anterior portion of flagellum, and as long as pedon.

**Figure 38.**
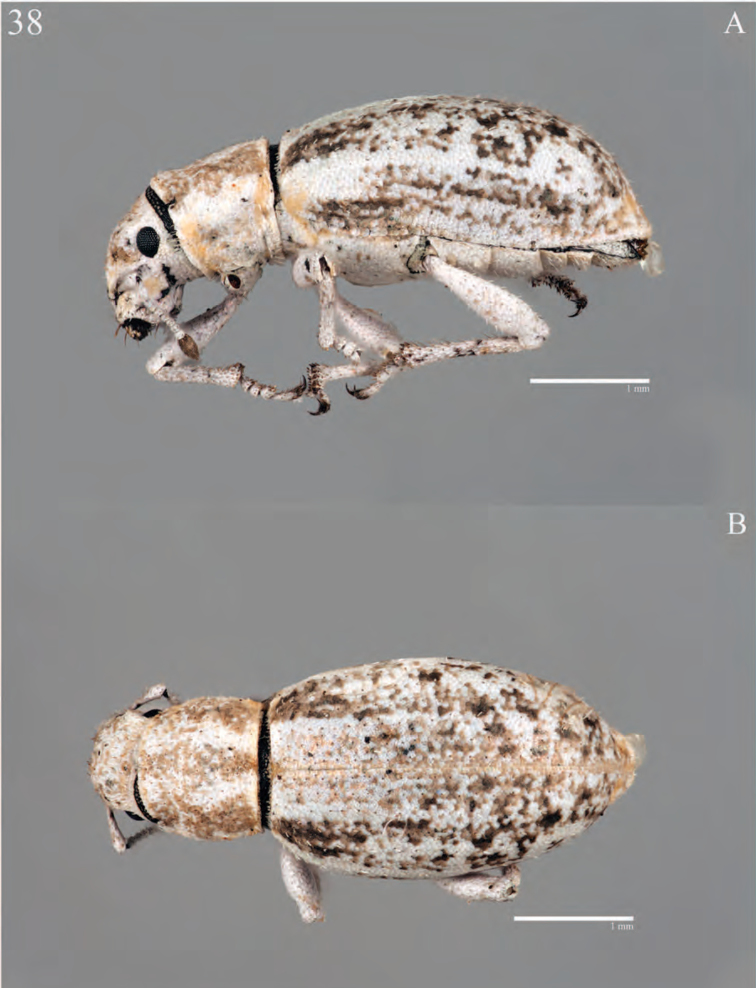
Habitus of *Minyomerus politus* [JF2015], female **A** lateral view **B** dorsal view.

**Figure 39.**
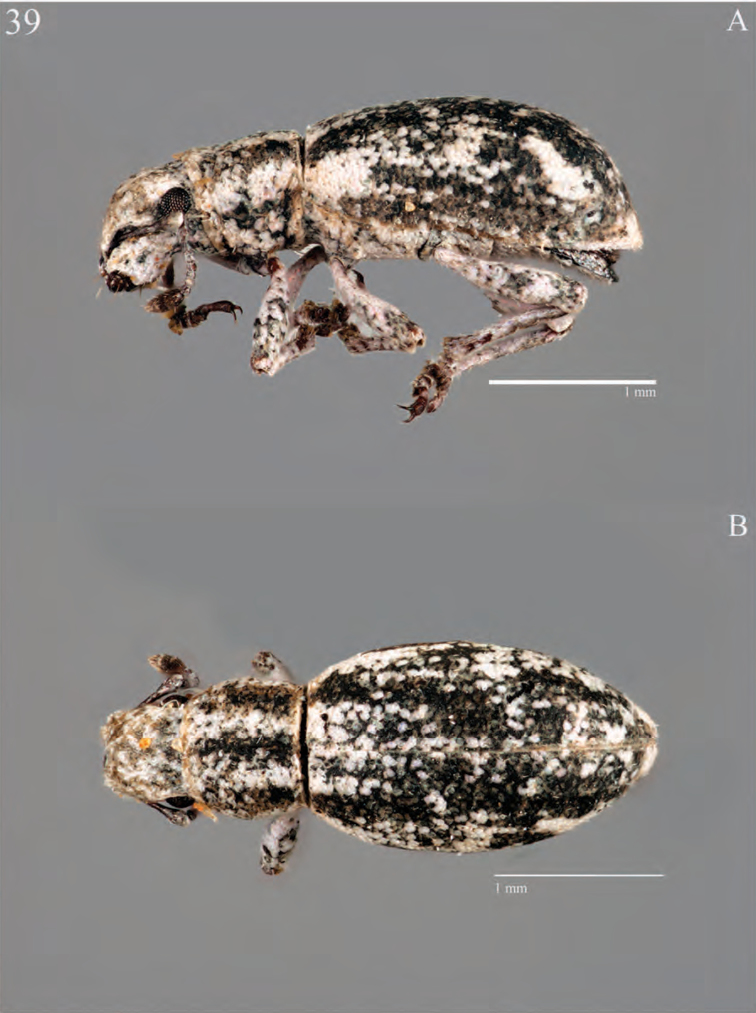
Habitus of *Minyomerus politus* [JF2015], male **A** lateral view **B** dorsal view.

**Figure 40.**
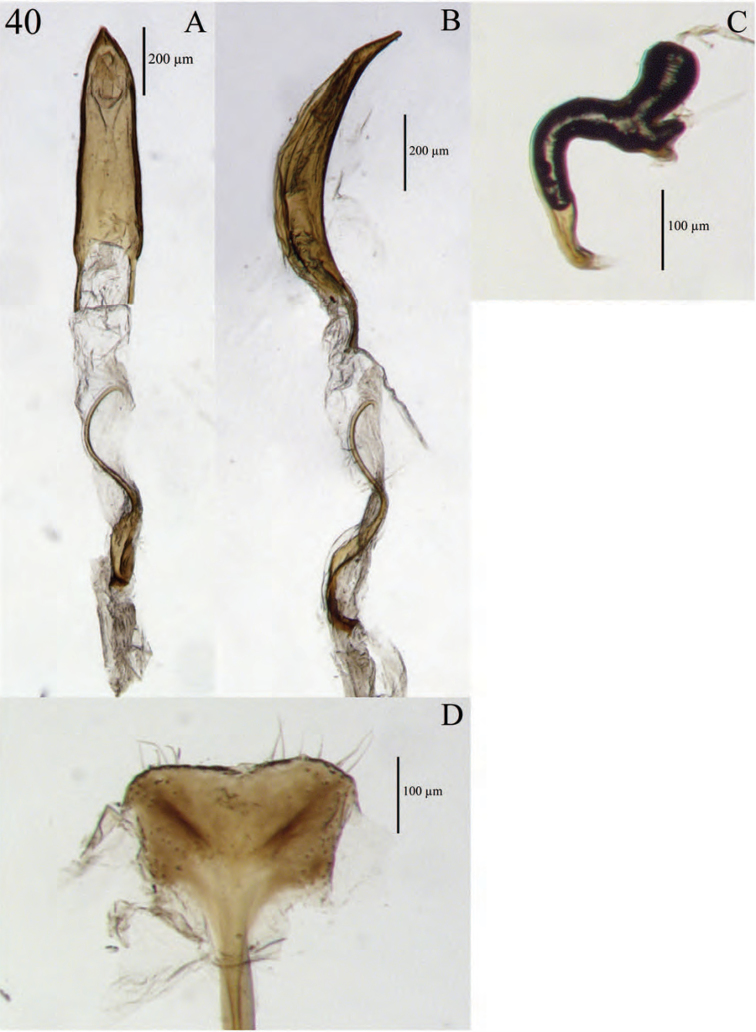
Diagnostic features and terminalia of *Minyomerus politus* [JF2015] **A** aedeagus, dorsal view **B** aedeagus, lateral view **C** spermatheca **D** lamina of sternum VIII.

#### Comments.

Due to the limited number of specimens of this species, dissections of mouthparts could not be performed.

#### Etymology.

Named in reference to the smooth body surface and imperceptibly minute setae; *politus* = polished; Latin adjective (Brown 1956).

#### Material examined.

Holotype – female “NV: Clark Co. Jcn., I-15 & Hwy. 169, 24-IV-2005, C.W. O’Brien/ on *Franseria dumosa* Gray [non-focal]” (CWOB). Paratypes, same label information as female holotype (CWOB: 1 female, 1 male); “NV: Clark Co. Jcn., I-15 & Hwy. 93, exit 64, east, small dunes, IV-8-2005/ C.W. O’Brien, Collector” (CWOB: 2 females, 2 males).

#### Distribution.

This species is known from a single locality in Clark County, Nevada (Fig. 52).

#### Natural history.

Associated with burro-weed (*Ambrosia dumosa* [A. Gray] W.W. Payne [non-focal]; Asteraceae [non-focal]).

### Minyomerus gravivultus [JF2015]

Taxon classificationAnimaliaColeopteraCurculionidae

Jansen & Franz sec. Jansen & Franz (2015) sp. n.

http://zoobank.org/A6353776-CA19-4E7E-B215-D3CB0DA357AE

Figs 41 , 42

#### Diagnosis.

*Minyomerus gravivultus* [JF2015] can be distinguished from other congenerics by a combination of features, and is similar to *Minyomerus languidus* [JF2015] in general appearance. The frons is protuberant in this species, and the nasal plate is covered with scales that have a strongly opalescent reflectance. The elytra are lightly sculpted. The ocular vibrissae are as long as the eye, but reduced in number. The oral cavity has weakly curved lateral margins; the posterior margin is at least as long as the lateral margins. The genae are not impressed with a strong ventrolateral suture. The spermatheca has a bulbous, basally tapered ramus. Additionally, the lamina of the spiculum gastrale is mesally membranous between the laminar arms. The aedeagus is acute and evenly curved.

#### Description – female.

*Habitus.* Length 3.74–4.15 mm, width 1.53–1.64 mm, length/width ratio 2.45–2.53, widest near midpoint of elytra. Integument orange-brown to black. Scales with variously interspersed colors ranging from slightly off-white to manila/tan to dark coffee brown, in some specimens appearing opalescent or with metallic reflections. Setae brown, becoming longer, white, and more erect on humeri and venter.

*Mandibles.* Covered with opalescent scales, each with 2 pairs of long setae.

*Maxillae.* Cardo bifurcate at base with an inner angle of ca. 105°, inner (mesal) arm 2 × longer than outer arm, inner arm of equal width to outer arm, inner arm of bifurcation equal in length to apically outcurved arm, glabrous. Stipes sub-quadrate, 1.5–2 × longer than wide, roughly equal in length to inner arm of bifurcation of cardo, with 1 short, lateral seta. Galeo-lacinial complexapically incurved (mesally); complexmembranous; setose in basal half; dorsally with 7 apicomesal lacinial teeth; ventrally with 5 reduced lacinial teeth. Palpiger with a lateral patch of setae, sclerotized on basal 2/3.

*Maxillary palps*. Palpomeres I and II with apical ends facing mesally and forming a 45° angle with base, with 2 apical setae.

*Labium.* Prementum completely roughly pentagonal; apical margins feebly curved, angulate; lateral margins curved; basal margin arcuate; each lateral region with 1 or 2 long setae. Labial palps 3-segmented, I with apexjust projecting beyond margin of prementum, not reaching apexof ligula; III slightly longer than II.

*Rostrum.* Length 0.56–0.61 mm, anterior portion 1.5–2 × broader than long, rostrum/pronotum length ratio 0.57–0.65, rostrum length/width ratio 1.33–1.39. Dorsal outline of rostrum sub-rectangular, anterior half of dorsal surface strongly impressed, posterior half strongly rugose. Rostrum in lateral view trapezoidal; anterior half of dorsolateral margins somewhat diverging; apical margin with 2 large vibrissae. Nasal plate strongly defined by Y-shaped, impressed lines, medially convex; integument completely covered with strongly opalescent scales. Margins of mandibular incision curved, directed 25° outward dorsally in frontal view, bounded by whitish scales, similar to rest of body; ventrolateral sulci somewhat defined, beginning as a broad, shallow sulcus dorsad of insertion point of mandibles, running parallel to scrobe, becoming fainter posteriorly and disappearing into a fovea ventrally. Dorsal surface of rostrum with median sulcus running from fovea at posterior end of anterior half rostrum to midpoint of posterior half of rostrum; ventrolateral margins slightly converging. Rostrum ventrally lacking foveae in line with insertion point of mandibles. Oral cavity with lateral margins curved.

*Antennae.* Dorsal margin of scrobe overhangs forming a small tooth antero-ventrad of anterior marginof eye. Funicular antennomeres evenly progressing from elongate to broader than long; terminal segment lacking appressed scales, having instead a covering of apically-directed pubescence with interspersed sub-erect setae. Club similar in length to funicular antennomeres III-VII, nearly 2.5 × as long as wide.

*Head.* Eyes strongly impressed; eyes separated in dorsal view by 4–5 × their anterior-posterior length, narrowly separated from anterior prothoracic margin.

*Pronotum.* Length/width ratio 0.91–1.01, sub-cylindrical to conical; widest near anterior 1/4. Anterior margin arcuate, lateral margins feebly curved and widening into a slight bulge near anterior 1/3 of pronotum, thence straight to posterior margin, posterior margin straight. Pronotum in lateral view with setae that reach beyond anterior margin; these setae becoming evenly longer laterally, reaching posterior margin of eye at their maximum length. Anterolateral margin with a reduced tuft of post-ocular vibrissae present, consisting of 3–5 setae, emerging slightly above ventral margin of eye, becoming gradually, evenly longer ventrally, stopping just beneath ventral margin of eye; vibrissae achieving a maximum length nearly equal to anterior-posterior length of eye.

*Pleurites.* Metepisternum covered by elytron near posterior 7/8 of metasternum.

*Thoracic sterna.* Mesocoxal cavities separated by distance1/3 × width of mesocoxal cavity. Metasternum without apprent transverse sulcus; metacoxal cavities widely separated by 1.5–2 × their width.

*Legs.* Profemur/pronotum length ratio 0.93–1.02; proximal 5/6 of profemur gradually widening, then abruptly constricted with distal 1/6 produced ventrally as a nearly square projection covering tibial joint; condyle of tibial articulation occupying 2/3 of distal surface and 1/6 length of femur. Protibia/profemur length ratio 0.88–0.99; mucro reduced to a small, laterally projected tooth. Protarsus with tarsomeres I and II jointly similar in length to V. Metatibial apexwith almond shaped convex ity ringed by 9-11 short, widely separated, spiniform setae.

*Elytra.* Length/width ratio 3.00-6.80; widest near midpoint; anterior margins jointly 1.5-2 × wider than posterior margin of pronotum; lateral margins sub-parallel after anterior 1/4, more strongly rounded and converging in posterior 1/2. Posterior declivity angled at nearly 60° to main body axis. Elytral striae well defined, punctate; punctures visible in some specimens, separated by 3-5 × their diameter.

*Abdominal sterna.* Ventrite III elevated and set off from IV along lateral 1/4 of its length. Sternum VII mesally 3/5 × as long as wide; anterior margin weakly curved. *Tergum.* Pygidium (tergum VIII) sub-cylindrical; medial 1/3 of anterior 1/2 of pygidium less sclerotized.

*Sternum VIII.* Anterior laminar edges each incurved forming a 100° angle with lateral margin; less sclerotized medially.

*Ovipositor.* Coxites slightly sclerotized, in dorsal view 2 × as long as broad; styli 3/4 × length of coxites, with 3 long setae near base.

*Spermatheca.* Comma-shaped; collum short, sub-contiguous with, and angled at 90° to ramus; ramus bulbous, 2 × width of cornu; corpus swollen, 1.5 × thickness of cornu; cornu elongate, apically gradually narrowed, strongly recurved with an inner angle of 90° in basal 1/4, nearly sraight along mesal 1/2, and incurved 90° near apical 1/4 such that apexis sub-parallel to hood-shaped projection of collum.

#### Male.

Similar to female, except where noted.

*Abdomen.* Sternum VII slightly more broadly arcuate posteriorly, 3/5 as long as wide. Tergum VII with posterior margin straight. Pygidium (tergum VIII) with posterior 1/2 punctate; anterior 1/2 rugose. Posterior 1/5 constricted and depressed, with posterior margin flaring out and slightly projected dorsally.

*Sternum VIII.* Consisting of 2 subcontiguous, sub-triangular sclerites; posterior margins widely angulate. Laminar alae located on lateral 1/4s of posterior margin. Mesal 1/2 with a short, sub-trapezoidal projection.

*Aedeagus.* Length/width ratio 3.41. Pedon in lateral view with ventral margins curving to meet dorsal margins at a sharp apical point; apexacutely angulate. Flagellum with a small tortuous, recurved, ampullate sclerite, situated in anterior portion of flagellum.

**Figure 41.**
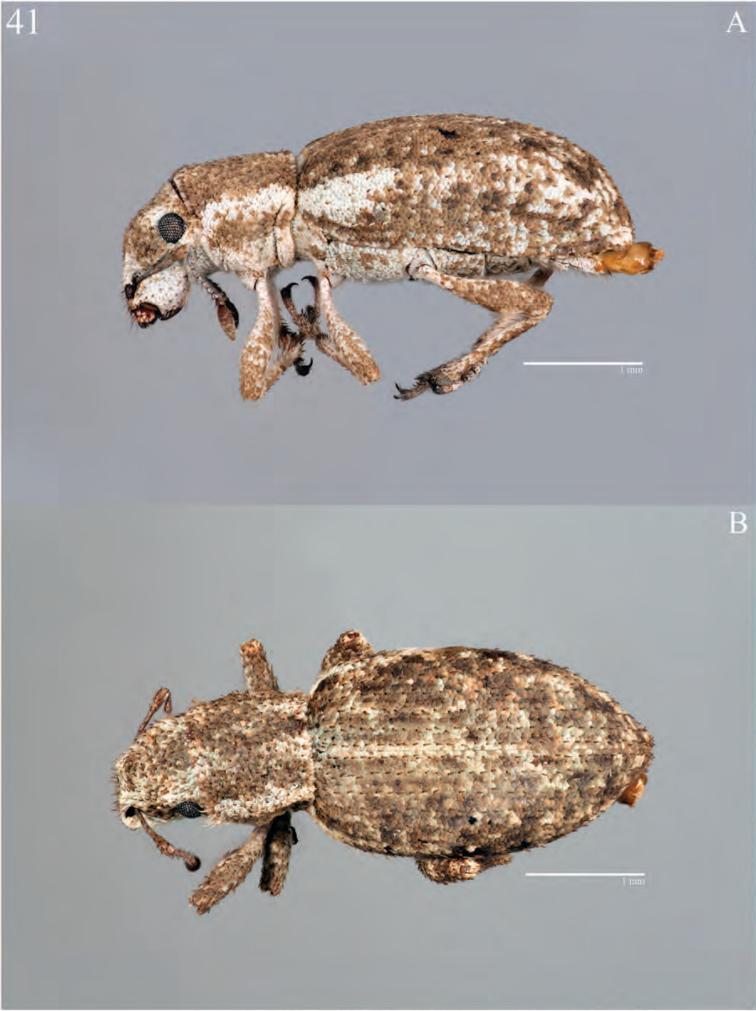
Habitus of *Minyomerus gravivultus* [JF2015], female **A** lateral view **B** dorsal view.

**Figure 42.**
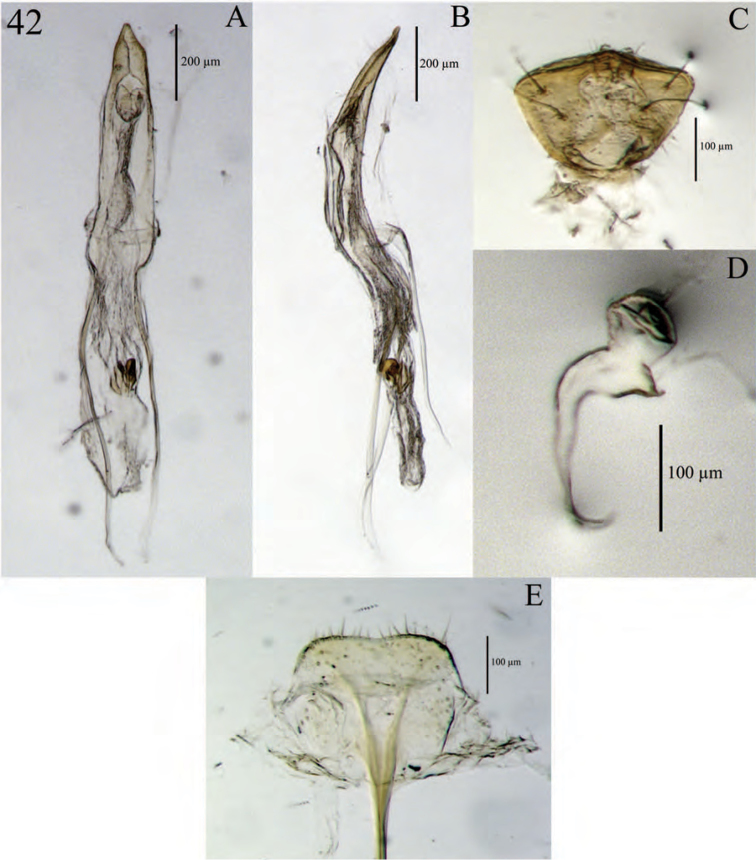
Diagnostic features and terminalia of *Minyomerus gravivultus* [JF2015] **A** aedeagus, dorsal view **B** aedeagus, lateral view **C** labial prementum, ventral view **D** spermatheca **E** lamina of sternum VIII.

#### Etymology.

Named in reference to the enlarged supraorbital ridges, which give the impression of a furrowed brow; *gravis* = heavy or serious, *vultus* = countenance or face, *gravivultus* = serious countenance; Latin noun in apposition (Brown 1956).

#### Material examined.

Holotype – female “MEXICO: Baja California, 5.5 mi. E El Arco, IV-15-1987, F. Andrews & A. Glibert” (CSCA). Paratypes, same label information as female holotype (CSCA: 3 females); “MEXICO: Baja California Sur, 1.9 mi. SW El Arco, IV-16-1987, F. Andrews & A. Gilbert” (CSCA: 1 male).

#### Distribution.

This species has been found in Baja California, near the border of Baja California Sur (Mexico) (Fig. 52).

#### Natural history.

Host plant associations remain unknown, though possibly on creosote bush (*Larrea tridentata* [DC.] Coville [non-focal]; Zygophyllaceae [non-focal]).

### Minyomerus griseus [JF2015]

Taxon classificationAnimaliaColeopteraCurculionidae

(Sleeper, 1960) sec. Jansen & Franz (2015) comb. n.

Figs 43 , 44 , 45

== AND = Piscatopus griseus Sleeper, 1960: 84 sec. Sleeper (1960)

#### Nomenclatural and taxonomic emendations.

Our phylogenetic analysis indicates that numerous significant characters are shared by all species herein assigned to *Minyomerus* [JF2015] and *Piscatopus griseus* sec. Sleeper (1960) which anchors the monotypic genus-level concept *Piscatopus* sec. Sleeper (1960) (see also O’Brien and Wibmer 1982; Anderson 2002). These include (Fig. 49): the widely separated and laterally positioned eyes, the ventrally directed scrobe (with overhanging dorsal margin), and the spiniform setae on the tarsi. The results of the analysis place *Minyomerus griseus* [JF2015] as sister to *Minyomerus rutellirostris* [JF2015], nested well within a clade of species of *Minyomerus* [JF2015] that also entails *Minyomerus gravivultus* [JF2015]. As such, we move to change *Piscatopus* sec. Sleeper (1960) to junior synonymy of *Minyomerus* [JF2015], and therefore also rename its sole member, *Piscatopus griseus* sec. Sleeper (1960), as *Minyomerus griseus* (Sleeper, 1960) [JF2015] in a new combination (see also Discussion).

#### Diagnosis.

*Minyomerus griseus* [JF2015] is readily distinguished from other congenerics by the separated procoxae and the size of the rostrum, which is narrower than the head. The eyes are relatively large, and the elytra are not noticeably punctate. The ramus of the spermatheca is cylindrical, somewhat bulbous, and basally constricted. The aedeagus is elongate, acutely angulate, and narrowing towards the apexmore strongly in the region of the ostium.

#### Redescription – female.

*Habitus.* Length 4.25-4.75 mm, width 1.77-2.03 mm, shape sub-cylindrical to ovate, length/width ratio 2.18–2.58, widest near anterior 1/2 of elytra. Integument dark reddish-brown to black. Scales with variously interspersed colors ranging from grey to white to yellow, in some specimens appearing semi-translucent (in others opaque) or with reddish or greenish opalescent reflections; scale color generally uniform throughout, sometimes with scales becoming lighter ventrally. Setae recumbent.

*Mandibles.* Covered with opalescent scales, with 6–7 longer setae.

*Maxillae.* Cardo as wide as palpomere III, bifurcate at base with an inner angle of ca. 100°, inner (mesal) arm 2 × longer than outer arm and of equal thickness, inner arm of bifurcation equal in length to apically outcurved arm. Stipes nearly square, equilateral, roughly equal in length to inner arm of bifurcation of cardo, glabrous. Galeo-lacinial complexmembranous and setose in posterior 2/3, sclerotized and somewhat emarginate anteriorly; dorsally with 8 apicomesal lacinial teeth; ventrally with 4 reduced lacinial teeth. Palpiger with a transverse row of setae; anterior 1/3 membranous, posteriorly sclerotized.

*Maxillary palps*. Palpomere I with apical end facing mesally and forming a 55° angle with base, I and II each with 2 apical setae; II with 1 mesoventral seta in addition to 2 apical setae.

*Labium.* Prementum roughly pentagonal, ventrally sub-planar throughout; apical margins sinuate, angulate, lateral margins broadly curved, posterior margin arcuate. Labial palps 3-segmented, I with apical 1/5 projecting beyond margin of prementum, but not reaching apexof ligula; III shorter than II.

*Rostrum.* Length 0.54–0.63 mm, anterior portion 2–2.5 × broader than long, narrower than head, rostrum/pronotum length ratio 0.51–0.53, rostrum length/width ratio 0.85–0.95. Dorsal outline of rostrum trapezoidal, posterior half of dorsal surface strongly rugose. Rostrum in lateral view nearly square; apical margin bisinuate, sinuations meeting to form a small median projection, and with 2 large vibrissae. Nasal plate very weakly defined by V-shaped, impressed lines, posteriorly planar, anteriorly concave, integument partially covered with opalescent scales. Margins of mandibular incision directed 25° outward dorsally in frontal view. Ventrolateral sulci weakly defined as a broad concavity dorsad of insertion point of mandibles (slightly notched in some specimens), running parallel to scrobe, becoming flatter posteriorly and disappearing ventrally. Dorsal surface of rostrum with median linear fovea at posterior end of rostrum equal in length to anterior-posterior length of eye. Rostrum ventrally with integument between 2 converging sulci (beginning at corners of oral cavity) slightly elevated. Oral cavity with lateral margins curved.

*Antennae.* Dorsal margin of scrobe overhanging slightly and forming a small tooth, anterior to margin of eye by 1/3 of length of eye. Terminal funicular segment lacking appressed scales, having instead a covering of apically-directed pubescence with interspersed sub-erect setae. Club nearly 3 × as long as wide.

*Head.* Anterodorsal margin of each eye slightly impressed, posterior margin strongly elevated from lateral surface of head; eyes separated in dorsal view by 4–5 × their anterior-posterior length, set off from anterior prothoracic margin by 1/4 of their anterior-posterior length. Head between eyes slightly bulging, appearing flat in some specimens. Head without any transverse post-ocular impression.

*Pronotum.* Length/width ratio 0.68–0.81, globular; surface finely punctate, though punctures somewhat obscured by scales; median sulcus absent. Anterior margin incurving mesally, posterior margin straight. Pronotum in lateral view with setae inserted 2 × their length from anterior margin. Anterolateral margin with a reduced tuft of 6–7 ocular vibrissae present, emerging near posterior 1/2 of eye, vibrissae longer ventrally, stopping near dorsal margin of scrobe, achieving a maximum length equal to 3/4 × anterior to posterior length of eye.

*Scutellum.* Margins straight. *Pleurites.* Metepisternum covered by elytron near posterior 1/4 of metasternu.

*Thoracic sterna.* Prosternal process complete or nearly so between coxae, coxae separated by distance equal to 1/6 × width of procoxal cavity. Mesocoxal cavities separated by distance 2/5 × width of mesocoxal cavity. Metasternum with transverse sulcus apparent; metacoxal cavities separated by 1.5–2 × their width.

*Legs.* Tibiae and trochanters of all legs with a single, hair-like, brown seta positioned on mesal surface, approximately 2 × length of adjacent setae. Profemur/pronotum length ratio 0.92–1.05; proximal 5/6 of profemur gradually widening, then abruptly constricted with distal 1/6 produced ventrally as a semicircular projection covering tibial joint; condyle of tibial articulation occupying 1/6 length of femur. Protibia/profemur length ratio 0.92–1.01; protibia moderately long and stout; protibial apexwith ventral setal comb recessed in a broadly concave groove; mucro present as a laterally projected tooth equal in length to nearby setae, triangular and equilateral. Protarsus with tarsomere I 1.5 × as long as II; II and III equilateral; I-II jointly similar in length to V. Metatibial apexwith almond shaped convex ity ringed by 10–11 short, spiniform setae.

*Elytra.* Length/width ratio 3.12–3.36; widest near anterior 1/2; anterior margins jointly 3/4–1 × width of posterior margin of pronotum; elytra slightly constricted near base, lateral margins gently and evenly curving thereafter, more strongly rounded and converging in posterior 1/4. Posterior declivity angled at nearly 70° to main body axis. Elytral striae finely punctate; punctures indistinct beneath appressed scales, separated by 4–6 × their diameter; intervals not elevated; scale color relatively uniform throughout; each interval medially with a row of recumbent setae.

*Abdominal sterna.* Ventrite III acutely anteromesally concave, posterior margin elevated and set off from IV along lateral 1/4s of its length. Sternum VII mesally 1/2–3/5 as long as wide; anterior margin straight.

*Tergum.* Pygidium (tergum VIII) sub-conical; porose on posterior 1/4; medial 1/3 of anterior 2/3 of pygidium less sclerotized.

*Sternum VIII.* Anterior 9/10 (spiculum ventrale) narrowly stylate; posterior 1/10 (lamina) sub-quadrate; anterior edges each incurved forming a 115° angle with lateral margin; less sclerotized medially; posterior margin medially incurved.

*Ovipositor.* Coxites 2/3 as broad as long in dorsal view; styli digitate, though not apically narrowed.

*Spermatheca.* Comma-shaped; collum highly reduced, apically with a small, hood-shaped projection nearly perpendicular to ramus, 3/5 × length of ramus and convex; ramus elongate, cylindrical, somewhat bulbous, with a basal constriction, 1.5 × thickness of corpus; corpus slightly swollen; cornu elongate, apically, gradually narrowed, strongly recurved in basal 1/3, straight thereafter, extending nearly to extent of projection of collum, forming an inner angle of ca. 45° corpus.

**Figure 43.**
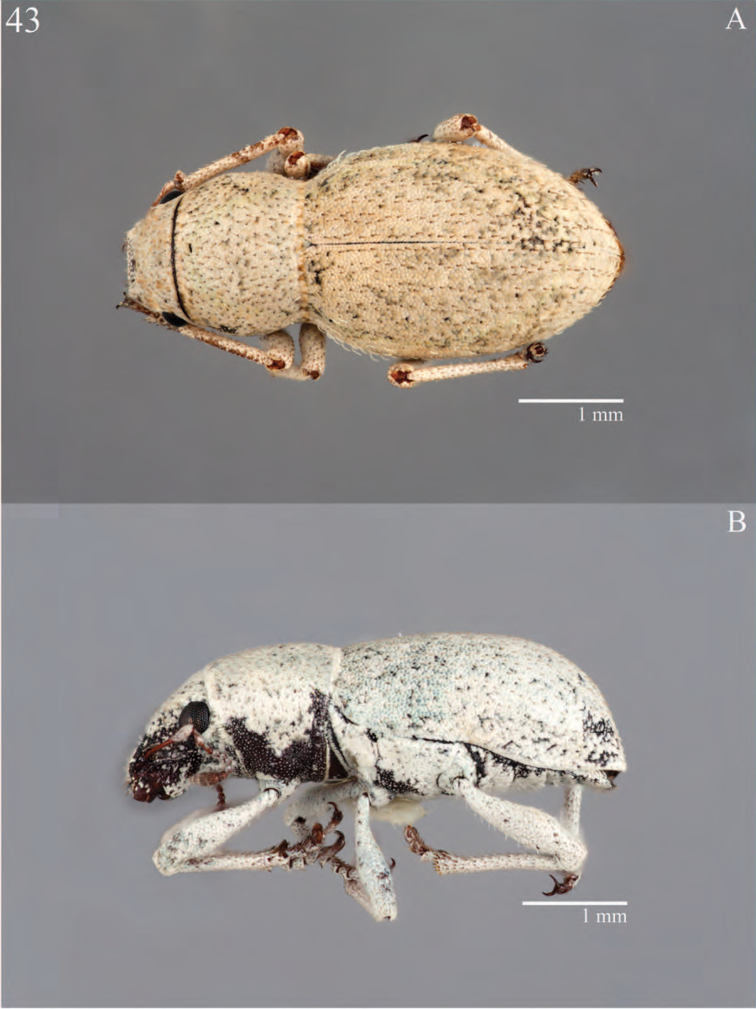
Habitus of *Minyomerus griseus* [JF2015], female **A** dorsal view **B** lateral view.

**Figure 44.**
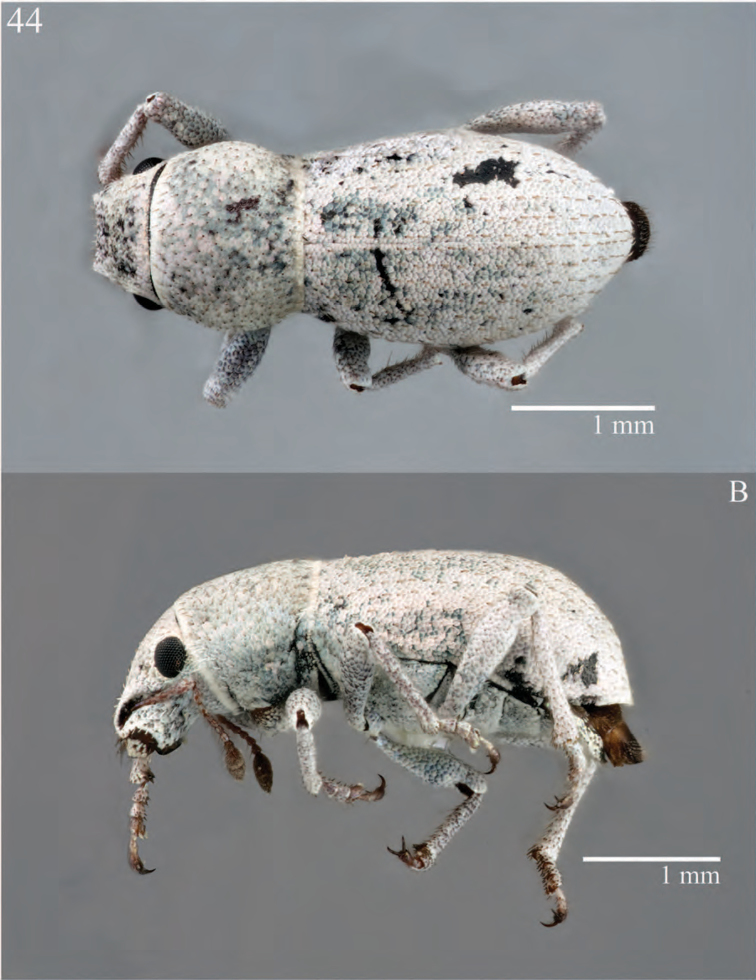
Habitus of *Minyomerus griseus* [JF2015], male **A** dorsal view **B** lateral view.

**Figure 45.**
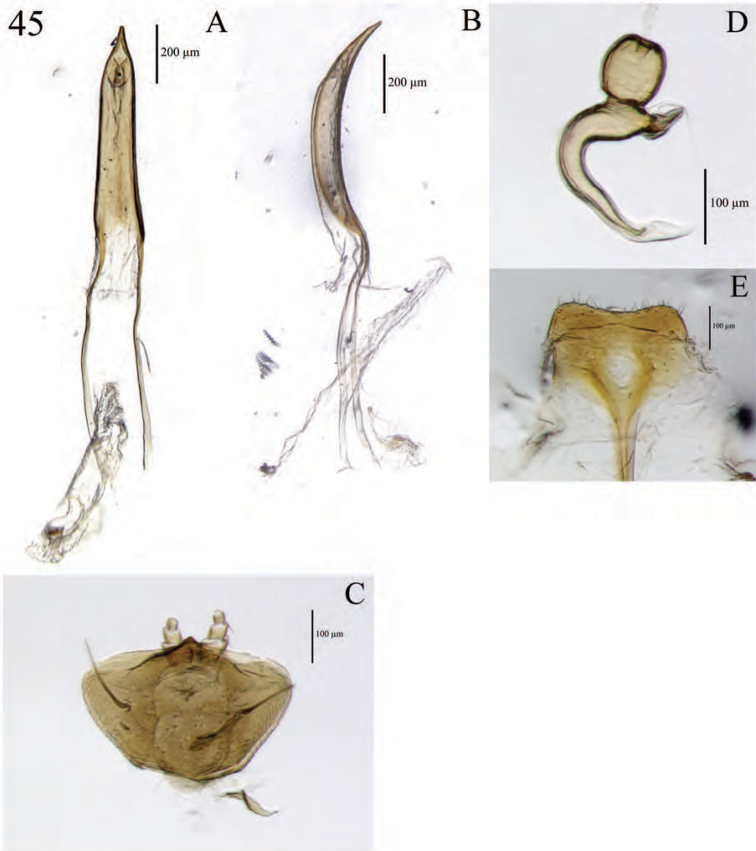
Diagnostic features and terminalia of *Minyomerus griseus* [JF2015] **A** aedeagus, dorsal view **B** aedeagus, lateral view **C** labial prementum, ventral view **D** spermatheca **E** lamina of sternum VIII.

#### Male.

Similar to female, except where noted. Length 4.17–4.37 mm, width 1.49–1.70 mm, length/width ratio 2.53–2.86. Rostrum length 0.55–0.62 mm, rostrum/pronotum length ratio 0.52–0.62, rostrum length/width ratio 0.90–0.98. Pronotum length/width ratio 0.76–0.82. Profemur/pronotum length ratio 0.94–1.16, protibia/profemur length ratio 0.92–1.00. Elytra length/width ratio 3.00–3.55.

*Elytra.* Elytral declivity more angulate, forming an 80° angle to main body axis, but otherwise as female.

*Abdomen.* Sternum VII similar to female. Tergum VII with posterior margin straight. Pygidium (tergum VIII) with posterior margin evenly arcuate; posterior 1/2 punctate; anterior 1/2 rugose.

*Sternum VIII.* Consisting of 2 sub-contiguous, sub-triangular sclerites; posterior margins widely angulate and with 6–8 setae. Spiculum gastrale 1/3 longer than pedon of aedeagus.

*Aedeagus*. Length/width ratio 3.96–4.40; lateral margins slightly converging posteriorly, more strongly converging in region of ostium. In lateral view, width of pedon nearly equal along anterior 3/4, ventral margins in posterior 1/4 becoming straight towards apex, meeting dorsal margins at a sharp apical point; apexacutely angulate. Flagellum with a large, narrowly elongate, tortuous, ampullate, apical sclerite, sclerite anteriorly gradually widened, constricted in anterior 1/4 and slightly widening anteriorly to form a small bulb, situated in anterior portion of flagellum.

#### Material examined.

Allotype – male “Presidio Tex, X-28-44, JH Russel/ On *Larrea divericata* [non-focal] foliage, 44-26373/ ALLOTYPE, *Piscatopus griseus* ♂, Sleeper [red]/ *Piscatopus griseus* Sleeper, Allotype, DET. SLEEPER [red]/ BLNO, 003007 [light blue]” (USNM). Paratype, same label information as male allotype (USNM: 1 male). Additional specimens examined: from same series as male allotype and paratype (USNM: 9 females, 8 males); “N. genus-N. sp., *Atriplex canescens* [non-focal], Presidio, T. 2376, Curculionidae, Det. L.L. Buchan.” (NMSU: 1 female); “TX: Presidio Co., Presidio, on *Atriplex canescens* [non-focal]” (NMSU: 1 female, 4 males); “TEX. Presidio Co., Presidio, 8-27-1971 [some with VIII-27-1971], C.W. O’Brien/ on *Larrea tridentata* [non-focal]” (CWOB: 8 females, 17 males; USNM: 1 female, 1 male); “Presidio Texas, 7-22-48 PresNo., 2376 JH Russel, *Atriplexcanescens* [non-focal], 48-16744” (USNM: 18 females, 24 males); “Presidio, Tex, X-1-43, Presidio 1142/ on *Atriplexcanescens* [non-focal]/ Lot. No. 43-11347” (USNM: 5 females, 4 males); “Presidio, Tex., IX-21-43, JH Russel/ on *Atriplexcanescens* [non-focal] foliage” (USNM: 1 female); “*Larrea divaricata* [non-focal]/ nr. Presdio, Tex. 34522, X-28-54-11853” (USNM: 4 females, 5 males).

#### Distribution.

This species has been found in Presidio, Texas (USA); and likely is also present in Chihuahua (Mexico) (Fig. 52).

#### Natural history.

Associated with creosote bush (*Larrea tridentata* [DC.] Coville [non-focal]; Zygophyllaceae [non-focal]) and four-wing saltbush (*Atriplex canescens* [Pursh] Nuttall. [non-focal]; Asteraceae [non-focal]). Erroneously listed as associated with *Larrea divaricata* Cav., which is not known to occur in this region.

### Minyomerus rutellirostris [JF2015]

Taxon classificationAnimaliaColeopteraCurculionidae

Jansen & Franz sec. Jansen & Franz (2015) sp. n.

http://zoobank.org/24BAD6A8-9225-47B5-9174-FDAB2B7E7A4A

Figs 46 , 47 , 48

#### Diagnosis.

*Minyomerus rutellirostris* [JF2015] can be readily differentiated from other congenerics by the separated procoxae and the size of the rostrum, which is as wide as the head, and distinctly square in dorsal view. In the males, the dilation of the rostrum is greatly exaggerated, and the lateral margins are flared. The ramus of the spermatheca is basally narrow, forming a stalk that tapers into an apical bulb. The aedeagus is elongate, narrowing towards the apexmore strongly in the region of the ostium.

#### Description – female.

*Habitus.* Length 4.47–5.29 mm, width 1.84–2.12 mm, length/width ratio 2.39–2.60, widest at anterior 1/4 of elytra. Integument orange-brown to black. Scales with variously interspersed colors ranging from slightly off-white to manila/tan to dark coffee brown, in some specimens appearing semi-translucent (in others opaque). Setae sub-recumbent, brown; with larger, more erect white setae interspersed throughout on even numbered elytral intervals, shorter setae arranged in rows on all elytral intervals.

*Mandibles.* Covered with opalescent scales, with 4 longer setae.

*Maxillae.* Cardo bifurcate at base with an inner angle of ca. 115°, inner (mesal) arm sub-equal in length to outer arm, inner arm of equal width to outer arm, arms of bifurcation equal in length to apically outcurved arm. Stipes sub-quadrate, nearly equilateral, roughly equal in length to inner arm of bifurcation of cardo, with a single lateral seta. Galeo-lacinial complexapically incurved (mesally); complexmembranous; setose in basal 2/3; dorsally with 8 apicomesal lacinial teeth; ventrally with 4 reduced lacinial teeth. Palpiger with a lateral patch of setae, sclerotized on basal 2/3.

*Maxillary palps.* Palpomeres I and II with apical ends facing mesally and forming a 45° angle with base, each with 2 apical setae; II with 1 mesoventral seta in addition to apical seta.

*Labium.* Prementum completely roughly hexagonal; apical margin straight, medially weakly or not projected (ligula reduced), weakly angulate; lateral margins slightly incurved; basal margin broadly arcuate. Labial palps 3-segmented, I with apexreaching margin of prementum; III slightly longer than II.

*Rostrum.* Length 0.57–0.77 mm, appearing distinctly flattened, anterior portion 2.5–3.0 × broader than long, rostrum/pronotum length ratio 0.46–0.53, rostrum length/width ratio 0.86–1.00; shape in cross section elongate rectangular. Dorsal outline of rostrum sub-rectangular, anterior half of dorsal surface slightly concave and weakly impressed, posterior half strongly rugose. Rostrum in lateral view rectangular; apical margin with 2 large vibrissae. Nasal plate defined by broad, Y-shaped concavities, convex, integument completely covered with scales similar to those of body. Margins of mandibular incision directed 30° outward dorsally in frontal view; ventrolateral sulci well defined, beginning as a sulcus dorsad of insertion point of mandibles, running parallel to scrobe, becoming fainter posteriorly and disappearing ventrally. Integument dorsad of sulcus expanded somewhat laterally. Dorsal surface of rostrum with median sulcus running from fovea at posterior end of anterior half rostrum to midpoint of posterior half of rostrum; ventrolateral margins sub-parallel, in some specimens diverging. Rostrum ventrally lacking foveae in line with insertion point of mandibles. Oral cavity with lateral margins feebly curved.

*Antennae.* Dorsal margin of scrobe overhangs forming a small tooth ventrad of anterior margin of eye. Funicular antennomeres evenly progressing from elongate to broader than long; terminal segment lacking appressed scales, having instead a covering of apically-directed pubescence with interspersed sub-erect setae. Club 2.5–3.0 × as long as wide.

*Head.* Eyes separated in dorsal view by 4–5 × their anterior-posterior length, touching anterior prothoracic margin. Head without transverse post-ocular impression.

*Pronotum.* Length/width ratio 0.94–0.99, sub-cylindrical to globular; widest near anterior 1/3; median sulcus absent. Anterior margin broadly arcuate, lateral margins curved and widening into a bulge at anterior 1/3 of pronotum, thence straight to posterior margin, posterior margin straight. Pronotum in lateral view setae that reach beyond anterior margin; these setae becoming evenly longer laterally, nearly reaching into anterior 1/2 of eye at their maximum length. Anterolateral margin with a reduced tuft of post-ocular vibrissae present, consisting of 4–6 setae, emerging near ventral margin of eye; vibrissae achieving a maximum length 3/4 × anterior-posterior length of eye.

*Pleurites.* Metepisternum covered by elytron near posterior 1/5 of metasternum.

*Abdominal sterna.* Procoxal cavities positioned at midpoint, contiguous, prosternal process complete between coxae, slightly elevated. Mesocoxal cavities separated by distance 1/2 × width of mesocoxal cavity. Metasternum without apprent transverse sulcus; metacoxal cavities widely separated by 3–4 × their width.

*Legs.* Profemur/pronotum length ratio 0.83–0.93; profemur with distal 1/5 produced ventrally as an oblique projection covering tibial joint. Protibia/profemur length ratio 0.87–0.94; mucro reduced to a small laterally projected tooth. Protarsus with tarsomere I 1.5 × as long as II; I and III similar in length, III equilateral. Metatibial apexwith almond shaped convex ity ringed by 11–13 short, widely separated, spiniform setae. *Elytra.* Length/width ratio 2.78–3.09; widest at anterior 1/4; anterior margins jointly 1.5 × wider than posterior margin of pronotum; lateral margins sub-parallel after anterior 1/4, more strongly rounded and converging in posterior 1/2. Elytra in lateral view sculpted with a shallow depression at anterior 1/3; posterior declivity angled at nearly 70° to main body axis. Elytral striae defined; punctures visible, separated by 3–5 × their diameter; intervals elevated, with every other interval slightly more raised and with interspersed, sub-erect, white setae.

*Abdominal sterna.* Ventrite III elevated and set off from IV along lateral 1/4 of its length. Sternum VII mesally 3/5 × as long as wide; anterior margin weakly curved.

*Tergum.* Pygidium (tergum VIII) sub-conical; medial 1/3 of anterior 2/3 of pygidium less sclerotized.

*Sternum VIII.* Anterior laminar edges each incurved forming a 110° angle with lateral margin; less sclerotized medially.

*Ovipositor.* Coxites slightly sclerotized, in dorsal view 2 × as long as broad.

*Spermatheca.* ?-shaped; collum short, apically with a small, hood-shaped projection angled at 90° to ramus, nearly equal in length to stalk of ramus and contiously aligned with curvature of bulb of ramus; collum sub-contiguous with, and angled at 90° to ramus; ramus basally elongate, forming a stalk, equal in length to collum, bulbous apically, 3 × thicker than stalk; corpus not swollen, of equal thickness to collum and cornu; cornu elongate, apically, gradually narrowed, strongly recurved in basal 1/4, straight along mesal 1/2, and curved near apical 1/4 such that apexis parallel to collum and corpus.

**Figure 46.**
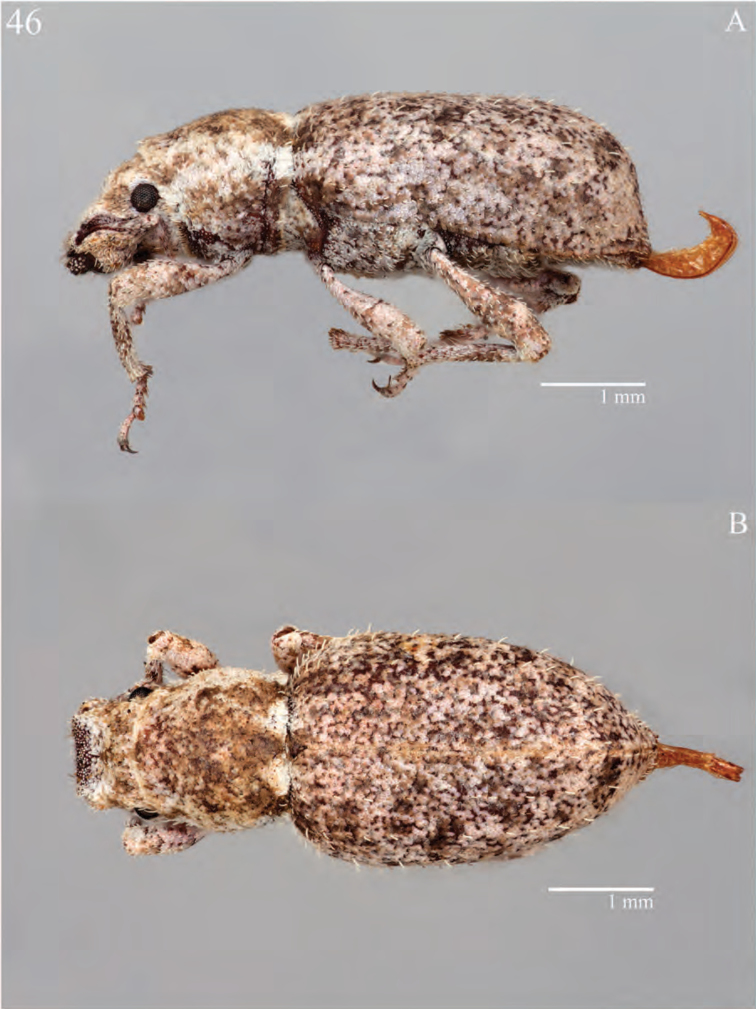
Habitus of *Minyomerus rutellirostris* [JF2015], female **A** lateral view **B** dorsal view.

**Figure 47.**
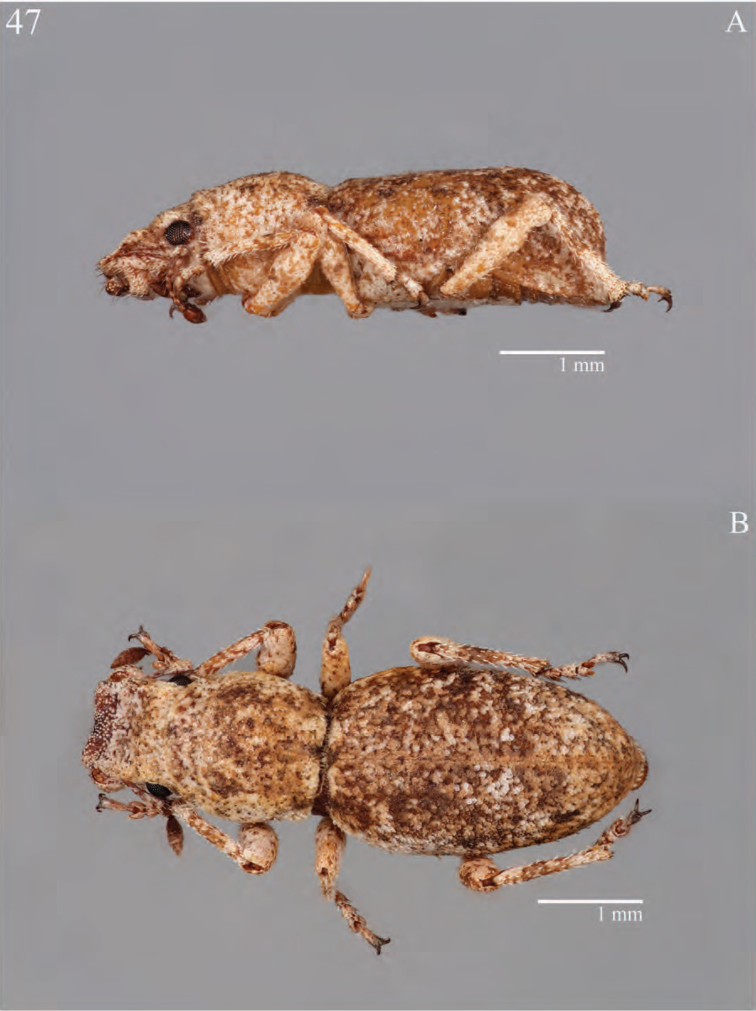
Habitus of *Minyomerus rutellirostris* [JF2015], male **A** lateral view **B** dorsal view.

**Figure 48.**
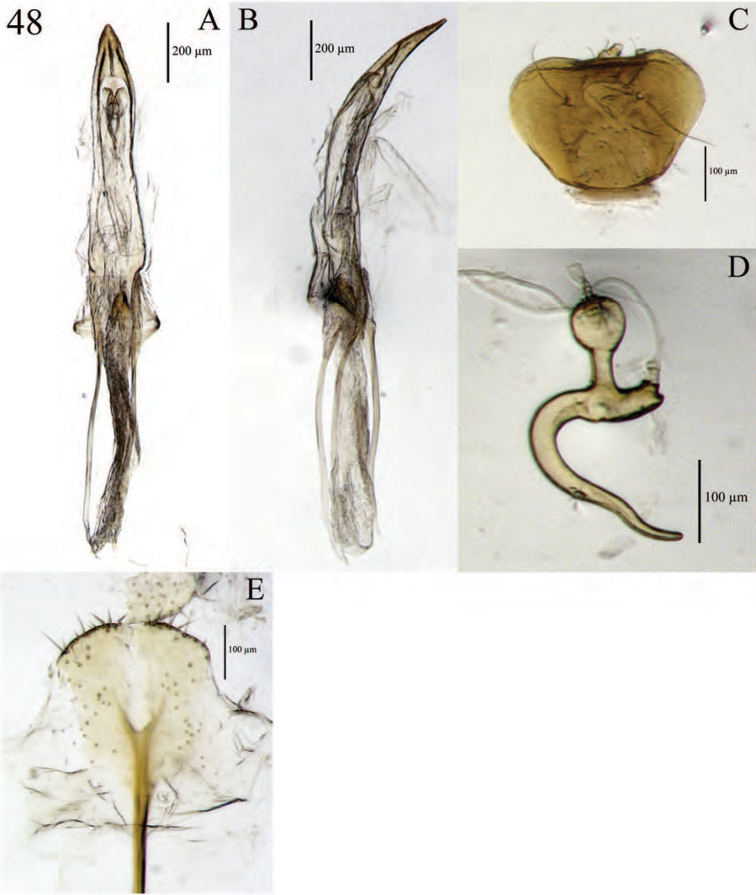
Diagnostic features and terminalia of *Minyomerus rutellirostris* [JF2015] **A** aedeagus, dorsal view **B** aedeagus, lateral view **C** labial prementum, ventral view **D** spermatheca **E** lamina of sternum VIII.

#### Male.

Similar to female, except where noted. Length 4.03–5.28 mm, width 1.36–1.80 mm, length/width ratio 2.70–3.17. Rostrum length 0.63–0.78 mm, rostrum/pronotum length ratio 0.49–0.55, rostrum length/width ratio 0.90–0.95. Pronotum length/width ratio 0.98–1.05. Profemur/pronotum length ratio 0.82–0.91, protibia/profemur length ratio 0.87–0.97. Elytra length/width ratio 3.01–3.21.

*Rostrum.* Rostrum similar to female, but generally more sculpted. Rostrum dorsally significantly more concave, emarginate, and with margins flared outwardly. Ventrolateral margins expanded laterally, each appearing as a semicircular projection beneath insertion point of scrobe. Scrobe channel less curved than female. Dorso-ventrally thinner than female.

*Head.* More sculpted than female, and with a more pronounced post-ocular impression. Thorax. More globular than female, but otherwise similar.

*Elytra.* In lateral view, dorsum flat, declivity significantly sharper, angled at 90° to main body axis. Intervals raised and all of uniform height and setation; lacking any of larger, more erect setae found on female.

*Abdomen.* Sternum VII more broadly arcuate posteriorly, 1/2 as long as wide. Tergum VII with posterior margin straight. Pygidium (tergum VIII) with posterior 1/2 punctate; anterior 1/2 rugose. Posterior 1/4 constricted and depressed, with posterior margin flaring out and slightly projected dorsally.

*Sternum VIII.* Consisting of 2 sub-contiguous, sub-triangular sclerites; posterior margins widely angulate. Laminar alae of spiculum gastrale lateral with lateral margins basally parallel, apically outcurved.

*Aedeagus.* Length/width ratio 4.36–4.73; lateral margins converging posteriorly, somewhat more strongly converging in region of ostium. Width of pedon in lateral view becoming gradually narrower posteriorly in anterior 1/2, ventral margins in posterior 1/2 becoming straight towards apex, then abruptly curving to meet dorsal margins at a sharp apical point; apexacutely angulate. Flagellum apically with a small conical sclerite.

#### Etymology.

Named in reference to the dilated, concave rostrum of the male, which has the appearance of a shovel; *rutellum* = shovel, *rostrum* = beak or nose, *rutellirostris* = with a shovel-nose; Latin adjective (Brown 1956).

#### Material examined.

Holotype – female “MEXICO: BAJA CALI–, FORNIA SUR: 3 km N., San Ignacio at km 77, 5-I-1979, Stop 79-33, Calif. Acad. Sci. Coll./ COLLECTORS:, David Weissman, Robert Love, Vincent Lee, Carolyn Mullinex” (CAS). Paratypes – same label information as female holotype (CAS: 1 female); “La Paz, L. Cal., X-7-41/ Ross & Bohart, Collectors” (CAS: 1 male); “San Pedro, L. Cal. X-7-41/ Ross & Bohart, Collectors/ *Elissa* sp. # 1” (CAS: 3 females, 5 males); “MEX: 12 mi. NW La Paz, Baja Calif. IX-18-67/ J. Chemsak, A. & M. Michelbache, Collectors” (EMEC: 1 female, 1 male); “MEX: Baja Sur, 8 mi. S. Mulege, X-7-1983 BLACKLIGHT, Faulkner & Andrews/ *Minyomerus* sp.” (CSCA: 2 females); “MEX: Baja Calif. Sur, 34.4 mi. SE. Guerrero, Negro IX-22-1981, F. Andrews & D. Faulkner/ Collected in, Cereal bowl, Pit trap” (CSCA: 1 female); “Purissima, Oct. ‘73, Baja Calif., WM Mann. *Minyomerus* sp., DET. A T HOWDEN” (USNM: 1 male).

#### Distribution.

This species is found in Baja California Sur (Mexico) (Fig. 52).

#### Natural history.

Host plant unknown, though possibly creosote bush (*Larrea tridentata* [DC.] Coville [non-focal]; Zygophyllaceae [non-focal]). No host plant associations have been documented.

### Identification key to the species of *Minyomerus* [JF2015]

**Table:** 

1	Procoxae apparently separate, with intercoxal processes touching or very nearly so	**2**
–	Procoxae apparently contiguous, with intercoxal processes short and not touching	**3**
2(1)	Rostrum approximately square and as wide as head in dorsal view; ramus of spermatheca basally narrow, forming a stalk that tapers into an apical bulb	***Minyomerus rutellirostris* [JF2015], sp. n.**
–	Rostrum approximately trapezoidal and narrower than the head in dorsal view; ramus of spermatheca cylindrical, somewhat bulbous, and basally constricted	***Minyomerus griseus* (Sleeper) [JF2015]**
3(1)	Anterior margin of pronotum bearing a full, well-developed tuft of 10 or more ocular vibrissae; anterolateral margins of prementum explanate, angular, and posteriorly declivous, with a distinctly hexagonal appearance	**4**
–	Ocular vibrissae reduced in number or length; anterior margins of prementum not explanate and declivous, typically with a pentagonal appearance	**5**
4(3)	Head very wide and only somewhat swollen between eyes; rostrum ca. 4× wider than long in dorsal view; pronotum in dorsal view cylindrical; elytral setae short, brown, and sub-recumbent; ramus of spermatheca stalked and with apical bulb abruptly constricted, not tapering at point of connection to stalk	***Minyomerus laticeps* (Casey) [JF2015]**
–	Head and rostrum typical (rostrum 2-3× wider than long in dorsal view); pronotum in dorsal view somewhat globular, with a strong anterior constriction; elytral setae short and setiform, especially near disk; spermatheca without basal stalk	***Minyomerus constrictus* (Casey) [JF2015]**
5(3)	Metatibial apexstrongly convex, with setae similar in length to those of remainder of leg, somewhat lighter in color and translucent, and slightly lamelliform; head somewhat conical in form, rounded between the eyes; elytral setae copious, not in uniform rows on intervals, instead appearing in offset rows, especially near elytral suture and declivity	**6**
–	Metatibial apexoblique or weakly convex, with setae short and conical in appearance; head roughly quadrate; elytral setae in relatively uniform rows on elytra, not strongly offset	**7**
6(5)	Elytral striae deeply and distinctly punctate, appearing pin-striped; elytra without obvious humeri, gradually widening posteriorly; ramus of spermatheca elongate, annulate, and sub-apically situated on corpus	***Minyomerus aeriballux* [JF2015], sp. n.**
–	Elytral striae punctate, with punctures somewhat obscured by appressed scales; elytra somewhat pyriform, with weak, but obviously present humeri; ramus of spermatheca elongate, somewhat swollen, and sub-apically situated on corpus	***Minyomerus reburrus* JF2015], sp. n.**
7(5)	Elytra very strongly convex in lateral view; anterior margin of pronotum wider than posterior margin; spermatheca comma-shaped, with the ramus reduced, apically flattened and sub-contiguous with the collum; aedeagal pedon membranous ventrally, and not fully sclerotized	***Minyomerus conicollis* Green [JF2015]**
–	Elytra only somewhat convex to nearly flat in lateral view; anterior margin of pronotum similar in length to posterior margin; spermatheca variable; aedeagal pedon fully sclerotized	**8**
8(7)	Elytral setae a mix of shorter, brown setae and longer, more erect, white setae	**9**
–	Elytral setae uniform	**10**
9(8)	Setae apically explanate, appearing somewhat spatulate; corpus of spermatheca uniquely elongate	***Minyomerus caseyi* (Sharp) [JF2015]**
–	Setae linear; corpus of spermatheca typical, ramus bulbous and basally constricted	***Minyomerus trisetosus* [JF2015], sp. n.**
10(8)	Anterior margin of pronotum lined with linear setae that extend anteriorly beyond margin by half their length	**11**
–	Anterior margin of pronotum lacking setae, or with setae that do not extend far beyond margin	**12**
11(10)	Lateral margins of gular cavity strongly rounded, never straight, and slightly longer than posterior margin; frons weakly projected between eyes; appressed scales on elytra without opalescent sheen; nasal plate with or without metallic reflections; lamina of spiculum ventrale sclerotized throughout	***Minyomerus languidus* Horn [JF2015]**
–	Lateral margins of gular cavity nearly straight, and not longer than posterior margin; frons strongly projected between eyes; appressed scales with strong opalescent sheen; nasal plate with metallic reflections; lamina of spiculum ventrale with a membranous region present medially between laminar arm	***Minyomerus gravivultus* [JF2015], sp. n.**
12(10)	Elytra each 4-5× as long as broad in dorsal view, strongly punctate; elytra constricted anteriad of humeri, narrower than the pronotum, widening thereafter near the humeri; spermatheca with the corpus somewhat bulbous, and the ramus either flattened somewhat or slightly elongate	***Minyomerus cracens*[JF2015], sp. n.**
–	Elytra not so elongate, variably punctate; elytra lacking basal constriction; spermatheca variable	**13**
13(12)	Elytral striae with punctures	**14**
–	Elytral striae without punctures	**15**
14(13)	Frons strongly protuberant; elytra in lateral view convex dorsally; spermatheca with corpus possessing an annulate, rectate projection nearly 1/2× length of ramus; aedeagal pedon evenly curving towards apex; aedeagal flagellum with spiriform apical sclerite that spirals counterclockwise and of equal length to aedeagal pedon	***Minyomerus bulbifrons* [JF2015], sp. n.**
–	Frons not so protuberant; elytra in lateral view nearly flat dorsally; spermatheca with corpus possessing an annulate, rectate projection nearly 2/3× length of the ramus; aedeagal pedon narrow and elongate; aedeagal flagellum with very minute apical sclerite	***Minyomerus puticulatus* [JF2015], sp. n.**
15(13)	Elytra with intervals not elevated or sculpted, apparently smooth; body not especially robust in overall quality; appressed scales bluish-gray to white, appearing smooth, never ‘crusty’; spermathecal with ramus and collum not appearing as subcontinuous, apically invaginated bulbs	**16**
–	Intervals broadly sculpted and raised, and striae not punctate; body generally robust in overall quality; appressed scales uniformly beige and gray, with a distinctly ‘crusty’ appearance; spematheca with ramus and collum appearing as two subcontiguous, apically invaginated bulbs	***Minyomerus microps* (Say) [JF2015]**
16(15)	Head nearly flat between eyes; nasal plate very weakly impressed; elytra with very minute setae, only perceptible at high magnification; ramus of spermatheca elongate, cylindrical, and slightly thinner than corpus, and cornu strongly recurved in basal half with uniquely sinuate appearance; males not known	***Minyomerus imberbus* [JF2015], sp. n.**
–	Frons strongly protruding between eyes; nasal plate deeply impressed; elytra with small, but easily visible setae; spermatheca with the ramus elongate and apically swollen, corpus possessing an annulate, rectate projection nearly 1/2× length of the ramus, and cornu evenly recurved throughout its length; aedeagal flagellum with a spiriform apical sclerite that spirals clockwise and of equal length to aedeagal pedon	***Minyomerus politus* [JF2015], sp. n.**

### Phylogenetic analysis of the species of *Minyomerus* [JF2015]

A matrix of 46 characters was assembled for the 22 terminal taxa (Table 1). Cladistic parsimony analysis returned a single, most-parsimonious cladogram with a length (L) of 82 steps, a consistency index (CI) of 65 and a retention index (RI) of 82 (Farris 1989). TNT was used to confirm that the shortest tree had been found (Goloboff et al. 2008). The assessed characters, states, and preferred optimizations will be jointly treated in this section. Due to a lack of prior study establishing generic relationships with Tanymecini, only characters relevant to the delimitation and internal relationships of *Minyomerus* [JF2015] are discussed in detail. For all characters not resolved as unreversed synapomorphies, both the individual consistency (ci) and retention (ri) indices are provided. Characters are numbered in accordance with descriptive sequence used in the genus/species accounts. A “–” symbol indicates inapplicable (character, state), whereas a “?” symbol indicates missing information, e.g., due to the unavailability of male specimens or insufficient specimens on hand to permit full dissections. At the reviewers’ request, we explored adding three outgroup representatives (one naupactine [non-focal], two tanymecines [non-focal]) to reaffirm that the polarity of characters of the male and female terminalia among the ingroup taxa was robustly inferred. Characters 7, 33, 38, 39, 41, and 46 were mapped onto the preferred phylogeny using ACCTRAN optimization, and the remaining characters had an unambiguous optimization.

**Table 1.** Taxon/character matrix used for for cladistics analysis of the species of *Minyomerus* [JF2015] and select outgroup taxa. All multistate characters coded as additive, except for character 33. The symbol “–” denotes inapplicable character states, whereas “?” denotes missing information (see also text).

Taxon/character	0 1 1 2 2 3 3 4 4
	5 0 5 0 5 0 5 0 5
*Sitona californicus* [non-focal]	000?? ???00 00000 00000 00000 00--- ----- -???? ????? ?
*Pandeleteius cinereus* [non-focal]	110?? ???01 00000 01100 00010 00000 00-00 0???? ????? ?
*Pandeleteinus subcancer* [non-focal]	110?? ???01 00000 01101 00010 00000 00-00 0???? ????? ?
*Isodrusus debilis* [non-focal]	110?? ???01 00000 01101 10010 00000 00-00 0???? ????? ?
*Isodacrys buchanani* [non-focal]	110?? ???01 00000 01101 10010 10000 00-00 0???? ????? ?
*Minyomerus imberbus* [JF2005]	210?? ???02 01010 02001 11121 10000 00000 0???? ????? ?
*Minyomerus constrictus* [JF2005]	21000 01002 11010 02001 11121 10000 00000 00000 10100 1
*Minyomerus laticeps* [JF2005]	21000 01002 11010 02001 11121 10000 00000 00000 10100 1
*Minyomerus conicollis* [JF2005]	21000 00002 01010 02001 11021 10001 10000 10000 00000 1
*Minyomerus languidus* [JF2005]	21011 10002 01010 02001 11021 10001 10000 ????? ????? ?
*Minyomerus microps* [JF2005]	21011 10102 11010 02001 11021 10001 10001 0???? ????? ?
*Minyomerus aeriballux* [JF2005]	22011 10112 02010 12001 12021 10001 10011 00000 00000 0
*Minyomerus reburrus* [JF2005]	22011 10112 02011 12001 12021 10001 10210 0???? ????? ?
*Minyomerus cracens* [JF2005]	21011 10102 02011 12001 11121 10001 10010 00001 00101 1
*Minyomerus caseyi* [JF2005]	21100 10102 02011 12001 11121 10001 11011 00101 00100 0
*Minyomerus trisetosus* [JF2005]	21100 10102 02010 12001 11021 10001 11011 0???? ????? ?
*Minyomerus puticulatus* [JF2005]	21011 10102 02010 12001 11121 11101 10100 10110 10001 0
*Minyomerus bulbifrons* [JF2005]	21011 10102 02111 12001 11021 11101 10000 11100 10010 0
*Minyomerus politus* [JF2005]	210?? ???02 02111 12001 11021 11101 10100 11110 10011 0
*Minyomerus gravivultus* [JF2005]	21011 10102 12010 02001 11021 10111 1000? ?0100 00001 0
*Minyomerus griseus* [JF2005]	21010 10102 12010 02011 11121 10111 10000 00100 11001 1
*Minyomerus rutellirostris* [JF2005]	21110 10102 12010 02011 11121 10111 10000 00100 11001 1

1. Habitus, form of appressed scales: (0) elongate pyriform, not overlapping; (1) sub-circular to polygonal, variously overlapping non-linearly; (2) sub-circular and only overlapping posteriorly. Coded as additive due to alignment of character states with the preferred phylogeny. Coding as non-additive in isolation or in unison with other additive multistate characters does not affect polarization of the character/states or alter the phylogeny. State 1 is a synapomorphy for the tanymecine clade [non-focal], whereas state 2 is a synapomorphy for *Minyomerus* [JF2015].
2. Habitus, arrangement of elytral setae: (0) variously interspersed; (1) arranged in single-file rows on elytral intervals; (2) arranged non-uniformly on elytral intervals. Coded as additive due to alignment of character states with the preferred phylogeny. Coding as non-additive in isolation or in unison with other additive multistate characters does not affect polarization of the character/states or alter the phylogeny. State 1 is a synapomorphy for the tanymecine clade [non-focal], whereas state 2 is a synapomorphy the *Minyomerus aeriballux*–*Minyomerus reburrus* clade [JF2015].
3. Habitus, rows of elytral setae with larger white setae interspersed among smaller brown setae: (0) absent; (1) present. Convergently present in the *Minyomerus caseyi*–*Minyomerus trisetosus* clade [JF2015] and in *Minyomerus rutellirostris* [JF2015] (ci = 50; ri = 50).
4. Prementum, anterior margin forming a distinct face that continues to lateral margins: (0) absent; (1) present. Synapomorphy for the *Minyomerus languidus*–*Minyomerus griseus* clade, with a single reversal in the *Minyomerus caseyi*–*Minyomerus trisetosus* clade (ci = 50; ri = 75).
5. Prementum, strongly ligulate and with margins nearly straight, appearing pentagonal: (0) absent; (1) present. Synapomorphy for the *Minyomerus languidus*–*Minyomerus rutellirostris* clade [JF2015], with independent reversals in the *Minyomerus caseyi*–*Minyomerus trisetosus* clade [JF2015] and *Minyomerus griseus*–*Minyomerus rutellirostris* clade [JF2015], respectively (ci = 33; ri = 60).
6. Prementum, anterolateral margins simple, unexpanded: (0) absent; (1) present. Synapomorphy for the *Minyomerus languidus*–*Minyomerus rutellirostris* clade [JF2015].
7. Prementum, anterolateral margins explanate, angular, and posteriorly declivous, with a distinctly hexagonal appearance: (0) absent; (1) present. ACCTRAN optimization preferred (see Agnarsson and Miller 2008), therefore inferred as a synapomorphy for the *Minyomerus imberbus*–*Minyomerus laticeps* clade [JF2015].
8. Prementum, exposure of palpomere I: (0) exposed, visible beyond ligula and anterior margin of prementum in ventral view; (1) hidden, fully covered or only minutely exposed beyond ligula and anterior margin of prementum in ventral view. Synapomorphy for the *Minyomerus microps*–*Minyomerus rutellirostris* clade [JF2015].
9. Rostrum, form in dorsal view: (0) approximately quadrate; (1) somewhat conical, medially convex. Synapomorphy for the *Minyomerus aeriballux*–*Minyomerus reburrus* clade.
10. Rostrum, form of nasal plate and demarcation of epistoma: (0) with three parallel, longitudinal carinae, and surface planar between these; (1) with a sharp, narrow, chevron-shaped carina demarcating epistoma; (2) with a broad, scale-covered, chevron-shaped carina demarcating epistoma. Coded as additive due to alignment of character states with preferred phylogeny. Coding as non-additive in isolation or in unison with other additive multistate characters does not affect polarization of the character/states or alter the phylogeny. State 1 is a synapomorphy for the tanymecine clade [non-focal], whereas state 2 is a synapomorphy for *Minyomerus* [JF2015].
11. Rostrum, sulcus posteriad of nasal plate weakly impressed: (0) absent; (1) present. Convergently present in the *Minyomerus constrictus*–*Minyomerus laticeps* clade [JF2015], *Minyomerus microps* [JF2015], and the *Minyomerus gravivultus*–*Minyomerus rutellirostris* clade [JF2015] (ci = 33; ri = 60).
12. Rostrum, form of sulcus posteriad of nasal plate: (0) absent; (1) sulcus present, broad, and weakly punctate; (2) sulcus present, more strongly punctate. Coded as additive due to alignment of character states with preferred phylogeny. Coding as non-additive in isolation or in unison with other additive multistate characters does not affect polarization of the character/states or alter the phylogeny. Synapomorphy for *Minyomerus* [JF2015] (state 1) and the *Minyomerus aeriballux*–*Minyomerus rutellirostris* clade [JF2015] (state 2), respectively.
13. Head, frons very strongly projected beyond anterior margin of eye, by 2 × anterior-posterior length of eye: (0) absent; (1) present. Synapomorphy for the *Minyomerus bulbifrons*–*Minyomerus politus* clade [JF2015].
14. Antenna, length of scrobe relative to funicle and club: (0) scrobe shorter than funicle and club combined; (1) scrobe subequal in length to funicle and club combined. Synapomorphy for *Minyomerus* [JF2015].
15. Antenna, terminal funicular segment entirely without thin, nearly setiform scales: (0) absent; (1) present. Convergently present in the *Minyomerus aeriballux*–*Minyomerus trisetosus* clade [JF2015] and the *Minyomerus bulbifrons*–*Minyomerus politus* clade [JF2015], with independent reversals in *Minyomerus aeriballux* [JF2015] and *Minyomerus trisetosus* [JF2015] (ci = 25; ri = 25).
16. Antenna, terminal funicular segment at least partially clothed with broad scales: (0) absent; (1) present. Synapomorphy for the *Minyomerus aeriballux*–*Minyomerus rutellirostris* clade [JF2015] with a reversal in the *Minyomerus gravivultus*–*Minyomerus rutellirostris* clade [JF2015] (ci = 50; ri = 85).
17. Head, angle of base in relation to prothorax: (0) directed anteriorly, in line with main body axis; (1) directed strongly ventrally; (2) directed slightly ventrally. Coded as additive due to alignment of character states with preferred phylogeny. Coding as non-additive in isolation or in unison with other additive multistate characters does not affect polarization of the character/states or alter the phylogeny. State 1 is a synapomorphy for the tanymecine clade [non-focal], whereas state 2 is a synapomorphy for *Minyomerus* [JF2015].
18. Prosternum, intercoxal process complete, undivided: (0) absent; (1) present. Synapomorphy for the tanymecine clade [non-focal], with a single reversal for *Minyomerus* [JF2015] (ci = 50; ri = 66).
19. Prosternum, intercoxal process divided at midpoint between coxae, but both anterior and posterior processes extending completely between procoxae and contiguous with each other: (0) absent; (1) present. Synapomorphy for the *Minyomerus griseus*–*Minyomerus rutellirostris* clade [JF2015].
20. Legs, fore femora not swollen in comparison to other legs: (0) absent; (1) present. Synapomorphy for the *Pandeleteinus subcancer*–*Minyomerus rutellirostris* clade [non-focal].
21. Legs, sculpture of ventral surface of protibiae: (0) evenly convex throughout; (1) with a longitudinal groove or concavity. Synapomorphy for the *Isodrusus debilis*–*Minyomerus rutellirostris* clade [JF2015].
22. Legs, setation of metatibial apex: (0) bristles at least as long as surrounding setae and setiform; (1) bristles shorter than surrounding setae and conical; (2) bristles subequal in length to surrounding setae and somewhat lamelliform. Coded as additive due to alignment of character states with preferred phylogeny, and the appearance of being a transformation series. Coding as non-additive in isolation or in unison with other additive multistate characters does not affect polarization of the character/states or alter the phylogeny. Synapomorphy for *Minyomerus* [JF2015] (state 1) and the *Minyomerus aeriballux*–*Minyomerus reburrus* clade [JF2015] (state 2), respectively.
23. Legs, curvature of metatibial apex: (0) convex; (1) oblique. Convergent gains (state 1) in the *Minyomerus imberbus*–*Minyomerus laticeps* clade [JF2015], the *Minyomerus cracens*–*Minyomerus trisetosus* clade [JF2015], *Minyomerus puticulatus* [JF2015], and the *Minyomerus griseus*–*Minyomerus rutellirostris* clade [JF2015], with a single reversal in *Minyomerus trisetosus* [JF2015] (ci = 20; ri = 42).
24. Legs, relative length of mesotarsi to mesotibiae: (0) tarsi much shorter than tibiae; (1) tarsi at least equal in length to tibiae; (2) tarsi slightly shorter than tibiae. Coded as additive due to alignment of character states with preferred phylogeny. Coding as non-additive in isolation or in unison with other additive multistate characters does not affect polarization of the character/states or alter the phylogeny. State 1 is a synapomorphy for the tanymecine clade [non-focal], whereas state 2 is a synapomorphy for *Minyomerus* [JF2015].
25. Legs, tarsi ventrally spinose: (0) absent; (1) present. Synapomorphy for *Minyomerus* [JF2015].
26. Elytra, humeral angle rounded, not projected: (0) absent; (1) present. Synapomorphy for the *Isodacrys buchanani*–*Minyomerus rutellirostris* clade [JF2015].
27. Female terminalia, spermatheca with apical cylindrical bulb on corpus: (0) absent; (1) present. Synapomorphy for the *Minyomerus puticulatus*–*Minyomerus politus* clade [JF2015].
28. Female terminalia, lamina of spiculum ventrale less sclerotized between laminar arms: (0) absent; (1) present. Coded as inapplicable for *Sitona californicus* [non-focal], as laminar arms are not apparent. Synapomorphy for the *Minyomerus puticulatus*–*Minyomerus rutellirostris* clade [JF2015].
29. Female terminalia, lamina of spiculum ventrale with laminar arms bifurcating around a membranous region: (0) absent; (1) present. Coded as inapplicable for *Sitona californicus* [non-focal], as laminar arms are not apparent. Synapomorphy for the *Minyomerus gravivultus*–*Minyomerus rutellirostris* clade [JF2015].
30. Female terminalia, lamina of spiculum ventrale with style basally divided or obscured, not mesally intact: (0) absent; (1) present. Coded as inapplicable for *Sitona californicus* [non-focal], as laminar arms are not apparent. Synapomorphy for the *Minyomerus conicollis*–*Minyomerus rutellirostris* clade [JF2015].
31. Female terminalia, lamina of spiculum ventrale with laminar arms clearly bifurcating. (0) absent; (1) present. Coded as inapplicable for *Sitona californicus* [non-focal], as laminar arms are not apparent. Synapomorphy for the *Minyomerus conicollis*–*Minyomerus rutellirostris* clade [JF2015].
32. Female terminalia, coxites of ovipositor with a lateral, anteriorly-directed, recurved, alate process: (0) absent; (1) present. Coded as inapplicable for *Sitona californicus* [non-focal], as coxites of ovipositor are not apparent. Synapomorphy for the *Minyomerus caseyi*–*Minyomerus trisetosus* clade [JF2015].
33. Female terminalia, relative length of styli to coxites of ovipositor: (0) Similar in size; (1) distinctly shortened; (2) highly reduced, appearing minute. Coded as non-additive, due to strong differences in structure of coxites and styli in state 2; inapplicable for outgroup taxa, as styli of ovipositor are not apparent. ACCTRAN optimization preferred (see Agnarsson and Miller 2008), therefore inferred as a synapomorphy for the *Minyomerus puticulatus*–*Minyomerus politus* clade [JF2015] (state 1), with a single reversal in *Minyomerus bulbifrons* [JF2015] (state 0). Autapomorphy for *Minyomerus reburrus* [JF2015] (state 2).
34. Female terminalia, condition of medial, anteriorly-directed, sclerotized process of coxites of ovipositor: (0) fully developed; (1) reduced and inapparent. Coded as inapplicable for *Sitona californicus* [non-focal], as coxites of ovipositor are not apparent. Synapomorphy for the *Minyomerus aeriballux*–*Minyomerus trisetosus* clade [JF2015].
35. Female terminalia, anterior margin of tergum VII entirely free of sclerotized band: (0) absent; (1) present. Coded as inapplicable for *Sitona californicus* [non-focal], as the tergum VII is evenly sclerotized throughout. Convergently present in *Minyomerus microps* [JF2015], *Minyomerus aeriballux* [JF2015], and the *Minyomerus caseyi*–*Minyomerus trisetosus* clade [JF2015] (ci = 33; ri = 33).
36. Female terminalia, anterior margin of tergum VII sclerotized fully, appearing as an obviously complete band: (0) absent; (1) present. Coded as inapplicable for *Sitona californicus* [non-focal], as the tergum VII is evenly sclerotized throughout. Convergently present in *Minyomerus conicollis* [JF2015] and the *Minyomerus puticulatus*–*Minyomerus politus* clade [JF2015] (ci = 50; ri = 66).
37. Male terminalia, apical sclerite of aedeagal flagellum elongate-spiriform: (0) absent; (1) present. Synapomorphy for the *Minyomerus bulbifrons*–*Minyomerus politus* clade [JF2015].
38. Male terminalia, style of spiculum gastrale with an anterior ventral flange: (0) absent; (1) present. ACCTRAN optimization preferred (see Agnarsson and Miller 2008), therefore convergently present in *Minyomerus caseyi*–*Minyomerus trisetosus* clade [JF2015] and the *Minyomerus bulbifrons*–*Minyomerus politus* clade [JF2015] (ci = 50; ri = 75).
39. Male terminalia, lamina of spiculum gastrale longer than broad and anteriorly extended along syle: (0) absent; (1) present. ACCTRAN optimization preferred (see Agnarsson and Miller 2008), therefore inferred as a synapomorphy of the *Minyomerus puticulatus*–*Minyomerus politus* clade [JF2015], with a reversal in *Minyomerus bulbifrons* [JF2015] (ci = 50; ri = 0).
40. Male terminalia, sub-triangular sclerites of sternum VIII with a medial process: (0) absent; (1) present. Synapomorphy for the *Minyomerus cracens*–*Minyomerus trisetosus* clade [JF2015].
41. Male terminalia, curvature of posterior margin of tergum VII: (0) evenly arcuate; (1) medially incurved. ACCTRAN optimization preferred, therefore convergently present in the *Minyomerus imberbus*–*Minyomerus laticeps* clade [JF2015] and the *Minyomerus puticulatus*–*Minyomerus rutellirostris* clade [JF2015] with a reversal in *Minyomerus gravivultus* [JF2015] (ci = 33; ri = 50).
42. Male terminalia, tergum VII approximately 4 × as long as broad: (0) absent; (1) present. Synapomorphy for the *Minyomerus griseus*–*Minyomerus rutellirostris* clade [JF2015].
43. Male terminalia, aedeagal pedon expanded laterally around ostium: (0) absent; (1) present. ACCTRAN optimization preferred (see Agnarsson and Miller 2008), therefore convergently present in the *Minyomerus imberbus*–*Minyomerus laticeps* clade [JF2015] and the *Minyomerus caseyi*–*Minyomerus trisetosus* clade [JF2015] (ci = 50; ri = 66).
44. Male terminalia, aedeagal pedon broad basally, evenly tapering toward apex: (0) absent; (1) present. Synapomorphy for the *Minyomerus bulbifrons*–*Minyomerus politus* clade [JF2015].
45. Male terminalia, aedeagal pedon medially sclerotized along dorsum: (0) absent; (1) present. Convergently present in the *Minyomerus cracens* [JF2015] and the *Minyomerus puticulatus*–*Minyomerus rutellirostris* clade [JF2015], with a reversal in *Minyomerus bulbifrons* [JF2015] (ci = 33; ri = 60).
46. Male terminalia, width of connection between apodemes of aedeagal tegmen: (0) narrower than base of apodeme; (1) wider than base of apodeme. ACCTRAN optimization preferred (see Agnarsson and Miller 2008), therefore inferred as a synapomorphy for *Minyomerus languidus*–*Minyomerus rutellirostris* clade [JF2015] (state 0), with reversals in *Minyomerus cracens* [JF2015] and the *Minyomerus griseus*–*Minyomerus rutellirostris* clade [JF2015] (ci = 33; ri = 60).

## Discussion

**Figure 49.**
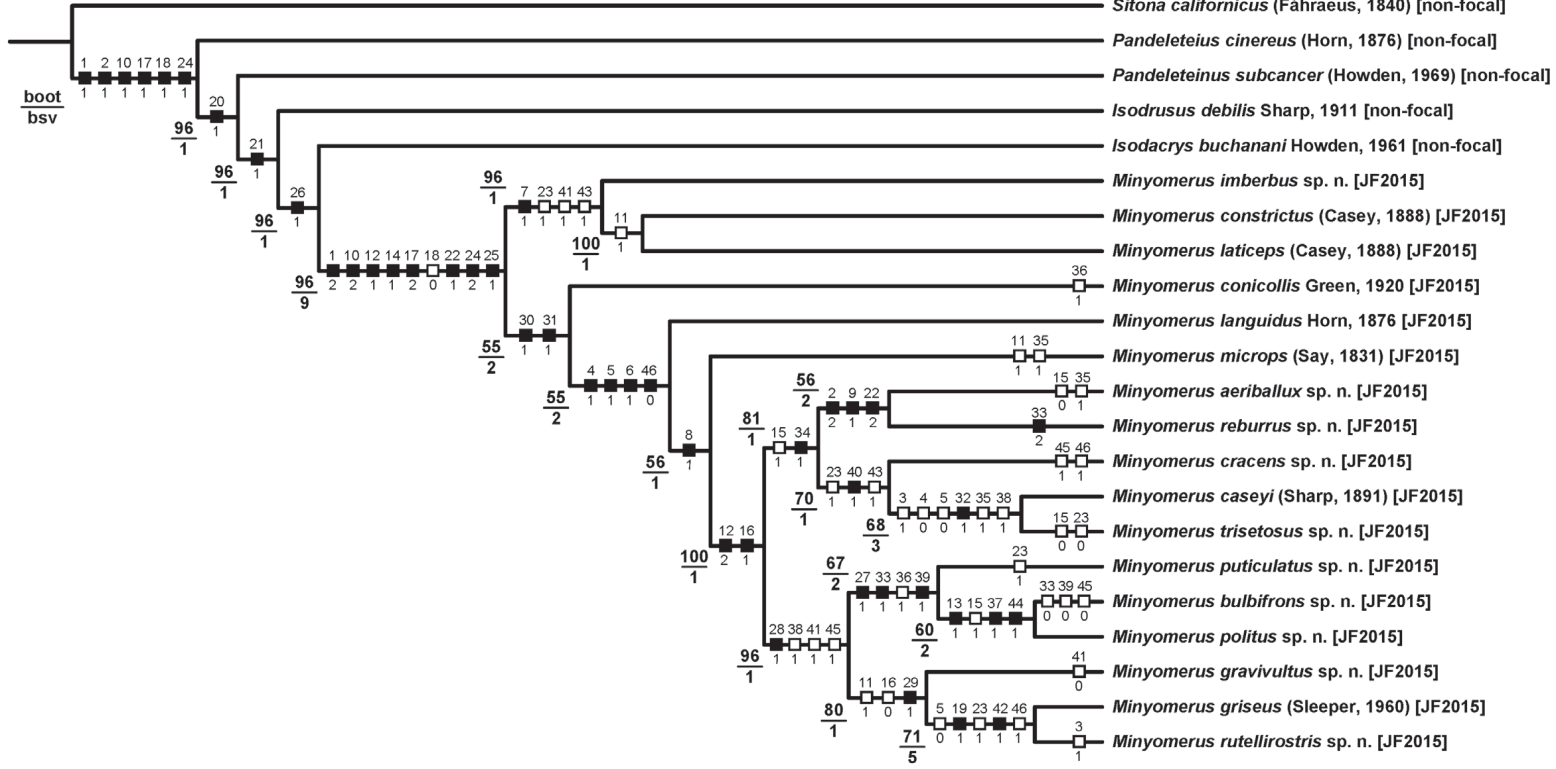
Single most parsimonious cladogram representing the preferred phylogeny of species of *Minyomerus* [JF2015], and select outgroup taxa (L = 82, CI = 65, RI = 82). Characters 7, 33, 38, 39, 41, 43, and 46 are mapped under ACCTRAN optimization; all others are unambiguously optimized. Black squares indicate non-homoplasious character state changes, whereas white squares indicate homoplasious character state changes. The numbers above and below the squares represent character numbers and states, respectively. Bootstrap (upper value) and Bremer support (lower value) values can be found at the left ends of the branches.

**Monophyly and phylogenetic placement of *Minyomerus* [JF2015].** The monophyly of *Minyomerus* [JF2015] is well supported by eight unreversed synapomorphies (Fig. 49; Bremer support value [henceforth: bsv] = 9, Bootstrap support [henceforth: boot] = 96): (1) the appressed scales are sub-circular and overlap posteriorly (character 1, state 2 [char. 1:2]); (2) the nasal plate is a broad, scale-covered, chevron-shaped ridge demarcating the epistoma (char. 10:2); (3) a sulcus posteriad of nasal plate is present (char. 12:1); (4) the scrobe is subequal in length to funicle and club combined (char. 14:1); (5) the head is directed slightly ventrad (char. 17:2); (6) the metatibial apexlacks setiform bristles, and instead has bristles that are shorter to subequal in length to surrounding setae and conical (char. 22:1); (7) the mesotarsi are slightly shorter than the mesotibiae (char. 24:2), and (8) all tarsi lack pads of setiform setae, and instead have stout spiniform setae (char. 25:1). An additional homoplasious trait supports the monophyly of the genus, namely, the intercoxal process is medially divided (char. 18:1). Other traits useful in diagnosing *Minyomerus* [JF2015] include (compare with Anderson 2002): (1) the elytral humeri are rounded, rather than angled (char. 26:1); (2) the profemora are not dilated (char. 20:1); (3) the prothoracic legs lack femoral spines; (4) the protibiae are ventrally excavated by a longitudinal groove or concavity (char. 21:1); (5) a distinct scrobe channel is present, and directed ventrad of the eye; with (6) a more or less apparent tooth formed by an overhang of the dorsal margin.

More extensive outgroup sampling is needed to appropriately place *Minyomerus* [JF2015] in phylogenetic relationship to all genera presently assigned to the Tanymecini [non-focal] (Alonso-Zarazaga and Lyal 1999), including psammophilic lineages presumed to have evolved under strong selective pressures in these harsh desert environments. Anderson’s (2002) key identifies the genus *Miloderoides* van Dyke, 1936 sec. Anderson (2002), in the couplet immediately preceding *Minyomerus* sec. Anderson (2002) and *Piscatopus* sec. Anderson (2002) in reference to the presence of spiniform setae ventrally on the tarsi. However, such spiniform setation is a common trait in psammophile weevils, and not necessarily indicative of close phylogenetic relationship. Similarly, *Isodacrys* sec. Anderson (2002) keys out closely to *Minyomerus* sec. Anderson (2002) by virtue of the rounded elytral humeri. This reduction in the humeral angle is also likely homoplasious and often correlated with the secondary loss of the wings in entimine weevils (Franz 2012).

**Intrageneric relationships.** Within *Minyomerus* [JF2015], several clades are recognized based on variably strong support, as follows (Fig. 49). The *Minyomerus conicollis*–*Minyomerus rutellirostris* clade [JF2015] is supported by two unreversed synapomorphies (bsv = 2; boot = 55); viz. the laminar arms of the spiculum ventrale are not mesally joined, and are clearly bifurcate (chars. 30:1, and 31:1, respectively). These characters reflect a deep bifurcation within *Minyomerus* [JF2015], as the sister *Minyomerus imberbus*–*Minyomerus laticeps* clade [JF2015] (bsv = 1; boot = 96) corresponds to the genus *Elissa* sec. Casey (1888). The sister relationship *Minyomerus constrictus* [JF2015] and *Minyomerus laticeps* [JF2015] is well supported by genital morphology, especially by distinctive hexagonal shape of the prementum (char. 7:1) and the form of the lamina of sternum VIII. Other characters aligned with the monophyly of this clade include the relatively large eyes and presence of a well-developed tuft of post-ocular vibrissae. However, in light of the weak and frequently homoplasious characters supporting the *Minyomerus imberbus*–*Minyomerus laticeps* clade [JF2015], and much stronger clade support for an inclusive circumscription of *Minyomerus* [JF2015] (see above), we consider resurrecting *Elissa* Casey (1888) as unwarranted.

The *Minyomerus languidus*–*Minyomerus rutellirostris* clade [JF2015] is supported by a single, unreversed synapomorphy (bsv = 2; boot = 55), viz., the anterolateral margins of the prementum are simple, and not expanded and angular laterad of the ligula (char. 6:1). Additionally, three supporting characters exhibit one or more reversals (chars. 4:1, 5:1, and 46:0). The anterior margin of the ligula is flat and forms a very distinct face, and is strongly ligulate, having a typical pentagonal appearance. This clade roughly corresponds to the genus *Pseudelissa* sec. Casey (1888), and is further supported by the reduced tuft of post-ocular vibrissae and relatively small eyes. The clade does not include *Minyomerus conicollis* [JF2015], which was originally placed into *Minyomerus* sec. Green (1920). The original type of *Pseudelissa* sec. Casey (1888), *Pseudelissa cinerea* sec. Casey (1888), is a junior synonym of *Minyomerus languidus* [JF2015]. Thus *Pseudelissa* sec. Casey (1888) remains in synonymy of *Minyomerus* [JF2015].

The *Minyomerus aeriballux*–*Minyomerus rutellirostris* clade [JF2015] is supported by an unreversed synapomorphy (bsv = 1; boot = 100); i.e., the nasal plate is bordered by a relatively more strongly defined, punctate sulcus (char. 12:2). The clade is also supported by having the terminal funicular segment at least partially clothed with broad scales (char. 16:1), with an inferred reversal in the *Minyomerus gravivultus*–*Minyomerus rutellirostris* clade [JF2015]. This conspicuous scaling is often, but not always, correlated with an apparent loss of the thinner, more setiform scales present on the terminal funicular segment in other taxa (char. 15:1). Additionally, the *Minyomerus aeriballux*–*Minyomerus rutellirostris* clade [JF2015] is supported by a symplesiomorphy shared with its sister taxon, *Minyomerus microps* [JF2015], i.e., the ligula projects beyond and ‘hides’ the first segment of the labial palps in ventral view (char. 8:1).

Within the *Minyomerus aeriballux*–*Minyomerus rutellirostris* clade [JF2015], each internal node is supported by at least one unreversed synapomorphy. A basal split separates this clade into two groups: the *Minyomerus aeriballux*–*Minyomerus trisetosus* clade [JF2015] and the *Minyomerus puticulatus*–*Minyomerus rutellirostris* clade [JF2015]. The *Minyomerus aeriballux*–*Minyomerus trisetosus* clade [JF2015] is characterized by a single unreversed synapomorphy, namely, the lack of a medial, anteriorly directed, sclerotized process on the coxites of the ovipositor (char. 34:1). This character is phylogenetically significant, given the importance and relatively conserved morphology of the ovipositor within *Minyomerus* [JF2015]. The *Minyomerus puticulatus*–*Minyomerus rutellirostris* clade [JF2015] is primarily characterized by the sternum VIII which has apically bifurcating arms between which the disc of the lamina is medially less sclerotized than lateral regions (char. 21:1).

Within the *Minyomerus aeriballux*–*Minyomerus trisetosus* clade [JF2015] there is a basal division into (1) the *Minyomerus aeriballux*–*Minyomerus reburrus* clade [JF2015], characterized by three unreversed synapomorphies (chars. 2, 9, and 22; bsv = 2; boot = 56), and (2) the *Minyomerus caseyi*–*Minyomerus trisetosus* clade [JF2015], which is supported by a single unreversed synapomorphy (char. 32; bsv = 3; boot = 68) and five homoplasious characters (chars. 3, 4, 5, 35, and 38). The *Minyomerus aeriballux*–*Minyomerus reburrus* clade [JF2015] is delimited by: (1) having copious setae that are not arranged in even, single-file rows on the elytra; (2) the longitudinally rounded, rather than protuberant frons, which gives the head a uniquely conical appearance; and (3) the setation of the metatibial apexwhich is covered in longer, translucent, lighter colored, and somewhat lamelliform setae, rather than the typical, opaque brown, conical setae present throughout the genus. This clade is further supported by the rather large and protruding eyes, which do not exhibit the degree of impression seen in many other *Minyomerus* [JF2015]. The sister *Minyomerus cracens*–*Minyomerus trisetosus* clade [JF2015] is primarily characterized by a character of the male genitalia (char. 40:1). The species *Minyomerus caseyi* [JF2015] and *Minyomerus trisetosus* [JF2015] are sister in light of the shared presence of a unique lateral, anteriorly-directed, recurved, alate process on the coxites of the ovipositor (char. 32:1).

The major *Minyomerus puticulatus*–*Minyomerus rutellirostris* clade [JF2015] is further bifurcated into two multi-species sister clades. First among these, the *Minyomerus puticulatus*–*Minyomerus politus* clade [JF2015] is well delimited by having the spematheca with a cylindrical bulb located apically on the corpus (char. 27:1; bsv = 2; boot = 67). The clade is also characterized by two other synapomorphies; i.e., the anterior margin of tergum VII is sclerotized fully, appearing as an obviously complete band (char. 33:1), and the lamina of the spiculum gastrale is longer than broad and anteriorly extended along the syle (char. 39:1). Both characters are reversed in *Minyomerus bulbifrons* [JF2015], but nevertheless the clade is well supported by the derived and unique bulb on the corpus of the spermatheca. The *Minyomerus bulbifrons*–*Minyomerus politus* clade [JF2015] is sustained by three unreversed synapomorphies (chars. 13:1, 37:1, and 44:1; bsv = 2; boot = 60) and a single homoplasious character (char. 15:1). Characters most indidicative of their close relationship are the very strongly protuberant frons, the elongate-spiriform apical sclerite of the aedeagal flagellum, and the form of the basally broad, evenly tapering aedeagal pedon. The form of the apical sclerite is interesting in that the spiriform sclerites display chirality. The sclerite is a left-handed helix in *Minyomerus bulbifrons* [JF2015], and a right-handed helix in *Minyomerus politus* [JF2015]. Given their overlapping distributions and similar appearance, the origin of these species may be related to sexual selection, representing the outcome of reproductive isolation due to mutations affecting the development of the aedeagal flagellum and sclerites.

The second, remaining sister to the *Minyomerus puticulatus*–*Minyomerus politus* clade [JF2015] is the *Minyomerus gravivultus*–*Minyomerus rutellirostris* clade [JF2015]. The sole yet uniquely significant synapomorphy for the latter clade is the presence of an unsclerotized, membranous region between the bifurcating arms of the lamina of sternum VII (char 29:1). Two homoplasious characters provide additional support; i.e., the faintly impressed or unimpressed nasal plate (char. 11:1) and the absence of broad scales on the terminal funicular segment (char. 16:0). The *Minyomerus griseus*–*Minyomerus rutellirostris* clade [JF2015] is sister to *Minyomerus gravivultus* [JF2015], and well supported by two unreversed synapomorphies (chars. 19 and 42; bsv = 5; boot = 71) and three homoplasious characters (chars. 5, 23, and 46). Hence the critical characters for this clade are: (1) the intercoxal process is divided at the midpoint between the coxae, but has both the anterior and posterior processes extending completely between the procoxae and contiguous with each other; and (2) the male tergum VII is nearly 4 × as long as broad.

**Classificatory emendations.** Sleeper (1960) erected the monotypic genus *Piscatopus* sec. Sleeper (1960) in recognition of the complete separation of the procoxae by the intercoxal process (char. 19), as opposed to incomplete separation present in other members of *Minyomerus* sec. Sleeper (1960), as well as the more globular pronotum. Additional characters included the small overhanging tooth on the dorsal margin of the scrobe, having elytral stria 10 absent in basal third, and smaller mandibles. However, these characters are not adequate for differentiating *Piscatopus* sec. Sleeper (1960) from *Minyomerus* [JF2015], whose members (as herein described) exhibit varying degrees of separation of the procoxae. The procoxal separation can be more or less complete, even within *Minyomerus griseus* [JF2015]. The inferred sister species, *Minyomerus rutellirostris* [JF2015], shares this variable trait. We also note that the separation is significantly less conspicuous in the males of these species, where only the tips of the projecting intercoxal processes appear to meet between the procoxae. The shape of the pronotum, while somewhat more globular and enlarged than in some other species, is not exceptional, and bears similarity to the globular pronotum of the type species of *Minyomerus microps* [JF2015]. Furthermore, when cleared in 10% KOH, elytral stria 10 is fully apparent and complete along its entire length, and the size of mandibles is within the range of variation of other *Minyomerus* [JF2015] members. The species *Minyomerus griseus* [JF2015] and *Minyomerus rutellirostris* [JF2015] form a clade sister to *Minyomerus gravivultus* [JF2015], sharing a greatly shortened and widened tergum VII (char. 42). Moreover, the *Minyomerus gravivultus*–*Minyomerus rutellirostris* clade [JF2015] is supported by having a medially membranous region on the lamina of sternum VIII, around which the laminar arms of the spiculum ventral rejoin posteriad of their basal bifurcation (char. 29). Jointly these characters justify invalidation of *Piscatopus* sec. Sleeper (1960) to junior synonymy of *Minyomerus* [JF2015], renaming *Minyomerus griseus* [JF2015] accordingly as a new combination.

We also establish the synonymy of two entities *Minyomerus innocuus* sec. Horn (1876) and *Minyomerus microps* [JF2015]. *Thylacites microps* sec. Say (1831) was described therein based on a specimen collected during an excursion to the Rocky Mountains and the tributaries of the Missouri River in 1819. This specimen was sent to Schoenherr on March 18, 1830 (Jens Prena, pers. comm.), and then redescribed by Boheman, who was unaware of Say’s (1831) description, in Schoenherr (1833), as *Thylacites microsus* sec. Boheman (1833). The original owner of the specimen is referred to in Schoenherr (1833: 523): “*Curculio microsus*. SAY *in Litt.*/ *Patria* : Missouri Americae borealis. Mus. Dom. SAY”. While compiling “The complete writings of Thomas Say”, LeConte (1859: 268) recognized the homotypic synonymy and remarked: “This is *Thylacites microsus* Sch. – LEC.” Even though *Thylacites microps* sec. LeConte (1859) was thereby subsequently given priority, apparently this name was never used in identificationsof other specimens after 1859.

Horn (1876) subsequently described *Minyomerus* sec. Horn (1876) and two entailed species, *Minyomerus innocuus* sec. Horn (1876) and *Minyomerus languidus* sec. Horn (1876). His description of *Minyomerus innocuus* sec. Horn (1876) was based on a single specimen from Colorado. The name *Minyomerus innocuus* has been in regular use for identifications since that time, and was designated as the type of *Minyomerus* sec. Pierce (1913: 400), likely due to the order in which the species descriptions appear in the text. It was not until the publication of Blackwelder and Blackwelder (1948: 46), on the authority of Buchanan (“in litt.”), that *Thylacites microps* Say sec. Blackwelder & Blackwelder (1948) was recognized as an available (yet unused) name for a member species of *Minyomerus* sec. Blackwelder & Blackwelder (1948). Evidently Buchanan (in litt.) failed to recognize that *Minyomerus innocuus* sec. Horn (1876) and *Thylacites microps* sec. Say (1831) are heterotypic synonyms and that their respective types pertain to the same species (or congruent species-level concepts). Our analysis of the images of these types reveal their nearly identical curvature of the rostrum in lateral view; the diagnostic broad sculpture of the elytra and elytral intervals; shared body proportions which are characteristically robust; the similar form of the pronotum, which is somewhat globular and slightly narrower anteriorly; as well as the overall similarity of body form, scaling, setation, size, and overlapping species distributions. We therefore invalidate *Minyomerus innocuus* sec. Horn (1876) to junior synonymy of *Minyomerus microps* [JF2015].

**Figure 50.**
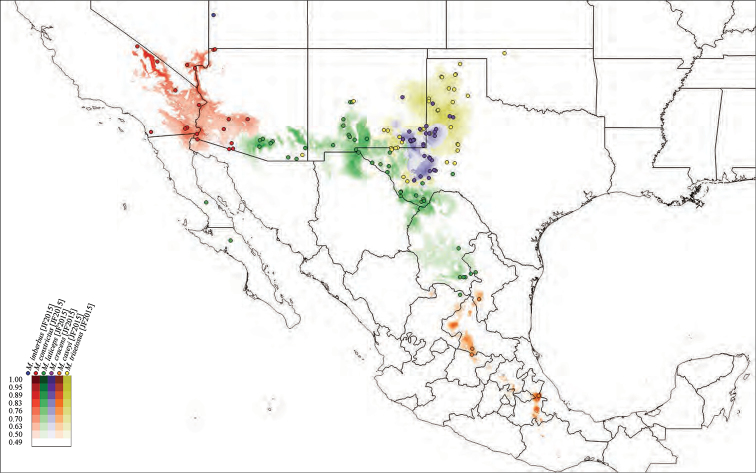
Combined occurrence record and Maxent habitat modeling map for si× species of *Minyomerus* [JF2015], as indicated in the legend. Increasing saturation indicates an increased probability of successful prediction of presence. Colored circles indicate georeferenced localities for a given species. For some species, only locality data are shown (see Methods section for further detail details).

**Figure 51.**
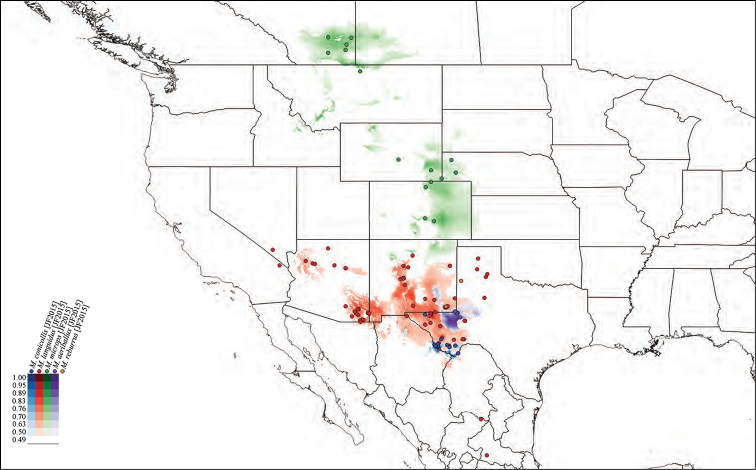
Combined occurrence record and Maxent habitat modeling map for five species of *Minyomerus* [JF2015], as indicated in the legend. See also Fig. 50.

**Figure 52.**
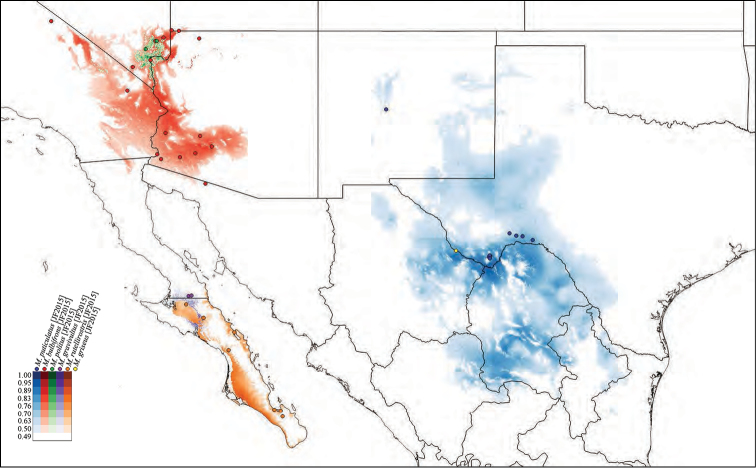
Combined occurrence record and Maxent habitat modeling map for five species of *Minyomerus* [JF2015], as indicated in the legend. See also Fig. 50.

**Biogeographic relationships.** The present-day distribution and phylogeny of members of *Minyomerus* [JF2015] suggest a complexpattern of radiation through the North American continent. As documented and modeled herein (Figs 50–52), the primary occurrence of *Minyomerus* [JF2015] to western North America, with most of the species diversity observed in the desert southwest habitats, suggests an origin in this region and ecological context.

Knowledge of the timing of historical divergence events is critical for correlating species radiations with presumed evolutionary driving factors and historical biogeographic scenarios (Franz and Engel 2010); however, such an analysis is hampered by the absence of an age-calibrated phylogeny for *Minyomerus* [JF2015]. The lack of relevant, closely related tanymecine [non-focal] fossils is particularly problematic. The oldest tanymecine [non-focal] fossil is known from the Upper Eocene in Chinese amber (> 36.6 mya; Hong 2002). Slightly younger Amber specimens are also recorded from the Lower Oligocene of Colorado and Europe (Yunakov and Kirejtshuk 2011), yet their phylogenetic relationships to *Minyomerus* [JF2015] remain unexplored. The following discussion must therefore remain somewhat speculative.

The Chihuahuan Desert is frequently invoked as the point of origin for modern North American desert taxa, as this region is considered to have been climatically stable since the Middle Miocene (ca. 15 mya; see Wilson and Pitts 2010). This area is central to the distribution of *Minyomerus* [JF2015] (Figs 50–52). The diversity of phenotypic traits and wide-ranging, overlapping distributions of many species may be indicative of long evolutionary history that followed the development of these desert biomes. Early members of *Minyomerus* [JF2015] possibly diversified originated following the rise of the Sierra Madre Occidental in the Miocene, 15-7 mya, during the Neogene uplift. This event is thought to have altered the relatively tropical climate of North America by blocking moisture from the Pacific Ocean and Gulf of Mexico, causing the Great Plains region and the Mexican Plateau to become significantly more arid (Wilson and Pitts 2010). *Minyomerus* [JF2015] is currently restricted to semi-/arid habitats, and is psammophilic, showing several putative adaptations for sandy habitats such as stout, spiniform setae on the tarsi which are thought to be correlated with sandy substrates (Fet et al. 1998). We therefore consider it most likely that *Minyomerus* [JF2015] diversified in correlation with shifts from moist to more arid climates and sandy habitats, approximately 15 mya.

The Sonoran Desert is thought to have become steadily more arid between 15-8 mya (van Devender 2000). These developments may correspond to the basal split between the *Minyomerus imberbus*–*Minyomerus laticeps* clade [JF2015] and the *Minyomerus conicollis*–*Minyomerus rutellirostris* clade [JF2015] (Fig. 49). The species *Minyomerus constrictus* [JF2015] and *Minyomerus imberbus* [JF2015] inhabit desert regions west of the Colorado Plateau and the Rocky Mountains, whereas *Minyomerus laticeps* [JF2015] is more widespread, occurring in the Sonoran and Chihuahan deserts (Fig. 50). Under this scenario the *Minyomerus imberbus*–*Minyomerus laticeps* clade [JF2015] dispersed northwest from the Mexican Plateau and speciated along with the developing desert biomes. *Minyomerus imberbus* [JF2015] may have diverged into the newly formed Great Basin Desert (ca. 8 mya). The split between *Minyomerus constrictus* [JF2015] and *Minyomerus laticeps* [JF2015] into their present distributions – the Mojave Desert and the Sonoran and Chihuahuan Deserts, respectively – corresponds to the formation of the Bouse Embayment, a northern extension of the Gulf of California, approximately 8–4 mya. The changing water regimes and geographic connections of this region (Wilson and Pitts 2010) may account for the present-day, disjoint distribution of *Minyomerus laticeps* [JF2015] solely in Baja California Sur.

*Minyomerus conicollis* [JF2015] is found in higher elevations and cooler temperatures, reaching north into Texas, whereas *Minyomerus languidus* [JF2015] occurs more westward, ranging from Texas to Arizona (Fig. 51). The present-day distribution of *Minyomerus microps* [JF2015] (Fig. 51) suggests parallel dispersal northward, with *Minyomerus imberbus* [JF2015] occurring west of the Rocky Mountains and *Minyomerus microps* [JF2015] east. The timing of these radiation events is possibly correlated with the formation of the Llano Estacado during the Neogene uplift (Matthews 1969; Spearing 1991). We note that *Minyomerus languidus* [JF2015] and *Minyomerus microps* [JF2015] are considered parthenogenetic.

The *Minyomerus aeriballux*–*Minyomerus trisetosus* clade [JF2015] occurs predominantly in west-central Texas and eastern New Mexico, whereas members of the *Minyomerus puticulatus*–*Minyomerus rutellirostris* clade [JF2015] inhabit regions across southern Texas, central New Mexico, southeastern Arizona, California, Nevada, and Baja California. It appears that this split occurred along an irregular north-south boundary. The species *Minyomerus aeriballux* [JF2015], *Minyomerus reburrus* [JF2015], *Minyomerus cracens* [JF2015], and *Minyomerus trisetosus* [JF2015], all have slightly to mostly overlapping distributions. The species *Minyomerus aeriballux* [JF2015] and *Minyomerus reburrus* [JF2015] (Fig. 51) are apparently more psammophilic than the members of the *Minyomerus cracens*–*Minyomerus trisetosus* clade [JF2015], and associated with very sandy soils and dune fields. The split between *Minyomerus cracens* [JF2015] and the *Minyomerus caseyi*–*Minyomerus trisetosus* clade [JF2015] is congruent with a vicariance scenario, since *Minyomerus cracens* [JF2015] occurs further north and west than *Minyomerus caseyi* [JF2015] and *Minyomerus trisetosus* [JF2015], respectively (Fig. 50). Parthenogenesis has likely been acquired independently in two taxa of this clade: *Minyomerus reburrus* [JF2015] and *Minyomerus trisetosus* [JF2015]. The sister species *Minyomerus caseyi* [JF2015] and *Minyomerus trisetosus* [JF2015] are geographically separated by the Sierra Madre Oriental and Chisos Mountains (Fig. 50).

Species distributions and biogeographic relationships in the *Minyomerus puticulatus*–*Minyomerus griseus* clade [JF2015] are complex. The species *Minyomerus puticulatus* [JF2015] is known from southeast Arizona, central New Mexico (Sevilleta), and Big Bend National Park (Fig. 52). *Minyomerus bulbifrons* [JF2015] occurs throughout the Mojave Desert; *Minyomerus politus* [JF2015] is known only from Clark County, Nevada; and *Minyomerus gravivultus* [JF2015] and *Minyomerus rutellirostris* [JF2015] are endemic to Baja California (Fig. 52). Lastly, *Minyomerus griseus* [JF2015] is only known from the Chihuahuan Desert near Presidio, Texas (Fig. 52). The divergence between *Minyomerus puticulatus* [JF2015] and the *Minyomerus bulbifrons*–*Minyomerus politus* clade [JF2015] might reflect dispersal by the latter into the Mojave Desert. The split between *Minyomerus bulbifrons* [JF2015] and *Minyomerus politus* [JF2015] is likely very recent; the two species are very similar in exterior and genitalic morphology but display chirality in the curvature of the apical sclerite of the aedeagal flagellum. The biogeographic relationships within the *Minyomerus gravivultus*–*Minyomerus rutellirostris* clade [JF2015] are possibly obscured by their present-day distributions. Both *Minyomerus gravivultus* [JF2015] and *Minyomerus rutellirostris* [JF2015] are endemic to Baja California, whereas *Minyomerus griseus* [JF2015] is found in the Chihuahuan Desert near the Big Bend region of Texas. The disparity between the current ranges of *Minyomerus griseus* [JF2015] and *Minyomerus rutellirostris* [JF2015] is remarkable, and would be explicable by postulating either: (1) a widespread ancestor that became separated by the formation of the Baja California Peninsula, followed by subsequent extinctions between the Peninsula and the Big Bend region; or (2) dispersal by the ancestor of *Minyomerus griseus* [JF2015] from Baja California to mainland Mexico, followed by further dispersal north to match thepresent-day distribution. The latter scenario seems less likely, considering possible barrier effects the Sierra Madre Occidental to the dispersal of these small, flightless weevils.

**Evolution of life history traits and parthenogenesis.** All species of *Minyomerus* [JF2015] are flightless, and many have been observed on a wide range of plant hosts. The adults are exclusively leaf-feeding, and found on the branches of their hosts during the day and night. Field work has revealed *Minyomerus* [JF2015] weevils to frequently occur in patches of relatively high population density. In certain habitats they are found in densities so high that every plant in the vicinity has dozens or more individuals present while in an adjacent region, less than half a mile away, there may be none. This phenomenon was observed at one site in Nevada, northeast of Las Vegas. In the area with abundant *Minyomerus* [JF2015] populations, the apparent damage to the host (creosote bush [non-focal]) is minor and not noticeably different from other areas. Under such circumstances, selective pressures favoring dispersal may not be high. Conversely, high population density can also result from poor dispersal ability, or a correlate of aggregating behavior, as seen in *Sitona* [non-focal] (Blight et al. 1984).

Loss of the hindwings is often correlated with an increase in fecundity, presumably due to a reduced expenditure of energy on relatively costly flight structures (Roff 1986, 1990; Wagner and Liebherr 1992). The loss of flight is also correlated with stable environments, such as deserts, where dispersal is not essential to survival and where the benefits of increased fecundity outweigh the costs of reduced dispersal potential. Similarly, the development of parthenogenesis and polyploidy is more frequent among flightless insects than flying insects (Roff 1990). No enrgy is invested into mating rituals. In stable environments, low genetic variability may result in greater success of progeny similar to reproductively successful parents.

According to the inferred phylogeny for *Minyomerus* [JF2015] (Fig. 49), parthenogenetic reproduction has evolved at least twice independently within the genus. The species *Minyomerus languidus* [JF2015], *Minyomerus microps* [JF2015], and *Minyomerus trisetosus* [JF2015] are only known to have females. Under a most parsimononious ACCTRAN scenario, we would have to postulate a reversal of this trait following the divergence of *Minyomerus microps* [JF2015]. This scenario is not cytologically very plausible (though not impossible either). It is therefore more likely that the gains of parthenogenetic reproduction observed in *Minyomerus languidus* [JF2015] and *Minyomerus microps* [JF2015] occurred convergently and in relation to sexually reproducing ancestors (see also Saura et al. 1993; Normark and Lanteri 1998).

Saura et al. (1993) list 75 different parthenogenetic lineages of weevils. All of these are known to be apomictic, and the majority are polyploid. Apomictic parthenogenesis occurs when meiosis becomes suppressed, resulting in egg development through mitosis, which may exhibit some vestigial aspects of meiosis. The authors found that development of this form of reproduction varies in origin, but is dependent on polyploidization. The process includes the acquisition of triploidy through hybridization, i.e., the fertilization of a hybrid diploid gamete with a haploid gamete produces triploid offspring, a phenomenon known as autopolyploidization and observed (e.g.) in the bagworm (Psychidae [non-focal]) moth *Dahlica triquetrella* [non-focal] (Huebner, 1813). Most instances of weevil parthenogenesis are though to be the result of hybridization, resulting in triploidy, and followed by subsequent changes in ploidy number (Saura et al. 1993). It is also possible that the putative parthenogenetic species of *Minyomerus* [JF2015] are the outcome of hybridization events between closely related species, given the numerous instances of overlapping species distributions (see above). Further studies should focus on understanding the extent and underlying mechanisms of parthenogenesis in different *Minyomerus* [JF2015] lineages (e.g., via karyotyping of eggs), and on utilizing the findings to attain a deeper understanding of the historical interactions of biogeographic and reproductive factors leading to the present-day diversity and distribution of *Minyomerus* [JF2015] species.

## Supplementary Material

XML Treatment for Minyomerus [JF2015]

XML Treatment for Minyomerus imberbus [JF2015]

XML Treatment for Minyomerus constrictus [JF2015]

XML Treatment for Minyomerus laticeps [JF2015]

XML Treatment for Minyomerus conicollis [JF2015]

XML Treatment for Minyomerus languidus [JF2015]

XML Treatment for Minyomerus microps [JF2015]

XML Treatment for Minyomerus aeriballux [JF2015]

XML Treatment for Minyomerus reburrus [JF2015]

XML Treatment for Minyomerus cracens [JF2015]

XML Treatment for Minyomerus caseyi [JF2015]

XML Treatment for Minyomerus trisetosus [JF2015]

XML Treatment for Minyomerus puticulatus [JF2015]

XML Treatment for Minyomerus bulbifrons [JF2015]

XML Treatment for Minyomerus politus [JF2015]

XML Treatment for Minyomerus gravivultus [JF2015]

XML Treatment for Minyomerus griseus [JF2015]

XML Treatment for Minyomerus rutellirostris [JF2015]

## Supplementary materials

Supplementary material 1 Set of 13 Euler/X toolkit input data files Data type: Euler/X taxonomy alignment input files (.txt) Explanation note: Set of 13 Euler/X toolkit input data files for all alignments produced for *Minyomerus* [JF2015] use case (Figs 3, 4). Each file is saved in txt format and contains annotations and instructions for run commands to yield the alignments and visualizations shown in the corresponding figures. Dryad. doi: 10.5061/dryad.53mv1 This dataset is made available under the Open Database License (http://opendatacommons.org/licenses/odbl/1.0/). The Open Database License (ODbL) is a license agreement intended to allow users to freely share, modify, and use this Dataset while maintaining this same freedom for others, provided that the original source and author(s) are credited.M. Andrew Jansen, Nico M. Franz

Supplementary material 2 Set of 13 Euler/X toolkit output Maximally Informative Relations Data type: Euler/X taxonomy alignment output files (.csv,. pdf) Explanation note: Set of 13 Euler/X toolkit output Maximally Informative Relations (MIR) and alignment visualizations for the input data files provided in Suppl. material 1. Each output file is saved in. csv (MIR) and. pdf (alingments) format, respectively. Dryad. doi: 10.5061/dryad.53mv1 This dataset is made available under the Open Database License (http://opendatacommons.org/licenses/odbl/1.0/). The Open Database License (ODbL) is a license agreement intended to allow users to freely share, modify, and use this Dataset while maintaining this same freedom for others, provided that the original source and author(s) are credited.M. Andrew Jansen, Nico M. Franz
